# Antineoplastics Encapsulated in Nanostructured Lipid Carriers

**DOI:** 10.3390/molecules26226929

**Published:** 2021-11-17

**Authors:** Gustavo Henrique Rodrigues da Silva, Ludmilla David de Moura, Fabíola Vieira de Carvalho, Gabriela Geronimo, Talita Cesarim Mendonça, Fernando Freitas de Lima, Eneida de Paula

**Affiliations:** Department of Biochemistry and Tissue Biology, Institute of Biology, University of Campinas—UNICAMP, Campinas 13083-862, Brazil; gustavohrs@gmail.com (G.H.R.d.S.); ludmilladavidm@gmail.com (L.D.d.M.); fabiolavieiracarvalho@hotmail.com (F.V.d.C.); gabrielageronimo95@gmail.com (G.G.); talitacesarim@yahoo.com.br (T.C.M.); flfernando_@hotmail.com (F.F.d.L.)

**Keywords:** antineoplastics, drug delivery, nanostructured lipid carriers, cancer

## Abstract

Ideally, antineoplastic treatment aims to selectively eradicate cancer cells without causing systemic toxicity. A great number of antineoplastic agents (AAs) are available nowadays, with well-defined therapeutic protocols. The poor bioavailability, non-selective action, high systemic toxicity, and lack of effectiveness of most AAs have stimulated the search for novel chemotherapy protocols, including technological approaches that provide drug delivery systems (DDS) for gold standard medicines. Nanostructured lipid carriers (NLC) are DDS that contain a core of solid and lipid liquids stabilised by surfactants. NLC have high upload capacity for lipophilic drugs, such as the majority of AAs. These nanoparticles can be prepared with a diversity of biocompatible (synthetic or natural) lipid blends, administered by different routes and functionalised for targeting purposes. This review focused on the research carried out from 2000 to now, regarding NLC formulations for AAs (antimetabolites, antimitotics, alkylating agents, and antibiotics) encapsulation, with special emphasis on studies carried out in vivo. NLC systems for codelivery of AAs were also considered, as well as those for non-classical drugs and therapies (natural products and photosensitisers). NLC have emerged as powerful DDS to improve the bioavailability, targeting and efficacy of antineoplastics, while decreasing their toxic effect in the treatment of different types of cancer.

## 1. Introduction

### 1.1. Antineoplastics

Cancer is the widespread name for a large group of diseases characterised by the uncontrolled proliferation of abnormal cells and further spread to organs or tissues [1]. Chemotherapy is claimed to have started in 1900, when the German chemist Paul Ehrlich tested several compounds for treating cancer in animals [2]. However, it was in just 1942 that the first chemical for inducing tumour regression (nitrogen mustard, used systemically) was tested for the first time in humans [3]. Currently, countless drugs for cancer treatment, the so-called antineoplastic agents (AAs), are used for the treatment of several kinds of cancer [4], benefitting patients not only for tumour regression but also for decreasing recurrence and metastasis beyond increasing survival.

**Figure 1:**
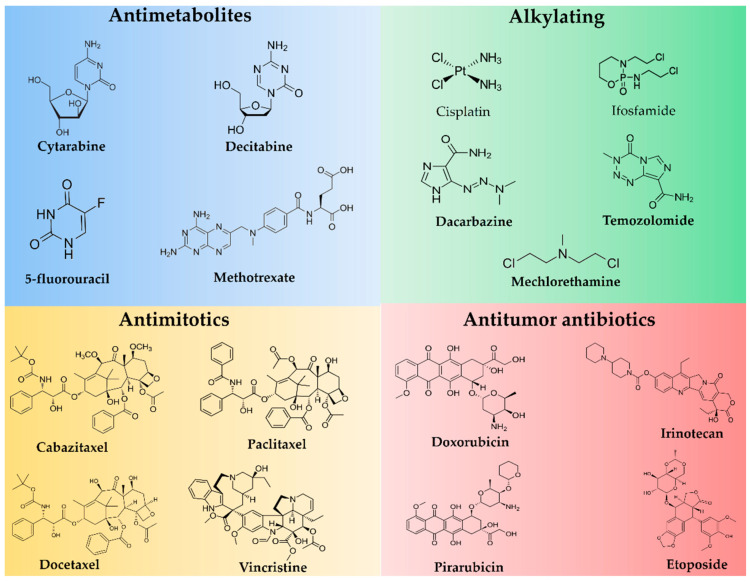
Chemical structures of antineoplastics agents described in the main text.

The classification of AAs is not consensual, considering the diversity of structures and mechanisms of action. While some authors propose a classification with as many as 16 classes, including antimitotic agents, protein kinase inhibitors, monoclonal antibodies, and miscellaneous [5], the major AAs classes are antimetabolites, alkylating agents, and natural products, including antibiotics and hormones/antagonists [6] (Figure 1). Although there is a discussion in the literature regarding the criteria for AAs grouping, their indication for each kind of cancer is well established.

Despite their well-established uses, AAs are mostly highly toxic agents. In this way, research is still needed in order to find more selective molecules or alternative approaches to reduce the toxicity and/or increase the efficiency of the existing AAs [7]. Among the limitations of AAs are low bioavailability, non-specific body distribution, low tumour delivery, hepatotoxicity, and nephrotoxicity [8]. To face this, nanotechnological approaches such as the development of drug delivery systems (DDS) have been pursued, including nanostructured lipid carriers (NLC).

### 1.2. Nanostructured Lipid Carriers

Lipid-based nanoparticles are DDS consisting of a lipid matrix stabilised by surfactants that are very useful for the encapsulation of hydrophobic drugs [9]. The lipid matrix composition defines their subtypes. Those composed of 100% liquid lipids are called nanoemulsions, whereas those composed of 100% solid lipids (at room and body temperature [10]) are called solid lipid nanoparticles (SLN). Nanostructured lipid carriers (NLC) evolved from SLN but have a mixture of solid and liquid lipids. Such lipid blends are responsible for the structural stability of NLC, avoiding lipid crystallisation without expelling the encapsulated drug [11]. Liquid lipids also increase drug solubility, justifying the greater upload capacity of NLC over SLN. Since the lipid core comprises the major excipients of these nanoparticles, in recent years, NLC have been classified into several types (core–shell, smart lipids, and flip flop [12,13]), depending on the solid/liquid lipid ratio that determines the physicochemical characteristics of the nanostructure, as well as its drug interactions and in vivo actions [10,12].

### 1.3. Metrics Regarding Articles with Antineoplastics Loaded in NLC

**Figure 2:**
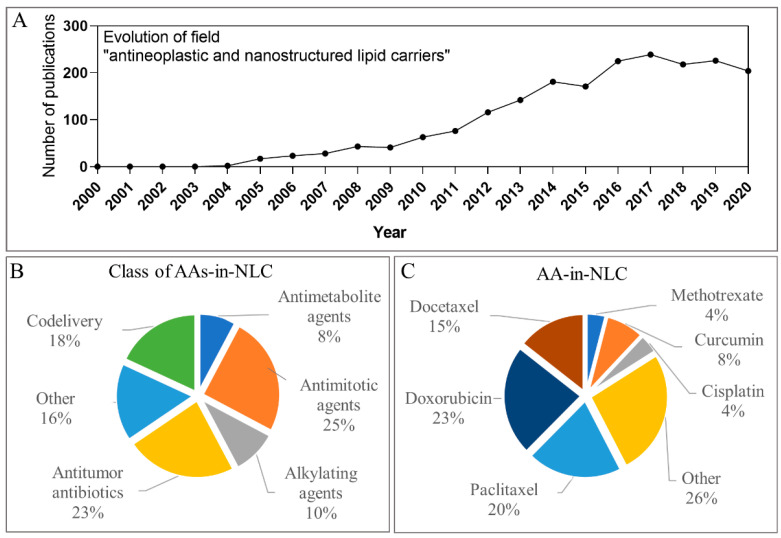
(**A**) Publications in PubMed platform relating the terms antineoplastics and nanostructured lipid carriers from 2000 to 2020; (**B**) distribution of the analysed articles regarding the class of antineoplastic agent (AA); (**C**) distribution of the analysed articles regarding the antineoplastic agent.

First, to understand the relevance of these AAs-in-NLC, we conducted a search with the descriptors “antineoplastics and nanostructured lipid carriers” in the PubMed platform (pubmed.gov (accessed on 11 November 2021)). Figure 2A shows the evolution of publications in this field, beginning in 2000 and up to 2020. It is evident that just a few years after the description of NLC in the literature (1999 [11]), the number of reports of AAs-in-NLC started to grow steadily (2004–2010) and then exponentially in the last decade, reaching its maximum in 2017. The significant increase in the number of publications (>200/year since 2016) indicates the potential of NLC for the encapsulation of AAs and cancer treatment.

This review covers the reports of NLC formulations containing AAs with data on the in vitro and/or in vivo performance from January 2000 to July 2021 using several research platforms. All the reviewed articles are compiled in Table S1. Therefore, NLC formulations in the literature have been classified according to the mechanism of action of the encapsulated AAs: antimetabolites, antimitotics, alkylating agents, and antibiotics (see Table S1). No reports on hormonal AAs-in-NLC, and just one (two) case(s) of codelivery systems involving protein kinase inhibitors (monoclonal antibodies) were found. The vast majority of the articles focused on the encapsulation of gold standard AAs. Antimitotics (25%) and antibiotics (23%) had the highest number of articles, followed by alkylating agents (10%) and antimetabolites (8%) (Figure 2B). As shown in Figure 2C, the three most studied AAs were doxorubicin (23%), paclitaxel (20%) and docetaxel (15%).

An interesting advantage of NLCs are their versatility to be applied by different routes (oral, subcutaneous, intramuscular, intravenous, nasal and topical [9]). Thus, it is interesting to note that, although most of the AAs approved so far are administered intravenously (requiring a hospital environment), several AAs-in-NLC studies have explored the use of different routes of administration to facilitate the treatment. Among these, formulations designed for oral [14,15,16,17,18,19] or nasal [20] administration have shown promising results, improving the bioavailability of AAs with less systemic toxic effects.

**Figure 3:**
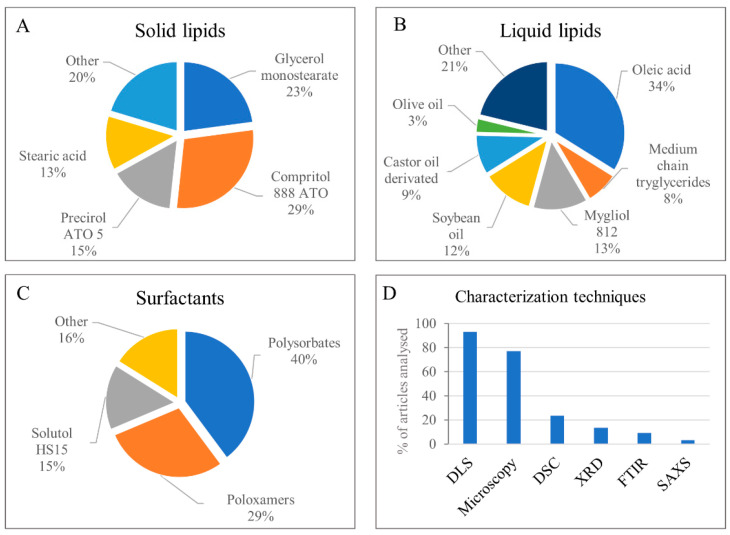
Most frequent NLC excipients: solid lipids (**A**); liquid lipids (**B**); surfactants (**C**), and techniques used for the characterisation of NLC in the reviewed articles (**D**). See Table S1 for details.

Regarding the composition, there are a wide range of lipids that can be used in the development of NLC [10], from waxes to triglycerides or fatty acids, and they can be synthetic or natural [21]. For AAs-in-NLC, the most-used solid lipids were synthetic, such as glyceryl dibehenate (Compritol^®^ 888 ATO) and glycerol monostearate, as shown in Figure 3A. As for the liquid lipids, over 54% of the articles employed natural (soybean, olive, or castor) oils or fatty (oleic) acid (Figure 3B). We found that non-ionic surfactants were mostly employed (Figure 3C), including polysorbates (40%), triblock copolymers, or poloxamers (29%), and poly-oxyethylene esters of 12-hydroxystearic acid (Solutol^®^ H-15) (15%). Surfactants and co-surfactants surround the lipid core of these nanoparticles, where functionalising agents can be added to target the DDS to the cancer cells. Interestingly, folic acid (FA) was the most frequent functionalising agent (~9% of the reviewed articles), while the phospholipid lecithin was the co-surfactant in more than 40% of the articles, helping in the stabilisation of the NLC.

High-pressure homogenisation and sonication were the most-used techniques for preparing the NLC, and various techniques were used to characterise them. In almost 100% of the analysed articles, dynamic light scattering (DLS) was the chosen technique for the determination size, polydispersity index (PDI), and zeta potential (PZ) (Figure 3D). Electron microscopy provided morphological analysis in almost 80% of articles. Structural characterisation (e.g., evaluation of crystallinity of the lipid matrix by DSC (24%) or XRD (14%)) was not so common, and only 25% of the articles reported on the stability of the nanoparticles during storage. Nanoparticle concentrations were determined in only 3% of the articles, despite their importance in determining the biological effects of the NLC [22].

The complete list of AAs-in-NLC formulations, with details on the composition, functionalisation, and main in vitro/in vivo results can be found in Table S1 (Supplementary Materials). In these studies, in vitro tests were more frequent (93%) than in vivo tests (65%) to evaluate the efficacy of AAs-in-NLC. The most important (mainly those with promissory in vivo results are described here, according to the class of the antitumour agent and focusing on the type of cancer treated and antitumour performance. Codelivery (Section 3) and NLC systems designed for non-conventional medications, such as natural compounds (curcumin) and photodynamic therapy, were also presented (Section 4).

## 2. Conventional Antineoplastics Agents Uploaded in NLC

### 2.1. Antimetabolites

Antimetabolites are compounds that interfere with normal metabolism, being structurally related to natural biological molecules. The alteration caused by antimetabolites in normal biochemical processes causes a toxic effect on the cells, which can alter their growth and division, leading to cell death [23].

#### 2.1.1. 5-Fluorouracil

The uracil analogue 5-fluorouracil (5-FU) inhibits normal function when incorporated into RNA and DNA [24]. Besides the treatment of breast, colorectal, gastric, and pancreatic cancers, it can be used to treat actinic keratosis (a precancerous lesion). It has a water solubility of 12.2 mg/mL [25] and low oral absorption, being administered intravenously [26] or topically (for actinic keratosis). Varshosaz et al. [27] prepared a 5-FU-NLC functionalised with lactobionic acid for targeting that showed 34% of encapsulation efficiency (EE). When tested in vitro in a human hepatocarcinoma (HepG(2)) lineage, encapsulation into the NLC enhanced the internalisation and doubled the cytotoxicity of 5-FU to those cells.

#### 2.1.2. Cytarabine

Cytarabine (CYT) is another pyrimidine nucleoside analogue that inhibits DNA synthesis, being commonly used to treat leukaemia. Its solubility in water (1:10) is low, but the hydrochloride form allows administration by the intravenous route [28]. Sharma et al. [29] encapsulated CYT in a small NLC (90.7 nm) and reached 49.5% EE. In vitro tests, by the MTT assay, revealed greater cytotoxicity of CYT-NLC in leukemic EL-4 cells, in comparison to free CYT, after 48 h of exposure.

#### 2.1.3. Decitabine

Decitabine (DCB) is also a nucleoside (2′-deoxycytidine) analogue that inhibits the enzyme DNA methyl transferase, causing hypomethylation of DNA. Despite its mild water solubility (23 mg/mL), its bioavailability is low (<14%) [30]. DCB is used in blood or bone marrow cancer by intravenous administration. Neupane et al. [14] developed an DCB-NLC formulation for oral application that reached 84% drug encapsulation. In ex vivo gut permeation tests, there was a four-fold increase in the permeation of DCB when encapsuled in NLC. The formulation showed greater cytotoxicity to (A549 human non-small cells) lung cancer after 24 h of treatment. The in vivo biodistribution of the nanoparticles in an Ehrlich ascites tumour-bearing mice model was performed after 4 h administration by scintigraphy imaging (using radiolabelled DCB). The activity of DCB-NLC administered orally was twice that of free DCB (administered intravenously), suggesting greater accumulation of the encapsulated drug in the tumour region, which seems promising to increase the efficacy of cancer treatment.

#### 2.1.4. Methotrexate

Methotrexate (MTX) is a folate derivative that prevents nucleotide synthesis, blocking cell replication [31,32]. It is approved by the FDA for the treatment of acute lymphoblastic leukaemia, breast cancer, head and neck cancer, lung cancer, non-Hodgkin lymphoma, and osteosarcoma. Despite being highly insoluble in water, its sodium salt has good aqueous solubility at pH > 7, enabling its application by several routes (oral, subcutaneous, intramuscular, intravenous, or intrathecal) [31].

Articles reporting the encapsulation of MTX in NLC are still in the developmental phase, with the formulation being tested only in vitro. In these studies, encapsulation of methotrexate in NLC produced formulations with EE in the range of 60 [33] to 87% [34], befitting with its high hydrophobicity. When tested in vitro in breast cancer (MDA-MB-231) cells, the magnetic MTX-NLC showed cell internalisation by caveolae-mediated endocytosis and after 72 h of exposure, were more toxic (IC_50_ = 12 μg/mL) than non-encapsulated MTX (IC_50_ = 18 μg/mL) [35]. Furthermore, control NLC (without MTX) were considered biocompatible (i.e., their excipients were non-toxic to the cells). Abdelbary and Haider [33] developed several MTX-NLC formulations employing diverse lipids and surfactants and tested them in vitro on human (prostate and ovarian) carcinoma strains. After 72 h of treatment, control NLCs of different compositions caused no cytotoxicity in the tested cancer cells. Although some MTX-NLC compositions showed significantly high cytotoxicity and sustained release, further studies are needed to correlate the in vitro results and assess their therapeutic use in vivo.

### 2.2. Antimitotics

Antimitotics describe a class of plant-derived AAs that specifically act on microtubules and can be divided into two main groups: microtubule stabilising agents (taxanes and epothilones) or microtubule destabilising agents (vinca alkaloids). Taxanes and vinca alkaloids are currently in use to treat a variety of tumours, including breast and lung cancers, neuroblastoma, rhabdomyosarcoma, acute leukaemia, and Hodgkin and non-Hodgkin lymphomas. A significant concern about antimitotic agents is their significant side effects, such as neutropenia and neurotoxicity [36].

#### 2.2.1. Taxanes

The anticancer mechanism of taxanes involves their binding to free tubulin, promoting its assembly into stable microtubules while inhibiting disassembly. The stabilisation of microtubule bundles impairs their normal function so that taxanes prevent cancer cells from dividing, leading to their death [37].

##### Cabazitaxel

Cabazitaxel (CBZ) is a semisynthetic derivative of 10-deacetylbaccatin III, an effective taxane used in the treatment of various cancers [38]. However, its clinical efficacy is limited by poor water solubility and a low safety profile. CBZ is highly toxic in patients, with a maximum tolerated dose of 25 mg/m^2^, which is significantly lower than those for other taxanes (e.g., 175 and 60–100 mg/m^2^ for PTX and DTX, respectively).

Chand et al. [38] developed CBZ-NLC aiming to decrease its toxicity in the treatment of breast cancer. In vitro studies demonstrated that, in comparison to free CBZ, there was a prolonged drug release (48 h), higher CBZ-NLC uptake (2.5 times by MDA-MB-468 and 2.1 times by MCF-7 cells) and cytotoxicity (by apoptosis) with CBZ-NLC. In addition, CBZ-NLC showed lower cytotoxicity than free CBZ to non-cancer (Beas-2B human lung epithelial) cells after 72 h of treatment.

##### Docetaxel

Docetaxel (DTX) is a semisynthetic drug produced by esterification of 10-deacetylbaccatin III, a natural compound isolated from *Taxus baccata*. It is used in the treatment of breast, lung (non-small cell), ovarian, prostate, skin, and head and neck cancers. The bioavailability of DTX is considerably better than that of paclitaxel [39], although its limited water solubility restrains its administration to a continuous intravenous delivery with formulations composed of lipophilic solvents [40].

Recent and innovative works with DTX in NLC have been reported, such as that by Zwain et al. [41] in which pegylated NLC were prepared by changing the concentrations or combining liquid lipids (Labrasol, Lauroglycol 90, Capryol and Kolliphor HS15) to enhance drug permeability through the blood–brain barrier (BBB). For the treatment of glioblastoma, the authors used 3D BBB/blood–brain tumour barrier (BBTB) in vitro models and combined optimised formulations with four liquid lipids at low concentrations to increase permeation to the central nervous system. DTX-NLC showed superior uptake by glioblastoma (strain U87MG) cells and short-term primary culture from patients (BTNW911 lineage), compared to non-cancer (SVG P12) brain cells or macrophages (RAW 264.7). The formulations effectively inhibited glioblastoma cell (U87MG and BTNW911 cells) growth, decreasing IC_50_ values and mitochondrial reserve capacities of cells, as well inducing cell arrest within 72 h of treatment, when compared to commercial DTX.

Liu and co-authors [42] developed DTX-NLC functionalised with Flk-1 (A-3), a mouse monoclonal antibody with high affinity for tumours that overexpress vascular endothelial grow factor receptor 2 (VEGFR-2). This dual-targeted (tumour and vascular targeting) functionalised DTX-NLC demonstrated superior antitumour efficacy against malignant melanoma in vitro and in vivo. The in vitro experimental results showed an effective ability to decrease the proliferation of tumour cells, and the in vivo model (Kunming female mice) showed regression of tumour growth with less toxicity when compared to non-functionalised DTX-NLC and commercial DTX (Duopafei^®^). The system was able to direct vascular delivery to the tumour, since the binding and internalisation of the nanoparticles were facilitated by the anti-VEGFR-2 antibody.

Fang et al. [43] developed nanoparticles functionalised with cysteine (Cys) aiming for the oral administration of DTX. The presence of Cys in the nanoparticles (Cys-NLC) increased their mucoadhesion due to a Cys-specific interaction with intestinal mucin. The absorption of Cys/DTX-NLC both by endocytosis and passive transport at different intestinal segments was higher than that of conventional NLC. Oral administration of Cys/DTX-NLC increased the drug’s bioavailability by 12.3 and 1.6 times in comparison to pure DTX (Taxotere^®^) and NCL, respectively.

Li and collaborators [44] developed an ultrasmall (~30 nm) NLC containing DTX and functionalised with FA (FA/DTX-NLC). The use of FA for functionalisation is due to its ability to interact, by mediated endocytosis, with folate receptors overexpressed in HeLa and other tumour cells. In vivo tests (with 4 mg/kg DTX, intravenously) confirmed the excellent targeting of FA/DTX-NLC to the tumour (due to the increased permeability and retention mediated by FA). The antitumour efficacy of the ultrasmall particles was also excellent, with superior inhibition of tumour growth (408 mm^3^), compared to that of commercial DTX (Taxotere^®^ = 658 mm^3^). Regarding biosafety, the ultrasmall FA/DTX-NLC did not cause reduction in the animals’ body weight, unlike Taxotere^®^.

Another DTX-NLC formulation, developed by Kim et al. [45], was functionalised with the RIPL peptide plus polyethyleneglycol (PEG) for selective targeting to cancer cells that overexpress hepsin. In vitro, the formulation showed great influence on ovarian cancer (SKOV3) cells, arresting the cell cycle in the G2/M phase and inducing the intrinsic apoptosis pathway related to mitochondria. The in vivo evaluation of the antitumour efficacy in a (SKOV3 ovarian cancer cells) xenograft mice model revealed the capacity of DTX-PEG-RIPL-NLC to reduce tumour growth by 2.4 and 1.6 times compared to commercial DTX and DTX-RIPL-NLC, respectively.

DTX was the third most studied AA with regard to encapsulation in NLC, and besides the articles highlighted here, Table S1 shows promising results with other studies of non-functionalised DTX-NLC systems. Overall, optimised systems were tailored to increase the targeting and specificity to tumours, thus decreasing the toxic effects of DTX to improve the quality of life of patients. It is possible to find such advantages in these works, such as increased drug bioavailability, cell uptake and apoptosis, and antitumour efficacy, as well as decreased side effects (weight loss and death in animals). The results are very promising, considering the well-known side effects of antimitotics, including neutropenia and neurotoxicity.

##### Paclitaxel

Paclitaxel (PTX), isolated from the bark of *Taxus brevifolia*, was the first antimitotic agent discovered and has several uses in cancer treatment. Poorly soluble in water (0.6 mM [46]) and used intravenously, its pharmaceutical form contains a solubilising agent that could be non-aqueous [47]. Therefore, due to these characteristics, a NLC is a promissory carrier for the delivery of PTX. Twelve articles were found (see Table S1), in which PTX was encapsulated in NLC formulations, aiming for the treatment of different types of cancer. Among those, functionalised NLC and preclinical studies are highlighted below.

Chen et al. successfully developed a PTX-NLC (6 mg/kg) formulation with nanoparticles functionalised with the glucosamine derivative stearyl-2-amino-2-deoxyglucose (2-DG) for targeted tumour delivery. The high internalisation of 2-DG by most tumour cells is due to their high energy consumption for cell proliferation. The authors evaluated the weight and volume of tumour-bearing mice (induced by inoculation of MCF-7 human breast tumour cells). In the animals treated intravenously with 2-DG/PTX-NLC, the tumour growth rate and systemic toxicity were reduced and survival time was longer when compared to animals treated with commercial PTX [48].

Yang et al. [49] synthesised PTX-NLC (3 mg/kg) functionalised with hyaluronic acid (HA) aiming at its binding to CD44, a cell surface marker overexpressed in tumours. Besides in vitro cytotoxicity, the authors measured the antitumour efficacy in vivo (in melanoma murine tumours induced with B16 cells), pharmacokinetics and tissue biodistribution. HA/PTX-NLC showed greater cytotoxicity than commercial PTX. In vivo, a significant decreased tumour volume was measured after intravenous administration of HA/PTX-NLC, which also prolonged the circulation time of PTX in the blood and promoted its specific accumulation in the tumour area.

To manage the drug resistance (DR) of lung cancer cells, Kaur and colleagues [50] developed a PTX-NLC optimised by design of experiments (DoE). The formulation demonstrated 72 h sustained release and elevated uptake by Caco-2 human intestinal cells in vitro. PTX-NLC inhibited the efflux of p-glycoprotein (P-gp) from Caco-2 cells, increasing drug cellular uptake, which was probably due to the small particle sizes (<180 nm) and high surfactant concentrations. In vivo distribution studies confirmed the therapeutic efficacy of the developed formulation, specifically in healthy rat lungs. The inhalable spray-dried formulation was considered adequate for the release of PTX in the airways. The inhalable PTX-NLC spray-dried system is promising for the treatment of DR in lung cancer.

For the treatment of brain cancer, Emami and co-workers [51] developed and optimised (by DoE) a formulation functionalised with transferrin (Tf/PTX-NLC) to favour crossing the BBB. Transferrin receptors are overexpressed in certain brain endothelial cells. The system was tested in vitro over U-87 malignant glioma cells, and as expected, Tf/PTX-NLC promoted increased cytotoxicity (~40% of cell survival at 0.586 µM) when compared to the free drug, commercial PTX (Anzatax^®^), and non-functionalised NLC-PTX after 48 h of incubation. The favourable outcomes were attributed to the faster cellular uptake of the functionalised formulation and proposed as an alternative for the treatment of brain cancer (e.g., glioma) that has a low survival time and fast tumour spread throughout normal brain tissue, making its complete surgical removal impossible.

Aiming for the treatment of lung tumours, Sun et al. [52] functionalised a PTX-NLC formulation with the poly-arginine (R8) cell-penetrating peptide and performed a systematic in vitro investigation of its effect on A549 (human alveolar basal epithelial adenocarcinoma) cells. The cytotoxicity of R8/PTX-NLC was higher than that of the non-functionalised PTX-NLC system. The study demonstrated that, because of the R8 molecules on the surface of the nanoparticles, the uptake of the functionalised formulation was four times greater than that of PTX-NLC by the tumour cells.

Ucar and co-authors [53] synthesised a NLC radiolabelled with 99 mTc(CO)3^+^ and functionalised with FA for the delivery of PTX, aiming at improving drug release and making the nanoparticle a contrast agent. In vitro (MCF-7, A549 and HeLa cells) and in vivo (biodistribution and intravenous injection) studies indicated that the developed system may be useful as a theragnostic agent for tumour tissues overexpressing folate receptors. PTX was used as a model drug without proper focus in its anticancer activity.

In 2019, Bang and collaborators [54] developed PTX-NLC functionalised with platelet membrane proteins (PlP) and tested it in vitro and in vivo (biodistribution and intravenously). The idea was to increase drug bioavailability, since platelets take part in angiogenesis and interact with circulating cancer cells. In vitro assays confirmed the affinity and high cytotoxicity (IC_50_ = 10 µg/mL) of PlP/PTX-NLC in SKOV3 ovarian cancer cells. In a biodistribution study, the functionalised formulation was captured by the RES and accumulated in the liver (which is responsible for platelet metabolization), showing the success of the functionalisation.

According to the literature, PTX is the second most studied AA in terms of encapsulation in NLC. Functionalised PTX-NLC is more frequent than non-functionalised nanoparticles (Table S1) but enhanced antitumour effects and lower toxic effects are observed in both cases. The results point out NLC as promissory PTX carriers in different types of tumours (e.g., ovarian, colorectal, hepatocellular, and breast cancers), suggesting its application in clinical studies.

#### 2.2.2. Vinca Alkaloids (Vincristine)

This group of AAs involves the alkaloids vinflunine, vinblastine, vincristine, vinorelbine, vindesine, and eribulin, which are extracted from *Catharanthus roseus*. These alkaloids bind to β-tubulin near the guanosine triphosphate binding site (the vinca domain) at the interface of the β-α-tubulin heterodimer. Such binding prevents the straightening of curved tubulins, interfering with the growth and assembly of microtubules [36].

Just one study on the encapsulation of vinca alkaloids in NLC has been published so far, with vincristine sulphate (VCR). VCR, despite its neurotoxicity, is widely used for treating different cancers, such as malignant lymphoma, breast cancer, and acute leukaemia. Moreover, low oral bioavailability and fast elimination hamper the application of VCR by the oral route, and only the injection formulation is used in clinics [16]. Gao and collaborators [16] developed a cationic NLC functionalised with HA (HA/VCR-NLC) for the oral delivery of VCR. As explained before, HA specifically binds to the CD44 cell surface marker that is overexpressed in some tumours. In vitro HA/VCR-NLC showed higher uptake and greater cytotoxicity, inducing apoptosis of breast cancer (MCF-7) cells greater than free VCR. In vivo pharmacokinetic studies showed the improved oral bioavailability (two times) of HA/VCR-NLC, indicating the effectiveness of the functionalised nanoparticles for delivering VCR by the oral route.

### 2.3. Alkylating Agents

Alkylating agents are pioneering AAs and have been used since the 1940s, with an ever-increasing therapeutic repertoire. According to their chemical structure, alkylating agents are classified as triazines, nitrogen mustards, nitrosoureas, ethylenimines, alkyl sulfonates, and platinum derivatives [55,56]. They act directly in the DNA molecule, affecting the cell replication process, which causes a delay or inhibition of tumour growth [57,58]. However, systemic toxicity (action on normal cells) and intrinsic DR are their major limitations [58,59].

#### 2.3.1. Cisplatin

The platinum derivative cisplatin (CIS) is poorly soluble in water (2.5 mg/mL). It is used to treat several (bladder, testicular, and ovarian) cancers, mostly associated with other drugs, and by an intravenous route. Zhang et al. [60] developed and evaluated NLC uploaded with CIS and functionalised with FA (FA/CIS-NLC) for cervical cancer chemotherapy. FA/CIS-NLC showed higher cytotoxicity (three times lower IC_50_) to HeLa cells when compared to that in free CIS. In vivo antitumour efficiency, evaluated in a mice-bearing human cervical cancer xenograft, revealed superior tumour regression in animals treated with FA/CIS-NLC (79.3%) in relation to CIS-NLC (64.4%) and free CIS (18.8%), without significant body weight loss.

#### 2.3.2. Dacarbazine

Dacarbazine (DZ) is a triazine used to treat metastatic melanoma and Hodgkin lymphoma. It has low water solubility [61], being administered by the intravenous route. Thus far, there is only one report on the encapsulation of DZ in NLC, by Almoussalam and Zhu [62]. The authors developed a NLC-DZ formulation with high (98%) EE and sustained release (>30 h) that was proposed for topical application (against cutaneous melanoma or epidermoid carcinoma) but failed to demonstrate its effectiveness against cancer cells in vitro or in vivo.

#### 2.3.3. Ifosfamide

Ifosfamide (IFOS) is a nitrogen mustard that inhibits DNA synthesis, inducing cell death. IFOS is used intravenously in the treatment of testicular cancer. Velmurugan and co-workers developed NLC formulations for the encapsulation of IFOS [18,63,64] and tested them in vivo [18,61]. Firstly, the authors determined IFOS-NLC pharmacokinetics and antitumour efficiency after oral administration to Swiss mice in a Dalton’s ascitic lymphoma model [18]. The better antitumour activity of IFOS-NLC was demonstrated by the increase (35%) in animal survival in relation to an IFOS suspension (administered intraperitoneally) and by improvement of the biochemical parameters of the animals. In another paper [64], the authors administered the IFOS-NLC formulation orally to Wistar rats and registered increased plasma concentrations and slower IFOS release, resulting in an improvement in bioavailability of the drug in relation to the administration of an IFOS suspension. Besides, the group demonstrated a reduction in toxicity with IFOS encapsulated in NLC formulations.

#### 2.3.4. Mechlorethamine

Mechlorethamine (MCT) is another nitrogen mustard alkylating agent that is soluble in its salt (hydrochloride) form. It is employed in the treatment of Hodgkin lymphoma. MCT salt could be administered by the topical, intrapleural, intraperitoneal, and intrapericardial routes. Gidwani et al. [17] developed a MCT-NLC formulation optimised by DoE with 93% EE that was stable for 180 days and tested it in vitro and in vivo. Cytotoxicity assays revealed that MCT-NLC was more toxic to K562 (human immortalised myeloid leukaemia) cells than the free drug. In vivo, after oral administration to normal Wistar rats, MCT-NLC improved the absorption, half-life, and bioavailability of MCT in relation to free MCT.

#### 2.3.5. Temozolomide

Temozolomide (TMZ) is a triazine of low water solubility used for the treatment of glioblastoma [65], either orally or intravenously. Several groups developed TMZ in NLC formulations. Khan et al. [66] investigated the efficacy of TMZ-loaded nanoparticles (TMZ-NLC) to enhance brain targeting via the nasal route. The formulation optimised by DoE showed a high (81.7%) EE. In ex vivo experiments with porcine nasal mucosa, the formulation almost doubled the flux and permeability coefficient, in comparation to non-encapsulated TMZ. Pharmacokinetic studies, after nasal administration to mice, revealed higher concentrations of TMZ-NLC in the brain (457%) in relation to a TMZ suspension, confirming the improved brain delivery and increased nasal permeation of the formulation.

Qu et al. [67] developed NLC, SLN and polymeric nanoparticles loading TMZ for the treatment of glioblastoma. Among the three kinds of nanoparticles, TMZ-NLC showed the best antitumour activity, both in vitro and in vivo. When tested in solid tumour-bearing mice (induced with U87 MG cells), the NLC formulation promoted 85% regression in tumour volume after intravenous administration for 21 days, against 59% for TMS-SLN and 45% for the polymeric nanoparticles, in comparison to the free TMZ.

Chen et al. [68] developed and evaluated NLC formulations with TMZ for the treatment of gliomatosis cerebri, a rare glioma. Cytotoxicity assays in U87 MG glioma cells showed lower (four-fold) IC_50_ values for TMZ-NLC when compared to free TMZ. The authors co-encapsulated the plasmid of the enhanced green fluorescent protein (EGFP) in the nanoparticles, allowing the detection of EGFP-positive cells by cytometry. The expression of EGFP was successful and did not interfere with the in vivo antitumour efficacy of the formulation evaluated in mice bearing malignant glioma, which revealed a reduction (3.3 times) in tumour size after treatment with TMZ-NLC in comparison with free TMZ.

Song and collaborators [69] developed a TMZ-NLC functionalised with the arginine-glycine-aspartic acid (RGD) peptide and conjugated in PEG-b-distearoylphosphatidylethanolamine (PEG-DSPE) for the treatment of glioblastoma multiforme. The RGD peptide binds to integrin receptors, helping in cell recognition (targeting) and attachment. In vitro cytotoxicity tests, using the MTT assay, showed that RDG/TMZ-NLC improved the cytotoxicity against glioblastoma (U87 MG) cells with IC_50_ values two- and ten-fold lower than TMZ-NLC and free TMZ, respectively. The in vivo effect, evaluated in solid tumour-bearing mice (induced with U87 MG cells) after intravenous injection, revealed 83% tumour volume regression with RDG/TMZ-NLC against 66.3 and 20.8% regression promoted by TMZ-NLC and free TMZ, confirming the high therapeutic efficacy of the RDG/TMZ-NLC formulation. In summary, in the articles analysed, TMZ encapsulation into NLC improved the cytotoxicity and tumour regression and promoted targeted delivery of TMZ, which are fundamental strategies for achieving brain cancer remission.

### 2.4. Antitumour Antibiotics

Antitumour antibiotics are among the most important AAs classes, and the compounds are derived from natural sources [70]. Among the several groups of drugs of this class, three have been contemplated with NLC formulation studies: anthracyclines, podophyllotoxins and camptothecins.

#### 2.4.1. Anthracyclines

Anthracyclines are an important class of antitumour antibiotics used since the 1960s, which include doxorubicin, daunorubicin, epirubicin, and idarubicin. Anthracyclines bind to DNA to induce several cellular events that lead to cell death. They are administered intravenously to avoid degradation by the digestive tract [71].

We have found ~20 articles in which anthracyclines have been incorporated into NLCs. Most of them studied doxorubicin, alone or in codelivery with other drugs, and just one reported the incorporation of another anthracycline, pirarubicin (4′-O-tetrahydropyranyl-adriamycin). The antiproliferative effects of the NLC formulations were investigated against different (breast, lung, prostate, melanoma, and colon) cancer cells.

##### Doxorubicin

Doxorubicin (DOX), isolated from the *Streptomyces peucetius* var. *caesius* is used in the treatment of several (bone sarcoma, breast, ovary, bladder, and thyroid) cancers. DOX hydrochloride salt is readily soluble in water [72], and it is marketed as powder or solution for intravenous injection, but cardiac toxicity is an undesirable side effect that affects ~11% of patients treated with DOX [73].

In 2014, Mussi et al. [74] formulated a DOX-containing NLC and tested it in vitro against the NCI/ADR-RES multidrug-resistant breast cancer cell line, finding higher anticancer activity than free DOX. In the following year, those authors [75] modified the NLC formulation with the use of layer-by-layer assembly of polyelectrolytes and coated them with PEG (PEG/DOX-LbL–NLC). In vivo tests in an orthotopic murine mammary carcinoma model (injected with 4T1 cells) revealed an increased half-life in blood, lower liver accumulation, increased amounts of DOX in tumour cells, and lower tumour volume after treatment with PEG/DOX-LbL–NLC in comparison to naïve or free DOX groups.

Fernandes et al. [76] compared the antitumour activity of DOX encapsulated in NLC formulations and liposomes in breast tumour-bearing mice (induced with 4T1 cells). NLC-DOX was the most efficient treatment, decreasing tumour growth in 74% in comparation to liposomal-DOX (68%) and free DOX (50%). Furthermore, these authors explored the use of DOX-NLC prepared with ω-3 docosahexaenoic acid (DHA) and radiolabelled with Tc-99m (NLC-DHA-DOX) [77]. The nanoparticles were injected in mice with the same (4T1 cells) mammary carcinoma model. Not only was the formulation able to reduce the systemic toxicity in comparison to free DOX but also the incorporation of Tc-99m allowed determination of the biodistribution profile and tumour accumulation. In 2018, the same group [78] introduced α-tocopherol succinate (TS) in the DOX-NLC formulation, as an additive to improve its antitumour properties. The TS/DOX-NLC displayed improved antitumour activity (~80%) in relation to free DOX or DOX-NLC without tocopherol (~50%) in the breast carcinoma-bearing mice.

Zhang et al. [19] developed pegylated DOX-NLC with soya lecithin and PEG-DSPE. Cytotoxicity tests in PC3 (human bone metastasis of grade IV prostate cancer) cells showed a decrease in IC_50_ (41 nM with DOX-NLC) regarding DOX in suspension (210 nM). Pharmacokinetic studies in male Sprague-Dawley rats after oral administration revealed that DOX-NLC improved in the bioavailability two- to three-fold in relation to free DOX.

In 2019, Li et al. [79] proposed a peculiar pH-sensitive NLC based on the composition of lipoproteins, with ApoB-100 protein, oleic acid and DOX, aiming at enhancing tumour-targeted therapeutic efficiency with this low density lipoprotein (LDL)-mimetic system. In vitro results revealed that ApoB/DOX-NLC was successfully phagocytosed by the LDL receptor, increasing DOX cytotoxicity to 4T1 cells. In vivo, in an orthotopic breast cancer model (induced with 4T1 cells), biodistribution showed that ApoB/DOX-NLC nanoparticles, different than free DOX, promoted drug accumulation in the tumour without any significant deposition in the heart. As for the antitumour activity, in comparison to free DOX, ApoB/DOX-NLC promoted lower tumour volume, decreased mortality without body loss (in relation to naïve animals), and no cardiac tissue damage after ex vivo analysis.

Lages et al. [80] loaded DOX in nanoparticles that contained TS and DHA (DHA-TS/DOX-NLC) to enhance the efficacy of the formulation in the treatment of breast cancer. In vitro, in 4T1 breast cancer cells, the formulation effectively promoted cell death. In vivo, in tumour-bearing BALB/c mice (injected with 4T1 cells), DHA-TS/DOX-NLC showed efficacy when intravenously applied, reducing tumour growth (76.6%) better than DOX-NLC (66.5%) and free DOX (64.6%) in relation to the naïve group. Moreover, the formulation reduced mortality and heart/liver toxicity, as well as prevented lung metastasis.

Han et al. [81] encapsulated DOX in NLC functionalised with transferrin to target the particles for lung tumour cells. The authors also co-encapsulated the EGFP plasmid in the nanoparticles to follow the fluorescence of the expressed protein inside the tumour. The antitumour activity of the formulations against (A549-induced) alveolar adenocarcinoma in mice was determined, and the presence of transferrin significantly enhanced the transfection efficiency, promoting major tumour inhibition efficiency (66%) in relation to free DOX (18%).

**Figure 4:**
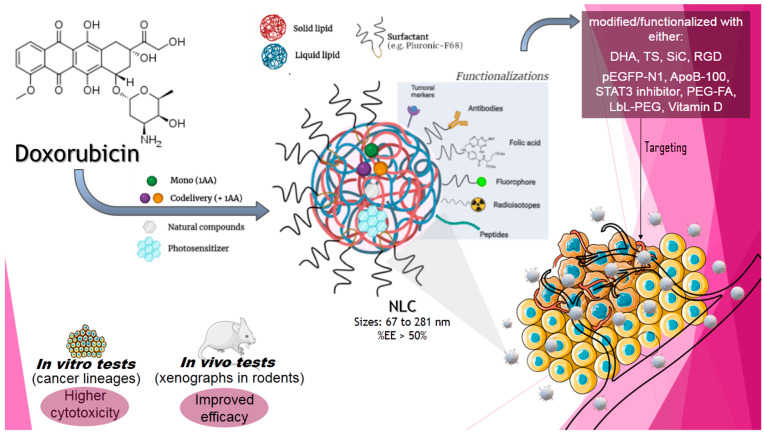
Main research and results obtained by the encapsulation of doxorubicin in nanostructured lipid carriers (abbreviations as in Table S1).

Since DOX was by far the most studied AA regarding encapsulation in NLC, either alone or in codelivery (see Section 3), Figure 4 depicts the main results obtained with DOX-in-NLC. These NLC, although developed with different excipients, presented high EE (>50%) and were tested in vivo in several cancer models (breast, prostate, skin, and colon) by intravenous and oral routes of administration. Encapsulation in NLC inhibited tumour growth in all reported cases, with reduction in mortality and metastasis. These results are likely due to the improvement of the pharmacological properties of DOX (increased bioavailability and high in situ concentration) when encapsulated. DOX tumour-targeting by the NLC was also accompanied by a decrease in systemic toxicity, the most undesirable side effect of this antitumour agent, confirming DOX-in-NLC as a very interesting DDS for future clinical application.

##### Pirarubicin

Pirarubicin (THP) is a DOX analogue that is a first-line treatment for breast cancer, but its low molecular weight facilitates non-specific distribution to healthy cells, with undesirable side effects. Deng et al. [82] encapsulated THP in NLC for the treatment of breast cancer. The formulation significantly reduced THP cytotoxicity to DC2.4 mouse dendritic cells, while improving the cytotoxicity in 4T1 cells (resistant breast cancer cell line). In a BaLB/c mouse-bearing (4T1 breast cancer) model, when intravenously co-injected with iRGD, a tumour-penetrating peptide, THP accumulation in the tumour was elevated, leading to increased expression of IFN-γ and INF-α cytokines, lower tumour growth rates (74.2% compared to the saline group), and cell death. A significant reduction in the proliferation of bone marrow cells was also observed, confirming the specificity (targeting) to tumour cells.

#### 2.4.2. Irinotecan

Irinotecan (Ir) is a semisynthetic analogue of camptothecin, an antitumour antibiotic extracted from the *Camptotheca acuminata* tree [70]. Ir is used in the treatment of metastatic colon or rectum cancer [83], since its hydrochloride salt has enough aqueous solubility for intravenous administration. Negi and co-workers [84,85] developed a formulation of Ir-NLC functionalised with HA (HA/Ir-NLC). The formulation showed increased cytotoxicity (9.5 times) against Colo-320 cells when compared with that of non-functionalised nanoparticles (Ir-NLC, 5.3 times) and in comparison to free Ir [84]. HA/Ir-NLC was also less haemolytic (1%) than Ir-NLC (11%). In vivo, in an Ehrlich’s ascites tumour mice model, intravenously administered HA/Ir-NLC caused a tumour reduction 5.8 times higher than that of free Ir and 2.6 times higher than that of non-functionalised Ir-NLC. Finally, the haematological parameters (neutropenia, thrombocytopenia, and mild anaemia) of HA/Ir-NLC were similar to that of free Ir [85].

#### 2.4.3. Etoposide

Etoposide (ETP) is a semisynthetic derivative of podophyllotoxin, a potent natural antitumour agent. ETP is poorly soluble in water and it can be administered by the oral or intravenous route for the treatment of several (gastric, lung, testicular, and blood) cancers [86]. In 2011, Zhang et al. [86] developed an ETP-NLC formulation, aiming for oral delivery. For that, they functionalised the formulation using PEG-DSPE. The PEG/ETP-NLC showed high intestinal absorption in ex vivo experiments. In vivo pharmacokinetic studies in male Sprague-Dawley rats showed a 3.5-fold increase in ETP bioavailability with the PEG/ETP-NLC formulation in relation to the free drug.

Jiang et al. [87] loaded ETP in NLC, aiming for the treatment of gastric cancer. The ETP-NLC was more cytotoxic to SGC7901 human gastric cancer cells (IC_50_ = 6.3 μg/mL) than free ETP (IC_50_ = 56.5 μg/mL). In a gastric tumour-bearing BALB/c nude mice model, after intravenous administration, ETP-NLC promoted a two-fold higher tumour inhibition rate when compared to free ETP.

Zhang and colleagues [88] developed ETP-NLC functionalised with FA (FA/ETP-NLC) and tested it in a gastric tumour-bearing BALB/c nude-mice model. After intravenous administration of FA/ETP-NLC, the ETP concentration in the tumour was higher than that in other tissues, while it was mainly distributed in the heart and kidney after injection of free ETP. After 21 days of treatment, tumour volume was 219 mm^3^ in the FA/ETP-NLC group, against 416 mm^3^ in the ETP-NLC group and 1130 mm^3^ in animals treated with free ETP. Confirming the lower systemic toxicity, animals that received the NLC formulations did not show changes in body weight, food intake, and inactive moving behaviour, which was opposite to naïve animals or those treated with free ETP.

## 3. Codelivery of Conventional AAs by Nanostructured Lipid Nanoparticles

Codelivery nanosystems (DDS uploading at least two agents) for the treatment of cancer have seen heavy growth in the last few years [89]. These systems promote synergistic and potentialized inhibition of tumour growth in comparison to the effect of each individual active, free, or encapsulated [90]. Since NLC is a promissory system for the encapsulation of hydrophobic molecules, it is also a suitable carrier for the simultaneous encapsulation of two or more AAs. Therefore, combined chemotherapy using NLC as carriers brings advantages, such as drug dose reduction, multitargeted therapy, synergistic action, and defeat of multi-DR [91]. The most promising reports with this codelivery approach are described below.

### 3.1. DOX Codelivery with NLC Formulations

#### 3.1.1. DOX and PTX

Wang et al. [92] developed a formulation containing DOX and PTX in a cationic NLC composed of Compritol^®^ 888 ATO, oleic acid, soya lecithin and dioleyl oxypropyl trimethyl ammonium chloride (DOTMA) for the treatment of lung cancer. The nanoparticles showed high EE (84 and 82% for DOX and PTX, respectively) and promoted sustained release for up to 48 h. The cytotoxic effect of PTX/DOX-NLC on NCL-H460 non-small lung cancer cells was significantly higher than that of each drug, either free (nine-fold) or encapsulated in NLC formulations (three-fold). In vivo investigation in mice grafted with (NCL-H460) lung cancer cells revealed high targeting capacity and antitumoural activity for the codelivery formulation (PTX/DOX-NLC), which exhibited the highest tumour volume inhibition rate (84%), followed by DOX-NLC (65%), PTX-NLC (64%) and free DOX or PTX (~26% each). The superior antitumour effect was assigned to the synergistic action of the AAs in the formulation, which also showed lower systemic side effects.

Chang et al. tested the PTX/DOX-NLC formulation [93] against glioma stem cells. In vitro studies revealed that the nanoparticles inhibited cell proliferation by suppression of the PI3K/AKT/mTOR signalling pathway, promoting apoptosis. Tumour volume regression in vivo was confirmed in a glioma mice xenograft model (induced with U87 cells), where treatment with PTX/DOX-NLC was more efficient (~77%) than DOX or PTX, free (~33%) or encapsulated in NLC formulations (~63%).

Another NLC formulation with DOX and PTX for the treatment of lung cancer was developed by Kaur et al. [20], which tested different surfactants and selected the formulation composed of soya lecithin, oleic acid, and Cremophor EL. In vitro, the formulation promoted sustained drug release (>20 days) and improved cell uptake and transfection efficacy in lung adenocarcinoma (A549) cells. In vivo (pharmacokinetic and lung deposition studies), the formulation enhanced retention and drug accumulation in the lungs of healthy Wistar rats without causing tissue abnormality, thereby helping to reduce the toxic effects in non-target tissues.

#### 3.1.2. DOX and CIS

Di et al. [94] developed a formulation of nanoparticles for the codelivery of DOX and CIS (DOX/CIS-NLC), aiming for the treatment of breast cancer. The characterisation showed an EE of 90 and 87% for DOX and CIS, respectively. In the cytotoxicity assays with MCF-7 and MCF-7/ADR cells, DOX/CIS-NLC exhibited the highest antitumour activity (IC_50_ = 18.6 and 9.3 μM for DOX and CIS, respectively) compared to NLC containing only DOX (86.1 μM) or CIS (13.2 μM) and free drugs (353.6 and 92.7 μM for DOX and CIS, respectively). Tissue distribution and in vivo antitumour efficacy were evaluated in a MCF-7/ADR tumour-bearing BALB/c nude mice model. Free DOX was mostly concentrated in the heart and kidneys, while DOX/CIS-NLC showed higher concentrations in the tumour tissue for up to 48 h after subcutaneous inoculation. The inhibition of tumour growth was more efficient in the DOX/CIS-NLC group (72%), showing synergistic activity and lower systemic toxicity than with NLC formulations containing only DOX or CIS (19 and 38%, respectively) in comparison to a DOX-CIS suspension. Animals treated with the NLC formulations showed no significant body weight loss, as observed in the free DOX-CIS or saline-treated groups.

#### 3.1.3. DOX and VCR

DOX and VCR are commonly used AAs and have limited clinical use due to systemic toxicity. Dong et al. evaluated the codelivery of DOX and VCR by NLC formulations to treat lymphoma [95]. The cytotoxicity of DOX/VCR-NLC at different w/w ratios (5/1, 2/1, 1/1, 1/2, and 1/5) revealed that DOX-VCR equivalent doses had the lowest IC_50_ (0.26 µg/mL) against LY1 cells when compared to those of free DOX (7.51 µg/mL), free VCR (9.82 µg/mL), or NLC formulations containing only DOX or VCR (2.63 µg/mL and 3.31 µg/mL, respectively). The same cells were inoculated into the armpit of BALB/c mice for in vivo evaluation of antitumour efficacy. The most obvious tumour regression (80%) was observed in the codelivery NLC group, revealing a synergistic effect between the two AAs.

#### 3.1.4. DOX, GEM and VCR

Ni et al. [96] encapsulated, in the same NLC formulation, three drugs commonly used in the treatment of lymphoma. Firstly, the authors synthesised a DOX-GEM prodrug that was then mixed with VCR and other excipients to prepare the NLC. The DOX/GEM/VCR-NLC showed high encapsulation (>86% for the three AAs) and cytotoxicity against Raji lymphoblast-like cells. In vivo, after intravenous administration to lymph cancer-bearing mice (injected with Raji cells), the group treated with DOX/GEM/VCR-NLC showed high levels of drug accumulation in the tumour, liver, spleen, and lungs, but lower levels in the heart. Accordingly, the antitumour activity was more evident in the formulation (86% tumour weight reduction) in relation to free drugs (<40%) without differences in body weight lost, food intake, and energy sag in relation to the naïve group. Pharmacokinetic studies revealed a sustained drug release (48 h), with low systemic toxicity when the drugs were encapsulated in the codelivery system, showing its potential for the treatment of lymphoma.

#### 3.1.5. DOX and Baicalein

Liu et al. [97] developed a NLC functionalised with HA for breast-cancer targeting, aiming for the codelivery of DOX and baicalein (BCL), a natural bioactive flavonoid with antitumoural effects. The nanoparticles were tested on MCF-7/ADR cancer cells and in a murine cancer model. In both cases, HA-BCL/DOX-NLC showed better antitumour activities. In a culture of MCF-7/ADR cells, a solution of BCL-DOX presented synergistic effects, but the cytotoxicity HA-BCL/DOX-NLC was even higher (12-fold) than that. In vivo tests were conducted in a model of mice bearing human breast cancer after intravenous administration of the formulations. The HA-BCL/DOX-NLC formulation was the most effective at reducing (89%) tumour volume when compared to the control group. Interestingly, the BCL/DOX-NLC and BCL-NLC (without HA functionalisation) groups, showed good results, reducing the tumour volume in relation to control group in 82 and 72%, respectively.

#### 3.1.6. DOX and Sclareol

In 2019, Borges et al. [98] explored the codelivery of DOX and sclareol (SC), a natural diterpene, by NLCs, aiming for the treatment of breast cancer. The DOX/SC-NLC formulation was tested in vitro in MDA-MB-231 and 4T1 breast cancer cells. The synergistic effect between DOX and SC was observed both in free solution (DOX-SC) and when encapsulated in NLC formulations. In the same way, in a tumour-bearing female BALB/c mice model (induced with 4T1 cells), superior tumour inhibition was determined with the drug association (66.7%, free DOX-SC; 53.2%, DOX/SC-NLC) than with free DOX (10.4%) or NLC-DOX (15.7%) relative to the control (naïve) group. Despite this, DOX/SC-NLC promoted lower body weight loss and was less myelosuppressive than free DOX-SC injection, indicating decreased systemic toxicity for DOX when loaded in the DOX/SC-NLC nanoparticles.

#### 3.1.7. DOX and β-Elemene

Cao et al. [99] developed a pH-sensitive NLC (doped with acid sensitive hydrazone (Hyd)) for the codelivery of DOX and β-elemene (ELE), a natural sesquiterpene, aiming for the treatment of lung cancer. When tested against mice tumour xenografts (produced with subcutaneously injected A549/ADR cells), the DOX/ELE-Hyd-NLC formulation deeply inhibited tumour growth (82.9%) in comparison to non-pH-sensitive nanoparticles (DOX/ELE-NLC, 64.5%), DOX-Hyd-NLC (61.9%), ELE-Hyd-NLC (46.7%) and free DOX-ELE (24.1%) after intravenous administration.

#### 3.1.8. DOX and β-Lapachone

To overcome multi-DR in breast cancer cells, Li et al. [100] developed a pegylated (PEG-oleic acid) NLC for the loading of DOX and β-lapachone (LAPA), a natural quinone with anticancer properties. The formulation was tested in vitro, promoting higher retention of DOX in MCF-7/ADR cells. Tests in a tumour-bearing nude mice model (infected with MCF-7/ADR cells) confirmed the enhanced uptake of DOX, which was promoted by reactive oxygen species (ROS) produced by LAPA, in the DOX/LAPA-NLC formulation. Additionally, its higher anticancer efficiency (lower tumour volume) in relation to monodelivery (DOX-NLC and LAPA-NLC) nanoformulations indicates that DOX/LAPA-NLC is a suitable platform to overcome multi-DR in breast cancer cells.

### 3.2. PTX Codelivery by NLC Formulations

#### 3.2.1. PTX and CIS

Yang and co-workers [101] developed a NLC functionalised with FA for the simultaneous delivery of PTX and CIS, which is the best choice for the treatment of head and neck cancers. The codelivery system presented high EE (79 and 82% for PTX and CIS, respectively). The in vivo drug distribution of FA-CIS/PTX-NLC revealed lower concentrations of PTX and CIS in the heart and kidneys, which was associated with a decrease in systemic toxicity. The drug concentrations in the tumour tissue remained high until 48 h after injection, revealing drug-sustained release by the FA-CIS/PTX-NLC formulation. The in vivo antitumour efficiency was evaluated in a squamous carcinoma (FaDu cells)-bearing head and neck cancer mice model, with significant tumour regressions (81.1%) compared to the formulation without FA functionalisation (70.4%), free drugs (11.0 and 9.3% for PTX and CIS, respectively) and NLC containing only PTX or CIS (48.8 and 41.7%, respectively). No significant weight loss was observed compared to the free drug solutions.

#### 3.2.2. PTX and Demethylnobitelin

Guo and collaborators [102] prepared a NLC functionalised with the monoclonal antibody cetuximab (which binds to the epidermal growth factor receptor overexpressed in different tumour cells) to deliver PTX and 5-demethylnobiletin (DMN) as a combined therapy for lung cancer. DMN is a flavonoid from citrus fruits with metabolites that effectively inhibit colon [103] and other cancers. Higher cell uptake was determined with the targeted NLC (65.8%) than without the antibody (35.5%) by A549/PTX lung cancer cells. The combined treatment synergistically decreased the viability of cells in comparison to PTX-NLC or DMN-NLC. When intravenously administered in lung tumour xenografts (induced with A549/PTX cells) in mice, a stupendous reduction in tumour volume (from 1010.2 to 211.2 mm^3^) was observed.

### 3.3. CIS Codelivery by NLC Formulations

CIS and 5-FU

Beyond the reports of DOX/CIS or PTX/CIS NLC formulations (described above), Qu et al. [104] prepared a NLC for the codelivery of CIS and 5-FU, the drugs of choice for gastric cancer therapy. At first, a prodrug (FU-stearic acid conjugate) was synthesised and after it was mixed with CIS and incorporated into the NLC functionalised with HA (HA-CIS/FU-NLC) as a strategy for selectively targeting gastric cancer cells. In mice bearing BGC823 human gastric cancer xenografts, HA-CIS/FU-NLC promoted major tumour reduction (~80%). The authors also reported a decrease in systemic toxicity in the HA-CIS/FU-NLC group, as seen by the absence of body weight loss, showing that codelivery of the drugs and NLC functionalisation successfully improved the antitumour activity of both AAs.

### 3.4. TMZ Codelivery by NLC Formulations

#### TMZ and VCR

Wu et al. [105] performed a comparative study between SLN and NLC formulations for the codelivery of TMZ and VCR, aiming at treating glioma. The TMZ/VCR-NLC showed a higher EE (TMZ = 89%, VCR = 85%) than SLN and prolonged drug release in vitro. The cytotoxicity of both formulations was tested in U87 MG glioma cells, and the IC_50_ value of TMZ/VCR-NLC (0.23 µM) was five times lower than that of TMZ/VCR-SLN (1.18 µM). The antitumour efficiency, evaluated in a BALB/c nude mice solid tumour (U87 MG cells) model, was higher (>80% tumour regression) in the TMZ/VCR-NLC group when compared with those for TMZ/VCR-SLN (56%), free TMZ (26%) or VCR (~15%), showing the synergistic effect of both AAs when encapsulated in NLC formulations.

## 4. New Approaches in Chemotherapy

### 4.1. Curcumin as a Non-Conventional AA Encapsulated in NLC Formulations

Curcumin (CUR) or diferuloylmethane is a polyphenol extracted from the rhizome of *Curcuma* spp. CUR has been used as a food additive for a long time, but its antitumour effects were demonstrated in preclinical and clinical studies [106]. The cytotoxicity of CUR toward tumour cells is related to angiogenic effects, induction of apoptosis, and interference in the proliferation cell cycle [107]. The water solubility of CUR is very poor, which makes it suitable for encapsulation in NLC formulations.

Thus far, there are six reports in the literature describing the effectiveness of NLC encapsulation to increase the in vitro cytotoxicity of CUR against cancer cells (see Table S1), and just one report by Chen et al. [108] that, beyond registering a 90% increase in cytotoxicity of CUR-NLC in A172 human brain cancer cells (by apoptosis induction and increased ROS levels), has also demonstrated its in vivo effects. Indeed, when CUR-NLC was intraperitoneally administered in a mice-bearing human lung cancer xenograft model, it increased drug bioavailability and reduced tumour volume (by 82% in comparison to naïve animals).

Interestingly, most of the CUR-in-NLC studies propose its co-encapsulation with another conventional AA to promote synergistic anticancer effects. A NLC formulation for the codelivery of CUR and imatinib (IMT, a tyrosine kinase inhibitor) was proposed by Varshosaz et al. [109] for the treatment of non-Hodgkin lymphoma. Aiming for a better target for CD20 receptors in lymphoma cells, the NLC was functionalised with rituximab (R, an anti-CD20 receptor antibody). The IC_50_ for the mixture of IMT and CUR was 1.9 µg/mL, while R-IMT/CUR-NLC decreased the IC_50_ to 1.3 µg/mL. The non-functionalised IMT/CUR-NLC formulation showed an IC_50_ of 2.0 µg/mL, confirming the better cytotoxic effect of the targeted nanoparticles.

Another codelivery system was proposed by Rawal and co-workers [110] who, using DoE, developed a pegylated NLC loaded with CUR and DTX and tested it against NCI-H460 non-small cell lung carcinoma cells. The nanosystem exhibited a high EE (>90% for DTX and CUR). MTT assays revealed that the IC_50_ of DTX/CUR-NLC was lower than that of the free drugs (DTX:CUR, 1:2 molar ratio) or pure DTX. The authors further investigated the NLC functionalisation with FA to target the particles to lung carcinoma cells [91]. The FA-DTX/CUR-NLC formulation showed improved antitumoural effect, with lower IC_50_ and enhanced cell uptake in comparison to the non-functionalised NLC formulation. In a lung carcinoma model (induced by intraperitoneal-injected urethane and butyl-hydroxy-toluene), the bioavailability of DTX was enhanced 12.4 times (4.7 times in the nanoparticles without FA) and was accompanied by a significant decrease in tumour volume in comparison to Taxotere^®^.

Xu et al. [111] developed a NLC formulation loaded with TMZ and CUR (TMZ/CUR-NLC). The EE of the optimised formulation was 71 and 68% for TMZ and CUR, respectively. In vitro cytotoxicity tests in C6 glioma cells showed the synergistic effect of the drug association, with higher cell death rates with TMZ/CUR-NLC when compared with those for TMZ-NLC, CUR-NLC or the free drugs. In the in vivo tumour-bearing mice model (with rat glioma C6 cells), TMZ/CUR-NLC showed a significantly higher antitumour effect (75% decrease in tumour volume) in relation to the naïve group.

### 4.2. Photodynamic and Photothermal Therapy

Photodynamic therapy (PDT) combines light energy with a photosensitiser (PS) to destroy cells after light activation [112]. When irradiated by the light source, the PS absorbs the photon’s energy and goes to the excited state; when decaying to the fundamental state, it transfers energy to O_2_ molecules in the cellular environment, giving rise to ROS, such as triplet oxygen (3O_2_), singlet oxygen (1O_2_), or superoxide anion (O_2_^−^) [112]. These ROS are responsible for the oxidative damage to the target cells, leading to death by apoptosis, necrosis, or autophagy [113,114]. Moreover, when the PS absorbs light (and heat) in the IR region, the therapy is called photothermal. In that special kind of PDT, cell death may arise from mechanisms other than ROS production, such as the increase in local temperature [115]. PDT then allows for spatial and temporal control of drug action, which can be punctually irradiated at established times after absorption.

PDT treatment was proposed in the beginning of the twentieth century [116], but it was in just 1978 that Dougherty et al. [117] carried out the first clinical study with the application of PDT to cancer, with 25 patients with breast, colon, prostate, melanoma, chondrosarcoma, angiosarcoma, endometrial, basal, and squamous cell cancers, all of which were resistant to conventional treatments. The lesions were treated with a hematoporphyrin-derived PS via intravenous administration, followed by local light application (red light, 20 min at 100 milliwatts/sq/cm or its equivalent). The results were quite satisfactory—total or partial remission in 87% of the lesions and complete eradication of metastases involving the chest wall.

Unfortunately, most PSs are hydrophobic, which makes them difficult to administer in aqueous medium. Insufficient solubilisation affects their photophysical properties, reduces their potential to produce ROS, and favours rapid elimination by mononuclear phagocytes [112]. Therefore, PSs are suitable candidates for encapsulation in NLC formulations, considering the aptitude of these nanoparticles to upload hydrophobic drugs in their lipid matrix [9].

In the first reports of NLCs as carriers for PDT, the systems were just tested in vitro. Qidwai et al. [118] developed a NLC for topical delivery of 5-amino levulinic acid, aiming for the treatment of basal cell carcinoma. In vitro release and skin permeation tests showed sustained drug release and greater penetration into the skin, respectively. Whang et al. [119] incorporated the xanthene-indolium derivative DHX-1 into a NLC as a PS against cancer cells. The PS was phototoxic against 4T1 murine breast adenocarcinoma cells and NIH-3T3 fibroblasts in vitro, but encapsulation into NLC formulations significantly reduced DHX-1 phototoxicity against the normal cell line. Oshiro-Junior [120] evaluated the potential of zinc phthalocyanine (ZnPc) encapsulated in NLC formulations functionalised with FA for PDT of breast cancer. Cytotoxicity assays in MCF-7 cells showed a significant decrease in cell viability (57%) after light exposure, substantiating PDT as an alternative breast cancer treatment.

NLC systems for photothermal therapy were also described and tested in vitro and in vivo. Chen et al. [121] reported the development of a NLC suitable for oral delivery containing IR780, a near infrared PS, aiming for the treatment of gastric tumours. The photothermal antitumour activity of IR780-NLC was evaluated after local laser irradiation in a colon tumour (CT-26) xenograft mice model. Photothermal treatment with IR780-NLC reduced the tumour growth rate, with no toxicity associated with oral administration. Li et al. [122] incorporated the hydrophobic IR780 in the inner core of a NLC functionalised with AMD3100 in the outer shell for targeting. AMD3100 is a drug that specifically binds to CXCR4 receptors, which are overexpressed in breast cancer cells, and required for their migration/metastasis. The IR780-AMD-NLCs were efficiently targeted to the tumour cells, reducing their invasive capacity in the lung, after photothermal therapy in a syngeneic (4T1-luc cell line in BALB/c female mice) mouse tumour model.

Almeida et al. [123] investigated SLN and NLC as potential carriers for chlorine aluminium phthalocyanine (ClAlPc), improving its skin penetration and antitumour effect with PDT. In vitro, ClA1Pc-NLC showed higher cytotoxic effects against A549 and B16-F10 melanoma cells than ClA1Pc-SLN of the free PS. At in vivo skin permeation tests in hairless mice, just the ClA1Pc-NLC was able to reach the deeper layers of the skin.

Michy et al. [124] developed a NLC containing the PS verteporfin (VTP) for targeted PTD of ovarian cancer. Cell uptake and phototoxicity of free VTP and VTP-NLC were studied in vitro in human ovarian cancer cell lines cultured in 2D and 3D spheroids. The biodistribution and PDT were evaluated in vivo in NMRI nude mice. Free and encapsulated VTP were internalised in ovarian cancer cells and strongly inhibited the viability of tumour cells when exposed to laser light. However, the administration of free VTP (2 mg/kg) induced severe phototoxicity and death in five out of eight mice. In contrast, exposure of the tumours to laser light after administration of intravenous VTP-NLC (8 mg/kg) significantly inhibited (50%) tumour growth without visible toxicity. Biodistribution and pharmacokinetic studies confirmed the long circulation time associated with efficient tumour uptake of VTP-NLC.

Finally, in 2019, Zhang et al. [125] tested the codelivery of chlorine e6 (C6) and PTX in a NLC functionalised with FA, aiming for the treatment of breast cancer. In vitro (cytotoxicity) and biodistribution tests were performed. In vivo treatments were conducted in MDA-MB-231 tumour xenografts in mice, showing that the non-encapsulated PTX + C6 were able to reduce the tumour volume in less than 10% in relation to the naïve animals, while FA-NLC-C6 (~50%) and FA-NLC-C6-PTX (~75%) showed remarkable tumour reduction rates after irradiation. This report proved, beyond the benefits of encapsulating AAs in NLC, the cooperative effect between chemotherapy and PDT.

## 5. Conclusions

Cancer treatment is a huge and difficult task. Thus far, its pharmacological treatment is based on “specific drugs for specific cancers”, and encapsulation of these drugs of choice in NLC formulations appears to be a powerful strategy to strengthen their efficacy.

**Figure 5:**
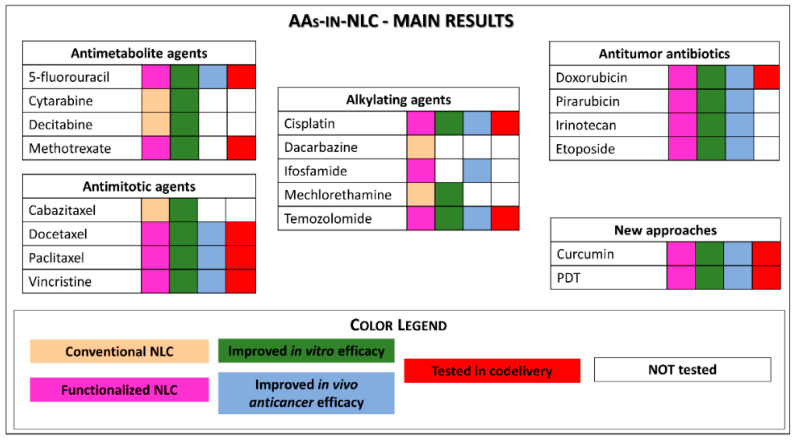
Main results reported for AAs-in-NLC formulations.

Twenty years after the description of NLCs, these lipid-based nanocarriers have shown to be efficient DDS for the encapsulation of the different classes of AAs, resulting in formulations with good physicochemical features and high EE. Figure 5 summarises the main results obtained for the different classes of antineoplastics when encapsulated in NLC formulations. In more than 100 revised publications, AAs-in-NLC formulations exhibited superior cytotoxic activity in vitro in different cancer cells, including drug-resistant lineages. In vivo studies were carried out mostly with xenograft rodent models, in which these formulations increased antitumour activity and reduced systemic toxicity by promoting sustained release, specific targeting (functionalised NLC), and accumulation in tumour tissues (by enhanced permeability retention). Indeed, it is noteworthy that functionalisation was employed in 14 out of the 19 AAs encapsulated in NLC formulations (Figure 5), improving the nanoparticle-specific delivery to the tumour and explaining the superior effects observed in vivo. These results disclose the greater bioavailability and biodistribution promoted by the NLC, leading to efficient and selective AA delivery to the tumour cells. Moreover, NLCs made possible the use of AAs by other routes of administration, and the simultaneous (co)delivery of two or more agents with different mechanisms of action, a strategy that potentiates cancer cell death and may avoid multidrug resistance.

In our view, the available preclinical studies for AAs-in-NLC should be improved, including studies of lipid miscibility (DoE and Raman imaging), a detailed characterisation of the nanoparticles structure (by DSC or XRD), and stronger shelf-stability studies (according to current regulation agencies), to produce reliable systems to be commercialised. However, a matter of current concern is the lack of clinical trials, which is a crucial step for AAs-in-NLC to become a reality in cancer therapy. The efficacy and safety of NLC antineoplastic formulations must be widely demonstrated in humans to overcome the pitfalls of AAs (systemic toxicity, drug resistance, and low bioavailability) and to provide quality of life to patients during and after cancer treatment. We hope these “bench to the bed” studies will emerge in the next couple of years.

## Supplementary Materials

The following are available online. Table S1: List of articles on the encapsulation of antitumor agents in Nanostructured lipid carriers, from 2000–2021. Descriptions include AAs, cancer type, NLC composition, preparation method, physicochemical properties—size, polydispersity (PDI), zeta potential (ZP) and encapsulation efficiency (%EE)—tests conducted and main achievements.

## Abbreviations

AAs—antineoplastic agents; DDS—drug delivery systems; DLS—dynamic light scattering; DHA—docosahexaenoic acid; DoE—design of experiments; DR—drug resistance; 2-DG—stearyl-2-amino-2-deoxyglucose; DSC—differential scanning calorimetry; EE—encapsulation efficiency; EGFP—enhanced green fluorescence protein; FA—folic acid; Flk-1A-3—mouse monoclonal antibody; HA—hyaluronic acid; IC_50_—drug concentration for 50% cell survival; NE—nanoemulsion; NLC—nanostructured lipid carriers; PLT—platelet membrane protein; PS—photosensitizer; R8—poly-argigine peptide; ROS—reactive oxygen species; SLN—solid lipid nanoparticles; XRD—X-ray diffraction.
