# Supplementary material for: Antineoplastics Encapsulated in Nanostructured Lipid Carriers
Source: Molecules. 2021 Nov 17;26(22):6929. doi: 10.3390/molecules26226929 (PMC8619566; doi:10.3390/molecules26226929)
Supplement: Supplementary file 1 [file molecules-26-06929-s001.zip › molecules-1443006-supplementary.pdf]

Table S1 – Articles on the encapsulation of antitumour agents in Nanostructured lipid carriers, from 2000-2021. Descriptions include AA, cancer type, NLC composition, preparation method, physicochemical properties - size, polydispersity (PDI), zeta potential (ZP) and encapsulation efficiency (%EE) - tests conducted and main achievements.

Antimetabolite agents

Drug	Tumour tested and route	Composition	Preparation method	Characterization	Physical-chemical properties	Tests conducted	Main results	Ref.	Ref. in the main text
5-fluorouracil (5-FU)	Hepatocellular carcinoma (**HepG2**) cell line	(Synthetic lipids)<br><br>Solid lipid:<br>GMS<br><br>Liquid lipid:<br>OA or Labrafac 5<br><br>Surfactant:<br>Lecithin<br>Tween 80 or Solutol HS15<br><br>Functionalization:<br>Galactisilated-stearyl amine	Emulsification-solvent diffusion	DLS<br>Microscopy (SEM)<br>DSC<br>FTIR<br>NMR	Size<br>139.2 nm<br><br>PDI<br>0.5<br><br>ZP<br>–18 mV<br><br>%EE<br>34.2%<br><br>Stability:<br>Not informed	Pharm. development<br><br>*In vitro* tests	✓ Improved cytotoxicity<br>✓ Enhanced drug uptake to HepG2 tumour cells	[1]	[27]
5-FU	Not tested	(Synthetic lipids)<br><br>Solid lipid:<br>Precirol ATO 5<br><br>Liquid lipid:<br>Labrasol<br><br>Surfactant<br>P-188<br>Solutol HS15	High-pressure homogenization (HPH)	DLS<br>Microscopy (TEM)	Size<br>211.2±4.9 nm<br><br>PDI<br>0.412±0.065<br><br>ZP<br>−21.6±0.7 mV<br><br>%EE<br>82.42 ± 3.14%	Pharm. development<br><br>*In vitro* tests<br><br>*In vivo:*<br>Skin permeation and irritation	✓ Increased 5-FU permeation<br>✓ Reduced skin irritation	[2]	-

					Stability:<br>Not informed				
5-FU	Not tested	(Synthetic lipids)<br><br>Solid lipid:<br>Tripalmitin<br>Tristearin<br><br>Liquid lipid:<br>Transcutol<br><br>Surfactant:<br>Lecithin<br>Tween 80	High-pressure homogenization (HPH)	DLS<br>Microscopy (TEM)<br>DSC<br>XRD	Size<br>205.8 nm<br><br>PDI<br>0.279<br><br>ZP<br>−30.20 mV<br><br>%EE<br>48.17%<br><br>Stability:<br>Not informed	Pharm. development<br><br>*In vitro* tests<br><br>*In vivo:*<br>Skin permeation	✓ Increased 5-FU permeation	[3]	-
Cytarabine (CYT)	Meningeal leukemia (**EL-4**) cell line	(Synthetic lipids)<br><br>Solid lipid:<br>SA<br><br>Liquid lipid:<br>OA<br><br>Surfactant:<br>Polysorbate 80	Sonication or ultra-sonication	DLS<br>Microscopy (TEM)<br>DSC<br>XRD	Size<br>90.7 nm<br><br>PDI<br>0.16<br><br>ZP<br>-24.2 mV<br><br>%EE<br>49.5%<br><br>Stability:<br>3 months	Pharm. development<br><br>*In vitro* tests	✓ Improved cytotoxicity against EL-4 cells	[4]	[29]
Decitabine (DCB)	Human non-small cell lung cancer (**A549**) cell line	(Synthetic lipids)<br><br>Solid lipid:<br>Precirol ATO5<br><br>Liquid lipid:	High-pressure homogenization (HPH)	DLS<br>Microscopy (AFM and TEM)<br>DSC<br>XRD	Size<br>116.66 nm<br><br>PDI<br>0.194	Pharm. development<br><br>*In vitro* tests<br><br>*Ex vivo*:	✓ Improved cytotoxicity<br>✓ Improved bioavailability after oral administration than intravenously DCB administration<br>✓ Improved gut permeation	[5]	[14]

	Ehrlich Ascites Tumour (**EAT**) bearing mice<br><br>Oral delivery	Transcutol<br><br>Surfactants:<br>Tween 80<br>P-188<br>Solutol HS 15			ZP<br>−31.8 mV<br><br>%EE<br>84.42 ± 5.38%<br><br>Stability:<br>45 days	Gut permeation<br><br>*In vivo:*<br>Biodistribution			
Methotrexate (MTX)	Human prostate cancer (**DU-145)** and ovarian human cancer (**A2780)** cell lines	(Synthetic lipids)<br><br>Solid lipid:<br>Imwitor 812<br><br>Liquid lipid:<br>Neobee<br><br>Surfactant:<br>Cremophor RH40 P-188	Sonication or ultra-sonication	DLS<br>Microscopy (TEM)	Size<br>255.56±15.40<br><br>PDI<br>0.140±0.08<br><br>ZP<br>−25.0±2.70<br><br>%EE<br>60%<br><br>Stability:<br>90 days	Pharm. development<br><br>*In vitro* tests	✓ Improved cytotoxicity in ovarian cancer cells	[6]	[33]
MTX	Not tested	(Synthetic lipids)<br><br>Solid lipid: Witepsol E85<br><br>Liquid lipid: Miglyol 812<br><br>Surfactant:<br>PVA	Sonication or ultra-sonication	DLS<br>Microscopy (TEM)<br>FTIR	Size<br>252 nm<br><br>PDI<br>0.06<br><br>ZP<br>−14 mV<br><br>%EE<br>87%<br><br>Stability:<br>4 weeks	Pharm. development<br><br>*In vitro* tests	✓ No toxicity in fibroblasts<br>✓ Safe application	[7]	[34]

MTX	Human breast cancer cell (**MDA-MB-231)**	(Synthetic lipids)<br><br>Solid lipid:<br>Witepsol E85<br><br>Liquid lipid:<br>Miglyol 812<br><br>Surfactant:<br>PVA<br><br>Functionalization:<br>SPIONs	Sonication or ultra-sonication	DLS	Size<br>214 ± 3 nm<br><br>PDI<br>0.090 ± 0.017<br><br>ZP<br>−12.6 ±1.6 mV<br><br>%EE<br>75%<br><br>Stability:<br>3 months	Pharm. development<br><br>*In vitro* tests	✓ Improved cytotoxicity<br>✓ Hemo-compatibility<br>✓ Caveolae-mediated endocytosis	[8]	[35]
MTX	Tested in normal cells	(Synthetic lipids)<br><br>Solid lipid:<br>cetyl palmitate<br><br>Liquid lipid:<br>Miglyol 812<br><br>Surfactant<br>Polysorbate 80	Sonication or ultra-sonication	DLS<br>Microscopy<br>(TEM / Cryo-SEM)	Size<br>458 nm<br><br>PDI<br>0.260<br><br>ZP<br>-24 mV<br><br>%EE<br>83%<br><br>Stability:<br>3 months	Pharm. development<br><br>*In vitro* tests	✓ Low toxicity in fibroblasts and human keratinocytes	[9]	-

Antimitotic agents

Drug	Tumour tested and route	Composition	Preparation method	Characterization	Physical-chemical properties	Tests conducted	Main results	Ref.	Ref. in the review

Cabazitaxel (CBZ)	Human breast adenocarcinoma (**MDA-MB-468** and **MCF-7**) cell lines	(Synthetic lipids)<br><br>Solid lipid:<br>Precirol ATO5<br><br>Liquid lipid:<br>Labrafac PG<br><br>Surfactant:<br>Tween 80	Microemulsion technique	DLS<br>Microscopy (SEM)<br>DSC<br>XRD<br>FTIR	Size<br>102.90 nm<br><br>PDI<br>0.140<br><br>ZP<br>-23.00 mV<br><br>%EE<br>84.12 ± 1.1%<br><br>Stability:<br>60 days	Pharm. development<br><br>*In vitro* tests	✓ High cellular uptake and apoptosis<br>✓ Improved cytotoxicity	[10]	[38]
Docetaxel (DTX)	Human ovarian carcinoma (**SKOV3**), human prostate cancer (LNCaP, **PC-3** and **DU145**) and **MCF-7** cell lines<br><br>**SKOV3** cell xenograft mouse model<br><br>Intratumourally	(Synthetic lipids)<br><br>Solid lipid:<br>Precirol ATO 5<br><br>Liquid lipid:<br>Labrafil M 1944<br><br>Surfactant:<br>PVA<br>Tween 20<br><br>Functionating:<br>RIPL	Emulsification-solvent evaporation	DLS<br>Microscopy (TEM)<br>DSC	Size<br>240.83 ± 3.44 nm<br><br>PDI<br>0.265<br><br>ZP<br>14.27 ± 0.78 mV<br><br>%EE<br>97.59 ± 0.13%<br><br>Stability:<br>2 weeks	Pharm. development<br><br>*In vitro* tests<br><br>*In vivo:*<br>Tumour growth inhibition (TGI)	✓ Improved tumour inhibition<br>✓ No body weight loss	[11]	-
DTX	Fibrosarcoma (**HT1080**), **MCF-7, A549** and human mouth epidermoid carcinoma (**KB**) cell lines	(Synthetic lipids)<br><br>Solid lipid:<br>Crodamol SS<br><br>Liquid lipid:<br>Crodamol GTCC	Sonication or ultra-sonication	DLS<br>Microscopy (TEM)	Size<br>35.57 ± 0.31 nm<br><br>PDI<br>0.10 ±0.01<br><br>ZP<br>-0.29 ± 0.17 mV	Pharm. development<br><br>*In vitro* tests<br><br>*In vivo:*<br>TGI	✓ Increased cell uptake<br>✓ Improved cytotoxicity<br>✓ Increased tumour cell apoptosis.<br>✓ Greater antitumour efficacy	[12]	-

	**KB** tumour-bearing mice<br><br>Intravenous	Surfactant:<br>Soy lecithin and Solutol HS15			%EE<br>97.47 ±0.90%<br><br>Stability:<br>Not informed				
DTX	Human cells lines: **HepG2**, **SKOV3**, **A549** and murine malignant melanoma (**B16**) cell line<br><br>Mice implanted with **B16** cells<br><br>Intravenous	(Synthetic lipids)<br><br>Solid lipid:<br>GMS<br>SA<br><br>Liquid lipid:<br>OA<br><br>Surfactants:<br>P-188<br>Soya lecithin	Sonication or ultra-sonication	DLS<br>Microscopy (TEM)	Size<br>193.47 ± 5.69 nm<br><br>PDI<br>Not informed<br><br>ZP<br>-33.17 ± 1.20 mV<br><br>%EE<br>89.72 ± 0.89%<br><br>Stability:<br>Not informed	Pharm. development<br><br>*In vitro* tests<br><br>*In vivo:*<br>TGI	✓ Superior antitumour activity<br>✓ Minor side effects compared to Duopafei	[13]	-
DTX	Not tested	(Synthetic lipids)<br><br>Solid lipid:<br>Precifac ATO 5<br><br>Liquid lipid:<br>MCT<br><br>Surfactant:<br>Tween 80<br>Soybean lecithin<br><br>Functionalization:<br>Cysteine-PEG-MSA	Sonication or ultra-sonication	DLS<br>Microscopy (TEM)<br>DSC<br>NMR<br>FTIR<br>XPS	Size<br>96.6 ± 8.0 nm<br><br>PDI<br>0.196 ± 0.004<br><br>ZP<br>−13.72 ± 0.07 mV<br><br>%EE<br>99.27 ± 0.44%<br><br>Stability:<br>Not informed	Pharm. Development<br><br>*In situ:*<br>perfusion experiments<br><br>*In vivo:*<br>Rat biodistribution, and pharmacokinetics	✓ Cysteine increased mucoadhesion<br>✓ Increased bioavailability after oral administration<br>✓ Improved drug absorption	[14]	[43]
DTX	**MCF-7**, **DU 145**,	(Synthetic lipids)	Microemulsion technique	DLS<br>Microscopy (SEM)	Size<br>170.7 ± 3.78 nm	Pharm. development	✓ Improved cytotoxicity	[15]	-

	Glioblastoma (**U-87 MG**) cell lines	Solid lipid:<br>Molten M<br><br>Liquid lipid:<br>Capmul MCM<br><br>Surfactant:<br>Tween 80 Transcutol HP		DSC<br>XRD<br>FTIR	PDI<br>0.192 ± 0.027<br><br>ZP<br>−3.87 ± 0.048 mV<br><br>%EE<br>70.42 ± 3.68%<br><br>Stability:<br>12 months	*In vitro* tests<br><br>*In vivo:*<br>Pharmacokinetic using a rat model administered by intravenous route	✓ Improved bioavailability<br>✓ The *in vivo* toxicological studies of did not show changes organs studied (heart, liver and lungs)		
DTX	**SKOV3** cells<br><br>**SKOV3**-xenografted mouse model<br><br>Intravenous	(Synthetic lipids)<br><br>Solid lipid:<br>Precirol ATO 5<br><br>Liquid lipid:<br>Labrafil M 1944 CS<br><br>Surfactant: Polysorbate 20<br>PVA<br><br>Functionalization:<br>PEGylated -RIPL	Emulsification-evaporation	DLS<br>Microscopy (TEM and CLSM)	Size<br>187.1 ± 6.6 nm<br><br>PDI<br>0.328 ± 0.053<br><br>ZP<br>−17.2 ± 0.2 mV<br><br>%EE<br>94.8 ± 0.2%<br><br>Stability:<br>4 weeks	Pharm. development<br><br>*In vitro* tests<br><br>*In vivo:*<br>Antitumour effects	✓ Increased cell apoptosis.<br>✓ Improved antitumour effect	[16]	[45]
DTX	Not tested	(Synthetic lipids)<br><br>Solid lipid:<br>Precirol ATO 5<br><br>Liquid lipid:<br>MCT<br><br>Surfactant:	Hot High-pressure homogenization	DLS<br>Microscopy (TEM)<br>DSC<br>XRD	Size<br>245.3 ± 13.6 nm<br><br>PDI<br>Not informed<br><br>ZP<br>− 38.8 mV	Pharm. development<br><br>*In vitro* test: release<br><br>*In vivo:*	✓ Improved bioavailability<br>✓ High uptake in reticuloendothelial system organs<br>✓ Concentration of DTX in the lungs was higher	[17]	-

		Soybean lecithin<br>Brij 78			%EE<br>97.8 ± 1.5%<br><br>Stability:<br>6 months	Pharmacokinetic using a rat model administered by intravenous route			
DTX	Cervical cancer (**HeLa**) cell line<br><br>**HeLa** tumour xenograft mice<br><br>Intravenous	(Synthetic lipids)<br><br>Solid lipid:<br>GMS<br><br>Liquid lipid:<br>OA<br><br>Surfactant:<br>PC<br><br>Functionalization:<br>SA-PEG-FA	Sonication or ultra-sonication	DLS<br>Microscopy (TEM)	Size<br>~ 30 nm<br><br>PDI<br>Not informed<br><br>ZP<br>Not informed<br><br>%EE<br>Not informed	Pharm. development<br><br>*In vitro* tests<br><br>*In vivo:*<br>Tissue distribution and antitumour effects	✓ Increased tumour accumulation<br>✓ Higher antitumour efficacy	[18]	[44]
DTX	**HepG2**, **A549** and mouse **B16** cell lines<br><br>Mice with **B16** cells<br><br>Intravenous	(Synthetic lipids)<br><br>Solid lipid:<br>GMS<br>SA<br><br>Liquid lipid:<br>MCT<br><br>Surfactant:<br>P-188<br><br>Functionalization:<br>DSPE-PEG-NH_2	Solvent diffusion method	DLS<br>Microscopy (TEM)	Size<br>168.70 ± 2.07 nm<br><br>PDI<br>0.195 ± 0.01<br><br>ZP<br>− 28.89 ± 1.3 mV<br><br>%EE<br>98.43 ± 0.51%<br><br>Stability:<br>Not informed	Pharm. development<br><br>*In vitro* tests<br><br>*In vivo:*<br>Efficacy, pharmacokinetics and tissue distribution effects	✓ Superior antitumour efficacy against malignant melanoma *in vitro* and *in vivo*	[19]	[42]
DTX	Human lung cancer **(NCL-H460)** cell line	(Synthetic lipids)<br><br>Solid lipid:<br>Precirol ATO 5	High-pressure homogenization	DLS<br>Microscopy (TEM)<br>DSC<br>XRD	Size<br>154.1 ± 3.13 nm<br><br>PDI	Pharm. development<br><br>*In vitro* tests	✓ Improved cytotoxicity	[20]	-

		Liquid lipid:<br>GMO<br><br>Surfactants:<br>Polysorbate 80			0.376 ± 0.04<br><br>%EE<br>86.12 ± 3.48%<br><br>Stability:<br>6 months				
DTX	**HeLa** and **B16** cell lines	(Synthetic lipids)<br><br>Solid lipid:<br>GMS<br><br>Liquid lipid:<br>OA<br><br>Surfactant:<br>P-188<br><br>Gel formulation:<br>Pluronic F127	Sonication or ultra-sonication	DLS<br>Microscopy (TEM)<br>DSC	Size<br>123.5 nm<br><br>PDI<br>0.218<br><br>ZP<br>−33.44 ± 2.54 mV<br><br>%EE<br>80 ± 2.64%<br><br>Stability<br>30 days	Pharm. development<br><br>*In vitro* tests<br><br>*In vivo:*<br>Tissue distribution studies in rats.	✓ Cytotoxicity was lower than free DTX on B16 and HeLa cells.<br>✓ Improved concentration on breast tissue	[21]	-
DTX	**Sarcoma-180** cells in mice<br><br>Intravenous	(Synthetic lipids)<br><br>Solid lipid:<br>GMS<br><br>Liquid lipid:<br>Miglyol 812N<br><br>Surfactant/co-surfactant<br>P-188<br>Soybean lecithin<br><br>Functionalization:	High pressure hot homogenization	MS	Not informed	Pharm. development<br><br>*In vivo*<br>Pharmacokinetics and tissue distribution study using male pathogen-free Sprague–Dawley rats and male Kunming mice	✓ Improved pharmacokinetic profile and distribution in tissues.<br>✓ Successful targeting of DTX, for skin cancer treatment	[22]	-

		FA-PEG-PCHL							
DTX	Human glioblastoma **(U87 MG)** and human grade IV glioblastoma **(BTNW911)** cell lines	(Synthetic lipids)<br><br>Solid lipid: Dynasan Phospholipon 90H<br><br>Liquid lipid: Labrasol Lauroglycol 90 Capryol<br><br>Surfactant: Lipoid S75	Hot homogenization method	DLS<br>NTA<br>Microscopy (TEM)<br>DSC<br>XRD<br>TGA<br>AF4<br>FTIR<br>Raman	Size<br>123.300 ± 0.642 nm<br><br>PDI<br>0.234 ± 0.016<br><br>ZP<br>- 32.400 ± 0.967 mV<br><br>%EE<br>99.130 ± 1.200%<br><br>Stability:<br>6 months	Pharm. development<br><br>*In vitro* tests	✓ Superior cell internalization<br>✓ Improved cytotoxicity<br>✓ Decreased mitochondrial reserve capacity Surpass of the blood-brain tumour barrier	[23]	[41]
DTX	Tested in normal skin	(Synthetic lipids)<br><br>Solid lipid:<br>GMS<br><br>Liquid lipid:<br>CT<br><br>Surfactant:<br>Egg lecithin<br><br>Functionalization:<br>Nicotinamide<br><br>Gel:<br>Carbomer 940	Emulsion evaporation-solidification	DLS<br>Microscopy (CLSM and TEM)<br>PXRD<br>DSC<br>TGA<br>FTIR	Size<br>59.48 ± 4.29 nm<br><br>PDI<br>0.24 ± 0.08<br><br>ZP<br>−31.03± 5.30 mV<br><br>%EE<br>79.48 ± 8.02%<br><br>Stability:<br>Not informed	Pharm. Development<br><br>*In vitro*:<br>*Permeation* in abdominal rat skins<br><br>*In vivo:*<br>skin permeation	✓ *In vitro,* greater skin permeation promoted by DN<br>✓ *In vivo,* DN-NLC gel remained mostly on the inner skin	[24]	-
Paclitaxel (PTX)	**SKOV3** cells	(Synthetic lipids)<br><br>Solid lipid:	Sonication or ultra-sonication	DLS<br>Microscopy	Size<br>115.20 ± 3.90 nm	Pharm. development	✓ Improved cytotoxicity	[25]	[54]

		GMS<br><br>Liquid lipid:<br>Capryol 90<br><br>Surfactant:<br>P-188<br>Tween 80<br><br>Functionalization:<br>Platelet membrane		(CLSM / TEM / STEM-EDS)<br>DSC<br>PXRD	PDI<br>0.284 ± 0.015<br><br>ZP<br>-15.00 ± 0.93 mV<br><br>%EE<br>99.88 ± 0.01%<br><br>Stability:<br>Not informed	*In vitro* tests			
PTX	**MCF-7** cells<br><br>Mice bearing **MCF-7** tumours<br><br>Intravenous	(Synthetic lipids)<br><br>Solid lipid:<br>GMS<br><br>Liquid lipid:<br>OA<br><br>Surfactant:<br>Tween 20<br><br>Functionalization:<br>2-DG	Sonication or ultra-sonication	Microscopy (TEM / CLSM)<br>DSC<br>NIR<br>XRD<br>LPSA	Size<br>135.00 nm<br><br>PDI<br>~0.270<br><br>ZP<br>- 21.8 ± 1.60 mV<br><br>%EE<br>95.50%<br><br>Stability:<br>90 days	Pharm. development<br><br>*In vitro* tests<br><br>*In vivo:*<br>Efficacy	✓ high anticancer potential<br>✓ lower systemic toxicity	[26]	[48]
PTX	Human colorectal adenocarcinoma (**HT-29**) cell line	(Synthetic lipids)<br><br>Solid lipid:<br>Chol<br><br>Liquid lipid:<br>OA<br><br>Surfactant:<br>P-188	Sonication or ultra-sonication	DLS<br>Microscopy (AFM)	Size<br>181.6 ± 14.8 nm<br><br>PDI<br>~0.10<br><br>ZP<br>−12.9 ± 0.2 mV<br><br>%EE	Pharm. development<br><br>*In vitro* tests	✓ Improved cytotoxicity	[27]	-

					53 ± 4.2%<br><br>Stability:<br>Not informed				
PTX	**HepG2** cells	(Natural lipids)<br><br>Solid lipid:<br>GMS<br><br>Liquid lipid:<br>Soybean oil<br>Chol<br><br>Surfactant:<br>Tween 80<br>P-188	Sonication or ultra-sonication	DLS<br>Microscopy (TEM)	Size<br>153.82 ± 5.58 nm<br><br>PDI<br>0.221 ± 0.02<br><br>ZP<br>−22.12 ± 2.48 mV<br><br>%EE<br>86.48 ± 6.46%<br><br>Stability:<br>Not informed	Pharm. development<br><br>*In vitro* tests<br><br>*Ex vivo:*<br>Intestinal permeation<br><br>*In vivo:*<br>Pharmacokinetic study in Wistar rats administrated orally	✓ Improved cytotoxicity<br>✓ Improvement in oral bioavailability	[28]	-
PTX	Human epithelial colorectal adenocarcinoma cell line (**Caco 2**)	(Synthetic lipids)<br><br>Solid lipid:<br>GMS<br><br>Liquid lipid:<br>OA<br><br>Surfactant:<br>Tween 20	Sonication or ultra-sonication	DLS<br>Microscopy	Size<br>283.4 ± 4.5 nm<br><br>PDI<br>0.226 ± 0.94<br><br>ZP<br>-25.12 mV<br><br>%EE<br>85.60%<br><br>Stability:<br>Not informed	Pharm. development<br><br>*In vitro* tests<br><br>*In vivo:*<br>Organ distribution studies after pulmonary administration	✓ Maximum cell uptake by **Caco-2** cells<br>✓ Improved pulmonary bioavailability	[29]	[50]
PTX	Breast cancer (**MCF-7** and **MDA-MB-231)** cell lines	(Synthetic lipids)<br><br>Solid lipid:	Hot melting homogenization	DLS<br>Microscopy (PLM)<br>DSC	Size<br>67 ± 15 nm	Pharm. development	✓ Improved cytotoxicity	[30]	-

		ATO<br><br>Liquid lipid:<br>Chol<br>Glycerol<br><br>Surfactant: Polysorbate 80			PDI<br>0.30 ± 0.12<br><br>ZP<br>− 14.5 ± 1.4 mV<br><br>%EE<br>90%<br><br>Stability:<br>30 days	*In vitro* tests			
PTX	**A549** cells	(Synthetic lipids)<br><br>Solid lipid:<br>GMS<br><br>Liquid lipid:<br>MCT<br><br>Surfactant:<br>Kolliphor HS15<br>Gelucire 44/14<br><br>Functionalization:<br>SA-R8	Fusion-emulsification method	DLS<br>Microscopy (CLSM)	Size<br>42.34 ± 0.87 nm<br><br>PDI<br>0.2 ± 0.01<br><br>ZP<br>16.84 ± 0.96 mV<br><br>%EE<br>65.94%<br><br>Stability:<br>Not informed	Pharm. development<br><br>*In vitro* tests	✓ Improved cytotoxicity	[31]	[52]
PTX	**MCF-7**, **A549** and **HeLa** cell lines	(Synthetic lipids)<br><br>Solid lipid:<br>SA<br><br>Liquid lipid:<br>OA<br><br>Surfactant:<br>P-188	Solvent diffusion method	DLS<br>Microscopy (TEM)	Size<br>237.10 nm<br><br>PDI<br>0.27<br><br>ZP<br>-33.9 mV<br><br>%EE<br>49%	Pharm. development<br><br>*In vitro* tests<br><br>*In vivo:*<br>biodistribution in *Wistar* rats after intravenous injection	✓ higher cell uptake<br>✓ *in vivo* higher uptake in folate receptor-positive organs	[32]	[53]

		Functionalization:<br>Fol-PEG-CHEMS			Stability:<br>Not informed				
PTX	Murine melanoma (**B16**), mouse colon cancer (**CT26**) and human colon cancer (**HCT116**) cell lines<br><br>**B16** in mice<br><br>Intravenous	(Synthetic /Natural lipids)<br><br>Solid lipid:<br>GMS<br><br>Liquid lipid: Soybean oil<br><br>Surfactant:<br>Soya lecithin<br><br>Functionalization:<br>HA	Hot emulsion method	DLS<br>Microscopy (TEM)	Size<br>197.47 ± 8.24 nm<br><br>PDI<br>0.26 ± 0.03<br><br>ZP<br>-39.61 ± 0.69 mV<br><br>%EE<br>90.25 ± 0.41%<br><br>Stability:<br>Not informed	Pharm. development<br><br>*In vitro* tests<br><br>*In vivo:*<br>Efficacy, pharmacokinetics and tissue distribution studies in rats.	✓ Improved cytotoxicity<br>✓ Greater antitumour efficacy<br>✓ Prolonged circulation time in the blood<br>✓ Increased accumulation	[33]	[49]
PTX	**HepG2** cells<br><br>**H22** (murine liver cancer carcinoma cells) tumour xenograft mice<br><br>Intravenous	(Synthetic lipids)<br><br>Solid lipid:<br>GMS<br><br>Liquid lipid:<br>OA<br><br>Surfactant:<br>PEG-SA<br><br>Functionalization:<br>SA-polyethylene glycol-folate	Microemulsion technique	DLS<br>Microscopy (TEM)	Size<br>25 ± 2.5 nm<br><br>PDI<br>0.102<br><br>ZP<br>Not informed<br><br>%EE<br>Not informed<br><br>Stability:<br>Not informed	Pharm. development<br><br>*In vitro* tests<br><br>*In vivo:*<br>Tumour penetration and anti-tumour studies	✓ Improved cytotoxicity<br>✓ Improved *in vivo* effect<br>✓ Low systemic toxicity	[34]	-
PTX	Human lung cancer **(NCl-H1299)** and murine sarcoma	(Synthetic lipids)<br><br>Solid lipid:<br>ATO	Emulsification–evaporation	DLS<br>Microscopy (TEM and CLSM)	Size<br>63.27 ± 1.14 nm<br><br>PDI	Pharm. development<br><br>*In vitro* tests	✓ Increased cell uptake by tumour cells and decreased recognition by macrophages	[35]	-

	cancer **(S180)** cell lines<br><br>**S180** tumour-bearing mice<br><br>Intravenous	GMS<br><br>Liquid lipid:<br>MCT<br><br>Surfactant:<br>Kolliphor ELP and HS15<br><br>Functionalization:<br>RBCm			0.095 ± 0.021<br><br>ZP<br>-29.56 ± 1.17 mV<br><br>%EE<br>93.44 ± 0.17%<br><br>Stability:<br>15 days	*In vivo:*<br>Tumour-targeting effect and anti-tumour studies was performed in mice.	✓ prolonged PTX blood circulation<br>✓ Increased the antitumour effect<br>✓ extended the survival period<br>✓ lower collateral effects.		
PTX	**U-87** cells	(Synthetic lipids)<br><br>Solid lipid:<br>Chol<br><br>Liquid lipid<br>Triolein<br>Soy lecithin<br><br>Surfactant:<br>P-188<br><br>Functionalization:<br>Transferrin	Emulsification-solvent evaporation	PCS<br>Microscopy (TEM)<br>XRD	Size<br>205.4 ± 11 nm<br><br>PDI<br>Not informed<br><br>ZP<br>25.7 ± 6.22mV<br><br>%EE<br>91.8 ± 0.5%<br><br>Stability:<br>Not informed	Pharm. development<br><br>*In vitro* tests	✓ Increased cytotoxicity<br>✓ Higher cellular uptake	[36]	[51]
Vincristine sulfate (VCR)	**MCF-7** cells	(Synthetic lipids)<br><br>Solid lipid:<br>GMS<br><br>Liquid lipid:<br>CT<br><br>Surfactants:<br>SDS<br>Egg lecithin	Emulsion solvent evaporation method	DLS<br>Microscopy (TEM / CLSM)	Size<br>192 ± 4.41 nm<br><br>PDI<br>0.184 ± 0.05<br><br>ZP<br>26.3 ± 1.44 mV<br><br>%EE<br>33.28 ± 2.31%	Pharm. development<br><br>*In vitro* tests<br><br>*In vivo:*<br>Pharmacokinetic in rats with oral administration	✓ Improved cytotoxicity<br>✓ HA induced cell apoptosis.<br>✓ Superior cell uptake *in vitro*<br>✓ Improved oral bioavailability	[37]	[16]

		Tween 80<br>CTAB<br><br>Functionalization:<br>HA			Stability:<br>Not informed				

Alkylating agents

Drug	Tumour tested and route	Composition	Preparation method	Characterization	Physical-chemical properties	Tests conducted	Main results	Ref.	Ref. in the review
Cisplatin (CIS)	**HeLa** cells<br><br>**HeLa** mice-bearing xenograft<br><br>Intravenous	(Natural lipids)<br><br>Solid lipid:<br>SA<br><br>Liquid lipid:<br>Soybean oil<br><br>Surfactant:<br>Tween-80<br>Soya lecithin<br><br>Functionalization:<br>FA-PEG-DSPE	Emulsification method	DLS	Size<br>143.2 ± 5.3 nm<br><br>PDI<br>0.15 ± 0.02<br><br>ZP<br>25.7 ± 2.3mV<br><br>%EE:<br>87.5 ± 3.2%<br><br>Stability:<br>Not informed	Pharm. Development<br><br>*In vitro* tests<br><br>*In vivo:*<br>Anti-tumour efficiency in mice	✓ Superior cytotoxicity and tumour inhibition	[38]	[60]
CIS	**SKOV3** cells<br><br>**SKOV3** cells in mice<br><br>Intravenous	(Synthetic lipids)<br><br>Solid lipid:<br>ATO<br>Precirol ATO 5<br><br>Liquid lipid:<br>Miglyol 812<br><br>Surfactant:	Sonication or ultra-sonication	DLS<br>Microscopy (TEM)	Size<br>132.4 ± 5.3nm<br><br>PDI<br>0.158 ± 0.015<br><br>ZP<br>−19.3 ± 1.9mV<br><br>%EE:	Pharm. Development<br><br>*In vitro* tests<br><br>*In vivo:*<br>Pharmacokinetics, biodistribution and antitumour efficiency	✓ Improved cytotoxicity and cellular uptake<br>✓ Sustained plasma retention until 48h<br>✓ Best tumour inhibition rate	[39]	-

		Tween 80<br>Lecithin			82.6 ± 3.9%<br><br>Stability:<br>Not informed				
Dacarbazine (DZ)	Not tested	(Synthetic lipids)<br><br>Solid lipid:<br>Glyceryl palmitostearate<br>Isopropyl myristate<br><br>Liquid lipid:<br>TS<br>Polyethylene glycol succinate<br><br>Surfactant:<br>P-188<br>Soybean lecithin	Sonication or ultra-sonication	DLS<br>Microscopy (TEM)	Size:<br>190 ± 10 nm<br><br>PDI:<br>0.2 ± 0.01<br><br>ZP:<br>Not informed<br><br>%EE:<br>98.5%<br><br>Stability:<br>Not informed	Pharm. development	NLC with high encapsulation efficiency	[40]	[62]
Ifosfamide (IFOS)	Not tested	(Synthetic lipids)<br><br>Solid lipid:<br>GMO<br><br>Liquid lipid:<br>OA<br><br>Surfactant:<br>P-188<br><br>Functionalization:<br>Chitosan and Sodium alginate	Sonication or ultra-sonication	DLS<br>Microscopy (TEM)<br>FTIR<br>DSC<br>XRD	Size<br>223 nm<br><br>PDI<br>0.194 ± 0.025<br><br>ZP<br>−25 mV<br><br>%EE:<br>77%<br><br>Stability:<br>6 months	Pharm. Development	✓ Enhanced NLC stability<br>✓ High entrapment efficiency<br>✓ Sustained release	[41]	[63]
IFOS	Dalton's ascitic lymphoma (**DAL**) cells in mice	(Synthetic lipids)<br><br>Solid lipid:	Sonication or ultra-sonication	DLS	Size<br>223 nm	*In vivo:*	✓ Increased IFOS plasma concentrations	[42]	[18]

	Oral route	GMO<br><br>Liquid lipid:<br>OA<br><br>Surfactant:<br>P-188<br><br>Functionalization:<br>Chitosan and Sodium alginate			PDI<br>0.194 ± 0.025<br><br>ZP<br>−25 mV<br><br>%EE:<br>77%<br><br>Stability:<br>6 months	Pharmacokinetics study and Antitumour Activity	and bioavailability.<br>✓ Oral administration as efficient as intravenous administration of IFOS suspension, but with less side effects		
IFOS	Not tested	(Synthetic lipids)<br><br>Solid lipid:<br>GMO<br><br>Liquid lipid:<br>OA<br><br>Surfactant:<br>P-188<br><br>Functionalization:<br>Chitosan and Sodium alginate	Sonication or ultra-sonication	DLS	Size<br>223 nm<br><br>PDI<br>0.194 ± 0.025<br><br>ZP<br>−25 mV<br><br>%EE:<br>77%<br><br>Stability:<br>6 months	*In vivo:*<br>Toxicity after oral delivery in rat	✓ No mortality<br>✓ No signs of behavioural changes or toxicity after oral administration of IIFOS NLC<br>✓ Reduced severity of renal dysfunction induced by IFOS	[43]	[64]
Mechlorethamine (MCTN)	Myelogenous leukemia (**K562**) cell line	(Synthetic lipids)<br><br>Solid lipid:<br>ATO<br><br>Liquid lipid:<br>Miglyol 812<br><br>Surfactant:	Sonication or ultra-sonication	DLS<br>Microscopy (TEM)<br>DSC	Size<br>105.92 ± 1.04 nm<br><br>PDI<br>0.02<br><br>ZP<br>- 30.1 ± 0.82 mV	Pharm. Development<br><br>*In vitro* tests<br><br>*In vivo:*<br>Pharmacokinetics study in rat by oral route	✓ Improved cytotoxicity<br>✓ Increased MCT plasma concentration<br>✓ Greater absorption	[44]	[17]

		P-188			%EE:<br>91.7 ± 1.20%<br><br>Stability:<br>180 days				
Temozolomide (TMZ)	Not tested	(Synthetic lipids)<br><br>Solid lipid:<br>Gelucire 44/14<br><br>Liquid lipid:<br>Trascutol<br><br>Surfactant:<br>Tween 80<br><br>Functionalization:<br>Vitamin E	High-pressure homogenization (HPH) followed by ultrasonication	PCS<br>Microscopy (TEM)<br>DSC	Size<br>131.58 nm<br><br>PDI<br>0.177<br><br>ZP<br>15.21 ± 3.11 mV<br><br>%EE:<br>81.64 ± 3.71%<br><br>Stability:<br>Not informed	Pharm. development<br>*Ex Vivo*: Transport Study Across Nasal Mucosa<br><br>*In vivo:*<br>(Wistar rats) absorption and brain uptake/ Pharmacokinetic and Brain Distribution	✓ Greater permeation through the nasal mucosa<br>✓ increased nose-to-brain drug delivery<br>✓ Long lasting (24h) effect<br>✓ Tween 80 enhanced drug penetration	[45]	[66]
TMZ	**U87 MG** cells<br><br>**U87 MG** in mice<br><br>Intravenous	(Synthetic lipids)<br><br>Solid lipid:<br>ATO<br><br>Liquid lipid:<br>Cremophor<br><br>Surfactant:<br>Tween-80<br>Soya lecithin	Emulsification-solvent diffusion	DLS<br>Microscopy (TEM)	Size<br>121.4±5.6 nm<br><br>PDI<br>0.21±0.06<br><br>ZP<br>29.1±2.4 mV<br><br>%EE:<br>81.4 ± 3.7%<br><br>Stability:<br>Not informed	Pharm. Development<br><br>*In vitro* tests<br><br>*In vivo:*<br>Anti-tumour efficacy	✓ Improved cytotoxicity<br>✓ High tumour regression rates	[46]	[67]
TMZ	Malignant glioma<br><br>Intravenous	(Synthetic lipids)<br><br>Solid lipid:	Emulsification-solvent diffusion	DLS	Size<br>178.9 ± 2.7 nm	Pharm. Development<br><br>*In vitro* tests	✓ Higher cytotoxicity	[47]	[68]

		ATO<br><br>Liquid lipid:<br>Cremophor ELP<br><br>Surfactant:<br>DDAB<br>Lecithin<br>Tween-80			PDI<br>0.16 ± 0.05<br><br>ZP:<br>22.8 ± 2.8 mV<br><br>%EE:<br>82.7 ± 2.5%<br><br>Stability<br>Not informed	*In vivo:*<br>Gene transfection analysis<br>Anti-tumour efficacy	✓ Higher tumour inhibition rates		
TMZ	**U87 MG** cells<br><br>**U87 MG** solid tumours in mice<br><br>Intravenous	(Synthetic lipids)<br><br>Solid lipid:<br>ATO<br><br>Liquid lipid:<br>Cremophor<br><br>Surfactant:<br>Tween-80<br>Soya lecithin<br><br>Functionalization:<br>RGD	Emulsification-solvent diffusion	DLS	Size<br>118.3 ± 2.6 nm<br><br>PDI<br>0.11 ± 0.02<br><br>ZP<br>28.9 ± 2.9 mV<br><br>%EE:<br>84.7 ± 3.2%<br><br>Stability:<br>Not informed	Pharm. Development<br><br>*In vitro* tests<br><br>*In vivo:*<br>Anti-tumour efficacy	✓ Significantly higher cytotoxicity<br><br>✓ Superior tumour growth inhibition	[48]	[69]
Oxaliplatin (OXA)	**HT-29** cells	(Synthetic lipids)<br><br>Solid lipid:<br>ATO<br><br>Liquid lipid:<br>Miglyol 812<br><br>Surfactant:<br>Poloxamer	Sonication or ultra-sonication	DLS<br>Microscopy (SEM)	Size<br>~ 98nm<br><br>PDI<br>0.2<br><br>ZP<br>-11,4mV<br><br>%EE:<br>87%	Pharm. Development<br><br>*In vitro* tests	✓ Higher inhibition of cell proliferation,<br>✓ Increased cell death and apoptosis	[49]	-

		Functionalization:<br>Naringenin			Stability:<br>Not informed				

Antitumour antibiotics

Drug	Tumour tested and route	Composition	Preparation method	Characterization	Physical-chemical properties	Tests conducted	Main results	Ref.	Ref. in the review
Doxorubicin (DOX)	**MCF-7** cells	(Synthetic lipids)<br><br>Solid lipid:<br>SA<br><br>Liquid lipid:<br>OA<br><br>Surfactant<br>Lecithin<br>P-188	Emulsification-solvent evaporation	DLS<br>Microscopy (SEM)	Size<br>134.0 ± 2.3nm<br><br>PDI<br>0.265 ± 0.031nm<br><br>ZP<br>−15 ± 3.4 to −29.4 ± 7.3 mV<br><br>%EE<br>75<br><br>Stability:<br>60 days	Pharm. Development<br><br>*In vitro* tests	✓ Improved cytotoxicity	[50]	**-**
DOX	Murine breast cancer cell line (**4T1**)<br><br>**4T1** in mice<br><br>Intravenous	(Natural lipids)<br><br>Solid lipid:<br>ATO<br><br>Liquid lipid:<br>Sesame oil<br><br>Surfactant<br>Tween 80	Sonication or ultra-sonication	DLS	Size<br>93.76 nm<br><br>PDI<br>0.262<br><br>ZP<br>-29.9<br><br>%EE<br>96.54%	Pharm. Development<br><br>*In vitro* tests<br><br>*In vivo:*<br>Antitumour activity	✓ Improved cytotoxicity<br>✓ Improved antitumoral efficiency compared to other DDS.	[51]	[76]

					Stability:<br>Not informed				
DOX	**MDA-MB-231** and **4T1** cells<br><br>**4T1** in mice<br><br>Intravenous	(Synthetic lipids)<br><br>Solid lipid:<br>ATO<br><br>Liquid lipid:<br>OA<br><br>Surfactant<br>Tween 80<br><br>Functionalization:<br>DHA<br>TS	Sonication or ultra-sonication	DLS	Size<br>72.5 ± 2.6nm<br><br>PDI<br>0.139 ± 0.006<br><br>ZP<br>- 21.7 ± 2.2 mV<br><br>%EE<br>95.7 ± 3.3%<br><br>Stability:<br>Not informed	Pharm. Development<br><br>*In vivo:*<br>Antitumour Activity, Biodistribution, histology and toxicity	✓ Improved cytotoxicity<br>✓ Improved antitumoral efficiency with reduced toxicity.	[52]	[77]
DOX	**MDA-MB-231** and **4T1** cells<br><br>**4T1** tumour-bearing mice<br><br>Intravenous	(Synthetic lipids)<br><br>Solid lipid:<br>ATO<br><br>Liquid lipid:<br>MCT<br><br>Surfactant<br>Tween 80<br><br>Functionalization: TS	Sonication or ultra-sonication	DLS<br>NTA<br>Microscopy (AFM)	Size<br>85.0 ± 4.1nm<br><br>PDI<br>0.204 ± 0.02<br><br>ZP<br>−41.0 ± 2.6 mV<br><br>%EE<br>98.97 ± 0.33%<br><br>Stability:<br>Not informed	Pharm. Development<br><br>*In vitro* tests<br><br>*In vivo:*<br>Pharmacokinetics, biodistribution and antitumour activity	✓ Improved cytotoxicity DOX/TS synergy)<br>✓ Improved bioavailability and antitumour activity	[53]	[78]
DOX	**A549** cells	(Synthetic lipids)<br><br>Solid lipid:<br>Precirol ATO 5<br><br>Liquid lipid:	High-pressure homogenization (HPH)	DLS<br>Microscopy (SEM)	Size<br>86.2 nm<br><br>PDI<br>0.28	Pharm. Development<br><br>*In vitro* tests	✓ Improved cytotoxicity<br>✓ Improved cellular internalization<br>✓ Induced cellular apoptosis	[54]	-

		Miglyol<br><br>Surfactant<br>Poloxamer 407<br><br>Functionalization:<br>SiC<br>RGD			ZP<br>−18.5 mV<br><br>%EE<br>56.04 ± 1.25%<br><br>Stability:<br>8 weeks				
DOX	**A549** cells<br><br>**A549** solid tumours in mice<br><br>Intravenous	(Natural lipids)<br><br>Solid lipid:<br>Precirol ATO-5<br><br>Liquid lipid:<br>Olive oil<br>Lipoid S100<br><br>Surfactant<br>Tween-80<br>Lecithin<br><br>Functionalization:<br>pEGFP-N1	Emulsification-solvent evaporation	DLS<br>Microscopy (TEM)	Size<br>198.2 ± 3.1nm<br><br>PDI<br>0.13 ± 0.02<br><br>ZP<br>18.9 ± 2.6 mV<br><br>%EE<br>86.7 ± 2.7%<br><br>Stability:<br>Not informed	Pharm. Development<br><br>*In vitro* tests<br><br>*In vivo:*<br>Gene transfection analysis and antitumour effect in mice	✓ Improve gene transfection efficiency of the vector<br>✓ Improved efficacy on solid cancer	[55]	[81]
DOX	**4T1** cells<br><br>**4T1** tumour-bearing mice<br><br>Intravenous	(Synthetic lipids)<br><br>Solid lipid:<br>ATO<br><br>Liquid lipid:<br>Docosahexaenoic triglyceride<br><br>Surfactant:<br>Tween 80<br><br>Functionalization:	Sonication or ultra-sonication	DLS<br>NTA<br>Microscopy (TEM)<br>DSC<br>SAXS	Size<br>85 ± 3 nm<br><br>PDI<br>0.22 ± 0.01<br><br>ZP<br>− 41 ± 1 mV<br><br>%EE<br>81 ± 2%<br><br>Stability:	Pharm. Development<br><br>*In vitro* tests<br><br>*In vivo:*<br>Evaluation of antitumour activity and toxicity evaluation in mice	✓ DOX, DHA, and TS synergistic effects<br>✓ Increased cellular uptake<br>✓ Improved tumour inhibition and reduced mortality, metastasis and toxicity in liver and heart	[56]	[80]

		DHA<br>TS			Not informed				
DOX	**4T1** cells<br><br>**4T1** tumour-bearing mice<br><br>Intravenous	(Synthetic lipids)<br><br>Solid lipid:<br>Cholesteryl ester<br>Triolein<br><br>Liquid lipid:<br>OA<br><br>Surfactant:<br>CTAB<br><br>Functionalization:<br>ApoB-100	Emulsification-solvent evaporation	DLS<br>Microscopy<br>(CLSM and TEM)	Size<br>129.0 ± 2.4 nm<br><br>PDI<br>0.19 ± 0.02<br><br>ZP<br>- 21 ± 0.82 mV<br><br>%EE<br>91.4 ± 1.9%<br><br>Stability:<br>Not informed	Pharm. Development<br><br>*In vitro* tests<br><br>*In vivo:*<br>Bio-distribution, antitumour efficiency and toxicity	✓ Higher tumour cell uptake and tumour cell inhibition<br>✓ Improved in vivo efficacy with lower toxicity	[57]	[79]
DOX	**B16F10** cells	(Synthetic lipids)<br><br>Solid lipid:<br>ATO<br><br>Liquid lipid:<br>Miglyol 812N<br><br>Surfactant:<br>Poloxamer<br><br>Functionalization:<br>Stattic	Sonication or ultra-sonication	DLS<br>Microscopy<br>(SEM	Size<br>67–115 nm<br><br>PDI<br>0.24<br><br>ZP<br>30 mV<br><br>%EE<br>Not informed<br><br>Stability:<br>Not informed	Pharm. Development<br><br>*In vitro* tests	✓ Synergistic activity<br>✓ Inhibition of STAT3 signalling/ downregulation of antiapoptotic Bcl-2 family genes	[58]	-
DOX	**MDA-MB-231** cells	(Synthetic lipids)<br><br>Solid lipid:<br>Gelucire<br>Cetyl palmitate	Sonication or ultra-sonication	DLS and ELS<br>Microscopy<br>(TEM)	Size<br>281 ± 18 nm<br><br>PDI<br>0.15 ± 0.04	Pharm. Development<br><br>*In vitro* tests	✓ Improved cytotoxicity and cellular uptake	[59]	-

		Liquid lipid:<br>OA<br>Miglyol<br><br>Surfactant:<br>Polysorbate 80<br><br>Functionalization:<br>PEG-FA			ZP<br>− 28.0 ± 0.9 mV<br><br>%EE<br>66 ± 8%<br><br>Stability:<br>42 days				
DOX	**MCF-7** and **MCF7/ADR** cells	(Synthetic lipids)<br><br>Solid lipid:<br>ATO<br><br>Liquid lipid:<br>OA<br><br>Surfactant:<br>Tween 80<br><br>Functionalization:<br>DHA<br>.	Sonication or ultra-sonication	DLS<br>Microscopy (CLSM)	Size<br>76–86 nm<br><br>PDI<br>0.22<br><br>ZP<br>−23 and −36 mV<br><br>%EE<br>~100<br><br>Stability:<br>Not informed	*In vitro* tests<br><br>Spheroids:<br>Cytotoxicity<br>Penetration	✓ Improved cytotoxicity and penetration in spheroids	[60]	[74]
DOX	**4T1** cells<br><br>**4T1** tumour-bearing mice<br><br>Intravenous	(Synthetic lipids)<br><br>Solid lipid:<br>ATO<br><br>Liquid lipid:<br>OA<br>DHA<br><br>Surfactant<br>Tween 80<br><br>Functionalization:	Sonication or ultra-sonication	DLS<br>Microscopy (SEM / TEM)	Size<br>128 ± 1 nm<br><br>PDI<br>0.098<br><br>ZP<br>5 ± 3 mV<br><br>%EE<br>Not informed<br><br>Stability:	Pharm. Development<br><br>*In vitro* tests<br><br>*In vivo:*<br>Tumour growth inhibition, toxicity and pharmacokinetic	✓ Improved pharmacokinetics (higher plasma concentration)<br>✓ lower accumulation in the liver and higher accumulation in tumour<br>✓ Enhanced effect (tumour growth inhibition)	[61]	[75]

		LbL-PEG			14 days				
DOX	**MCF-7** cells	(Synthetic lipids)<br><br>Solid lipid:<br>ATO<br><br>Liquid lipid:<br>Miglyol 812N<br><br>Surfactant:<br>Poloxamer 407<br><br>Functionalization:<br>Vitamin D	Sonication or ultra-sonication	DLS<br>Microscopy (SEM)<br>FTIR	Size<br>87.5 nm<br><br>PDI<br>0.24<br><br>ZP<br>-12 mV<br><br>%EE<br>Not informed<br><br>Stability:<br>Not informed	Pharm. Development<br><br>*In vitro* tests	✓ Improved cytotoxicity<br>✓ Increased percentage of MCF-7 cells in sub-G1 arrest	[62]	-
DOX	**PC-3** cells	(Natural lipids)<br><br>Solid lipid:<br>GMS<br><br>Liquid lipid:<br>Soybean oil<br><br>Surfactant:<br>Lecithin<br>PEG-40-St<br>DSPE-PEG	Emulsification-solvent evaporation	DLS<br>Microscopy (TEM)	Size<br>170 nm<br><br>PDI<br>Not informed<br><br>ZP<br>−14.8 mV<br><br>%EE<br>74.18 ± 0.3%<br><br>Stability:<br>Not informed	Pharm. Development<br><br>*In vitro* tests<br><br>*In vivo:*<br>Oral bioavailability	✓ Improved cytotoxicity<br>✓ Improved DOX bioavailability	[63]	[19]
DOX	**HCT116** cells	(Synthetic lipids)<br><br>Solid lipid:<br>SA<br>GMS	High-pressure homogenization (HPH)	DLS<br>Microscopy (TEM)<br>DSC<br>SAXS	Size<br>168 − 173 nm<br><br>PDI<br>was less than 0.2<br><br>ZP	Pharm. Development<br><br>*In vitro* tests	✓ Improved cytotoxicity<br>✓ Higher cellular uptake	[64]	-

		Liquid lipid:<br>OA<br>MCT<br><br>Surfactant<br>P-188			30 mV<br><br>%EE<br>97.80%<br><br>Stability:<br>30 days				
Pirarubicin (THP)	**4T1** cell<br><br>Mice bearing **4T1**<br>Intravenous	(Synthetic lipids)<br><br>Solid lipid:<br>Structured triglyceride<br><br>Liquid lipid:<br>OA<br><br>Surfactant:<br>Solutol HS 15<br>Lecithin<br><br>Functionalization:<br>TS	High-pressure homogenization (HPH)	DLS<br>Microscopy<br>(TEM / CLSM)	Size<br>112 ± 2.8 nm<br><br>PDI<br>0.18 ± 0.02<br><br>ZP<br>−17.5 ± 1.3 mV<br><br>%EE<br>90.01 ± 3.03%<br><br>Stability:<br>Not informed	Pharm. Development<br><br>*In vitro* tests<br><br>*In vivo:*<br>Distribution and pharmacokinetic studies, flow cytometry (Analysis of Immune cells and cytokine Assay), histological and immunohistoche mical analysis, antitumour efficacy<br>in mice Co-administering iRGD	✓ Higher levels of tumour distribution,<br>✓ enhanced tumour cell death<br>✓ increased immune-mediated antitumour effect	[65]	[82]
Irinotecan (Ir)	**HT-29** and **Colo-320** cell lines<br><br>Ehrlich's ascites tumour in mice<br><br>Intravenous	(Synthetic lipids)<br><br>Solid lipid:<br>ATO<br><br>Liquid lipid:<br>Capmul MCM	Emulsification-solvent evaporation	DLS<br>Microscopy<br>(CLSM / TEM)<br>FTIR<br>NMR	Size<br>386 ± 2.2 nm<br><br>PDI<br>Not informed<br><br>ZP<br>19.7 ± 1.2 mV	Pharm. Development<br><br>*In vitro* tests<br><br>*In vivo:*<br>biodistribution studies	✓ Low macrophage engulfment<br>✓ Enhanced uptake and prolonged accumulation in in vivo	[66]<br>[67]	[84]<br>[85]

		Surfactant<br>P-188 Deoxycholate<br>Lecithin<br><br>Functionalization:<br>HA			%EE<br>98.22 ± 2.06%<br><br>Stability:<br>Not informed	and antitumour efficiency	✓ Enhanced antitumoral activity<br>✓ Low (hematologic) side effects		
Etoposide (ETP)	Human gastric cancer (**SGC7901**) cell line<br><br>Mice bearing **SGC7901** cells xenografts<br><br>Intravenous	(Synthetic lipids)<br><br>Solid lipid:<br>GMS<br><br>Liquid lipid:<br>OA<br>SPC<br><br>Surfactant:<br>DOTAP<br>Lecithin	Solvent injection (or solvent displacement)	DLS	Size<br>91.3 ± 2.8 nm<br><br>PDI<br>0.12 ± 0.03<br><br>ZP<br>23.1 ± 3.3 mV<br><br>%EE<br>78.4 ± 3.6%<br><br>Stability<br>Not informed	Pharm. Development<br><br>*In vitro* tests<br><br>*In vivo:* antitumour effects	✓ Improved cytotoxicity<br>✓ Improved anti-tumour efficacy	[68]	[87]
ETP	Acute myelogenous leukaemia (**K562**) cell line	(Synthetic lipids)<br><br>Solid lipid:<br>Cetyl palmitate<br><br>Liquid lipid:<br>Octyldodecanol<br><br>Surfactant:<br>P-188<br>Lecithin<br><br>Functionalization:<br>Tf	Emulsification-solvent diffusion	DLS<br>FTIR	Size<br>171.0 ± 1.4 nm<br><br>PDI<br>0.287 ± 0.043<br><br>ZP<br>23.3± 0.6 mV<br><br>%EE<br>74.07 ± 0.78%<br><br>Stability:<br>Not informed	Pharm. Development<br><br>*In vitro* tests	✓ Improved cytotoxicity	[69]	-

ETP	Human Burkitt's lymphoma (**Raji**) cell line<br><br>Lymphoma model in mice<br><br>Intravenous	(Synthetic lipids)<br><br>Solid lipid:<br>GMS<br><br>Liquid lipid:<br>Miglyol 812N<br><br>Surfactant<br>CTAB<br><br>Functionalization:<br>HA<br>TAT<br>TS	Solvent injection (or solvent displacement)	DLS<br>Microscopy (TEM)<br>FTIR<br>NMR	Size<br>125.2 ± 7.1 nm<br><br>PDI<br>0.27 ± 0.07<br><br>ZP<br>21.3 ± 3.4%<br><br>%EE<br>90.74 ± 3.08%<br><br>Stability:<br>Not informed	Pharm. Development<br><br>*In vitro* tests<br><br>*In vivo:*<br>Tissue biodistribution<br>Antitumour efficacy	✓ Improved cytotoxicity and cellular uptake<br>✓ Improved antitumour efficacy with low side-effects	[70]	-
ETP	**A549** cells	(Natural lipids)<br><br>Solid lipid:<br>GMS<br><br>Liquid lipid:<br>Soybean oil<br><br>Surfactant:<br>Lecithin<br><br>Functionalization:<br>DSPE-PE or PEG-40-st	Emulsification-solvent evaporation	PCS<br>Microscopy (TEM)	Size<br>125.9–91.2 nm<br><br>PDI<br>< 0.2<br><br>ZP<br>28.49 to 15.34 mV<br><br>%EE<br>57.9 – 89.7%<br><br>Stability:<br>Not informed	Pharm. Development<br><br>*In vitro* tests<br><br>*In vivo:*<br>Pharmacokinetic study in rats and Intestinal absorption	✓ Improved cytotoxicity<br>✓ Superior absorption by the guts<br>✓ Improved bioavailability in rats after oral administration	[71]	[86]
ETP	**CT26**, **SGC7901** and **NCI-H209** cells<br><br>Gastric	(Synthetic lipids)<br><br>Solid lipid:<br>GMS<br><br>Liquid lipid:<br>OA	Solvent injection (or solvent displacement)	PCS<br>Microscopy (TEM)	Size<br>120.91 ± 3.59 nm<br><br>PDI<br>0.139 ± 0.026<br><br>ZP	Pharm. Development<br><br>*In vitro* tests<br><br>*In vivo:*	✓ Selective delivery to human gastric cancer cells<br>✓ Enhancement of the antitumour capacity	[72]	[88]

	tumour-bearing mice (**SGC7901** cells)<br><br>Intravenous	Surfactant:<br>DOTAP<br>Lecithin<br><br>Functionalization:<br>FA-PEG-DSPE			21.3 mV<br><br>%EE<br>83.86 ± 3.21%<br><br>Stability:<br>24 h	Tissues distribution assay and antitumour effects	✓		
Topotecan (TPT)	**B16F10** cells	(Synthetic lipids)<br><br>Solid lipid:<br>SA<br><br>Liquid lipid:<br>OA<br><br>Surfactant:<br>Lecithin<br>Taurodeoxycholate	Microemulsion technique	DLS<br>NTA<br>Microscopy (AFM)	Size<br>183.2 nm<br><br>PDI<br>0.3<br><br>ZP<br>75.9 mV<br><br>%EE<br>Not informed<br><br>Stability:<br>Not informed	Pharm. Development<br><br>*In vitro* tests<br>Skin permeation	✓ Enhanced cytotoxicity	[73]	-

Codelivery of conventional AA by NLC

**Drug**	**Tumour tested and route**	**Composition**	**Preparation method**	**Characterization**	**Physical-chemical properties**	**Tests conducted**	**Main results**	**Ref.**	**Ref. in the review**
DOX<br><br>+<br><br>PTX	**A549** cells<br><br>Pulmonary carcinoma<br><br>Nasal	(Synthetic lipids)<br><br>Solid lipid:<br>SA<br>GMS<br>Soya lecithin	Emulsification-solvent evaporation	DNPA<br>Microscopy (SEM)<br>DSC<br>XRD	Size<br>394.1 ± 5.6 nm<br><br>PDI<br>0.180 ± 0.02	Pharm. Development<br><br>*In vitro* tests<br><br>*In vivo:*	✓ Enhanced retention and drug accumulation in the lung without signs	[74]	[20]

		Liquid lipid:<br>OA<br><br>Surfactant<br>Cremophor EL			ZP<br>-18.17 ± 2.3 mV<br><br>%EE<br>79.87 ± 3.32%<br><br>Stability<br>Not informed	Lung deposition, bio-distribution (lungs, liver, kidney, and spleen) and lungs histology	of tissue abnormality		
DOX<br><br>+<br><br>PTX	**NCL-H460** cells<br><br>Mice models grafting **NCL-H460** cells<br><br>Intravenous	(Synthetic lipids)<br><br>Solid lipid:<br>ATO<br><br>Liquid lipid:<br>OA<br><br>Surfactant<br>DOTMA	Sonication or ultra-sonication	DLS	Size<br>129.3 ± 4.2 nm<br><br>PDI<br>0.18 ± 0.05<br><br>ZP<br>26.6 ± 3.2 mV<br><br>% EE_{DOX}<br>83.7 ± 4.1<br><br>% EE_{PTX}<br>81.9 ± 4.4<br><br>Stability<br>Not informed	Pharm. Development<br><br>*In vitro* tests<br><br>*In vivo:*<br>Antitumour efficiency	✓ Improved cytotoxicity<br>✓ Improved efficacy<br>✓ Synergistic DTX-PTX effects<br>✓ Lower systemic toxicity	[75]	[92]
DOX<br><br>/<br><br>PTX<br><br>*	**MCF-7**, **SKOV3** cells and their multidrug resistant (**MCF-7/ADR** and **SKOV3-TR30**) cells	(Synthetic lipids)<br><br>Solid lipid:<br>GMS<br><br>Liquid lipid:<br>OA<br><br>Surfactant:<br>P-188	Emulsification-solvent diffusion	DLS	Size<br>306.3 ± 40.1 nm<br><br>PDI<br>0.325 ± 0.022<br><br>ZP<br>-32.1 ± 2.5 mV<br><br>%EE<br>67.05 ± 2.60%	Pharm. Development<br><br>*In vitro* tests	✓ Improved cytotoxicity and cellular uptake	[76]	-

		Functionalization:<br>FA–SA			Stability<br>Not informed				
DOX<br>+<br>PTX	**MCF-7/ADR** cells	(Synthetic lipids)<br><br>Solid lipid:<br>SA<br><br>Liquid lipid:<br>SPC<br><br>Surfactant:<br>CTAB<br><br>Functionalization:<br>FA	Emulsification-solvent evaporation	DLS<br>Microscopy (TEM)<br>DSC<br>FTIR	Size<br>196 ± 2.5 nm<br><br>PDI<br>0.214 ± 0.04<br><br>ZP<br>23.4 ± 0.3 mV<br><br>$\%EE_{DOX}$<br>89.6 ± 0.5<br><br>$\%EE_{PTX}$<br>88.3 ± 0.2<br><br>Stability<br>Not informed	Pharm. Development<br><br>*In vitro* tests	✓ Improved cytotoxicity and synergistic effect of drug combination	[77]	-
DOX<br>+<br>PTX	Glioma stem cells (CD133+) **U87** cells<br><br>Intravenous	(Synthetic lipids)<br><br>Solid lipid:<br>ATO<br><br>Liquid lipid:<br>OA<br>SPC<br><br>Surfactant:<br>DOTMA<br>Lecithin<br><br>.	Sonication or ultra-sonication	DLS<br>Microscopy (TEM)	Size<br>122.83 ± 1.97 nm<br><br>PDI<br>0.31 ± 0.04<br><br>ZP<br>18.64 ± 1.46 mV<br><br>$\%EE_{DOX}$<br>66.45 ± 4.02<br><br>$\%EE_{PTX}$<br>62.96 ± 3.38	Pharm. Development<br><br>*In vitro* tests<br><br>*In vivo:* tumour progression; Immunohistochemistry; Biochemical analysis.	✓ Inhibitory effect on colony formation<br>✓ Suppression of proliferation and induced apoptosis by blocking the PI3K/Akt/mTOR signalling	[78]	[93]

					Stability<br>Not informed				
DOX<br><br>+<br><br>CIS	**MCF-7** and **MCF-7/ADR cells**<br><br>MCF-7/ADR tumour-bearing mice models<br><br>Intravenous	(Synthetic lipids)<br><br>Solid lipid:<br>SA<br><br>Liquid lipid<br>SPC<br><br>Surfactant:<br>DDAB	Emulsification-solvent evaporation	PCS	Size<br>108.3 ± 2.3 nm<br><br>PDI<br>Not informed<br><br>ZP<br>16.4 ± 1.6 mV<br><br>%EE_{DOX}<br>89.5 ± 3.7%<br><br>%EE_{CIS}<br>86.7 ± 2.8%<br><br>Stability<br>Not informed	Pharm. Development<br><br>*In vitro* tests<br><br>*In vivo:*<br>Anti-tumour efficacy	✓ Improved cytotoxicity<br>✓ Efficient on DOX-resistant MCF-7 cells<br>✓ Improved tumour concentration than in the heart and kidney<br>✓ Higher tumour inhibition	[79]	[94]
DOX<br><br>+<br><br>VCR	Diffuse large B-cell lymphoma (DLBCL) Cell lines (**LY1**)<br><br>**LY1** cells in mice<br><br>Intravenous	(Synthetic lipids)<br><br>Solid lipid:<br>ATO<br><br>Liquid lipid:<br>Cremophor ELP<br><br>Surfactant:<br>DDBA	Microemulsion technique	DLS	Size<br>95.8 ± 2.1 nm<br><br>PDI<br>Not informed<br><br>ZP<br>21.3 ± 2.8 mV<br><br>%EE_{DOX}<br>87.9 ± 2.4<br><br>%EE_{VCR}<br>83.3 ± 2.1<br><br>Stability<br>Not informed	Pharm. Development<br><br>*In vitro* tests<br><br>*In vivo:*<br>*Animal*<br>Antitumour efficacy	✓ High anti-tumour activity against lymphoma cells<br>✓ DOX-VCR synergistic effect	[80]	[95]

DOX<br><br>+<br><br>GEM<br><br>+<br><br>VCR	**Raji** cells<br><br>**Raji** cells in mouse<br><br>Intravenous	(Synthetic lipids)<br><br>Solid lipid:<br>Precirol ATO 5<br><br>Liquid lipid:<br>PVA<br><br>Surfactant:<br>Lecithin	Solvent injection (or solvent displacement)	DLS<br>Microscopy (TEM)	Size<br>112 ± 5.7 nm<br><br>PDI<br>0.187 ± 0.051<br><br>ZP<br>−39.7 ± 4.1 mV<br><br>$\%EE_{DOX}$<br>86.1 ± 2.7%<br><br>$\%EE_{GEM}$<br>86.8 ± 3.1%<br><br>$\%EE_{VCR}$<br>89.2 ± 2.9%<br><br>Stability<br>Not informed	Pharm. Development<br><br>*In vitro* tests<br><br>*In vivo:*<br>Efficacy, biodistribution	✓ Enhanced cytotoxicity<br>✓ Low drug concentration to heart and kidney<br>✓ Better antitumour therapeutic efficiency	[81]	[96]
DOX<br><br>+<br><br>Baicalein (BCL)	**MCF-7/ADR** cell<br><br>**MCF-7/ADR** tumour-bearing mice<br><br>Intravenous	(Synthetic lipids)<br><br>Solid lipid:<br>SA<br>Precirol ATO 5<br>SPC<br><br>Liquid lipid:<br>Cremophor ELP<br><br>Surfactant:<br>Lecithin<br>DDAB<br><br>Functionalization:<br>HA	Emulsification-solvent evaporation	DLS	Size<br>103.5 ± 2.2 nm<br><br>PDI<br>Not informed<br><br>ZP<br>12.6 ± 1.2 mV<br><br>$\%EE_{BCL}$<br>90.8 ± 1.7%<br><br>$\%EE_{DOX}$<br>91.5 ± 1.8%<br><br>Stability:<br>Not informed	Pharm. Development<br><br>*In vitro* tests<br><br>*In vivo:*<br>anti-tumour efficacy	✓ Improved cytotoxicity with synergistic effect<br>✓ Improved antitumour efficacy with reduced systemic toxicity	[82]	[97]

DOX<br><br>+<br><br>Sclareol<br>(SC)	**MDA-MB-231** and **4T1** cells<br><br>**4T1** tumour-bearing mice<br><br>Intravenous	(Natural lipids)<br><br>Solid lipid:<br>ATO<br><br>Liquid lipid:<br>OA<br>Peanut oil<br><br>Surfactant:<br>Tween 80	Sonication or ultra-sonication	DLS<br>Microscopy (PLM)<br>DSC<br>SAXS	Size<br>104 ± 12 nm<br><br>PDI<br>0.23 ± 0.02<br><br>ZP<br>-31 ± 2<br><br>%EE<br>97 ± 2%<br><br>Stability:<br>Not informed	Pharm. Development<br><br>*In vitro* tests<br><br>*In vivo:*<br>Antitumour activity	✓ Improved cytotoxicity DOX-SC synergism<br>✓ Improved antitumoral efficiency	[83]	[98]
DOX<br><br>+<br><br>β-elemene	**A549** and **A549/ADR** cells<br><br>Tumour xenografts **A549/ADR** in mice<br><br>Intravenous	(Synthetic lipids)<br><br>Solid lipid:<br>ATO<br><br>Liquid lipid:<br>Miglyol 812<br><br>Surfactant:<br>Lecithin<br>Tween 80<br><br>Functionalization:<br>mPEG-Hyd-DSPE	Sonication or ultra-sonication	DLS<br>Microscopy (TEM)	Size<br>190 nm<br><br>PDI<br>0.2<br><br>ZP<br>−30.9 and −41.3 mV<br><br>$\%EE_{DOX}$<br>89.3 ± 3.9%<br><br>$\%EE_{ELE}$<br>87.7 ± 4.2%<br><br>Stability:<br>72h	Pharm. Development<br><br>*In vitro* tests<br><br>*In vivo:*<br>Antitumour activity	✓ Improved cytotoxicity DOX-β-elemene synergistic effect<br>✓ Improved antitumoral efficiency	[84]	[99]
DOX<br><br>+	**MCF-7/ADR** cells	(Synthetic lipids)<br><br>Solid lipid:	Sonication or ultra-sonication	DLS<br>Microscopy (CLSM / TEM)	Size<br>100.2 ± 6.8 nm	Pharm. Development	✓ Improved DOX retention in tumour	[85]	[100]

β-Lapachone (LAPA)	**MCF-7/ADR** tumour-bearing mouse<br><br>Intravenous	GMS<br>ATO<br><br>Liquid lipid:<br>OA<br>SPC<br><br>Surfactant<br>P-188<br>PEG-SA			PDI<br>0.123<br><br>ZP<br>-23.5 ± 3.6 mV<br><br>%EE<br>92.3%<br><br>Stability:<br>Not informed	*In vitro* tests<br><br>*In vivo:*<br>anticancer efficiency and distribution	✓ Enhanced tumour inhibition		
PTX<br><br>+<br><br>CIS	Head and neck cancer (**FaDu**) cell line<br><br>**FaDu** in mice<br><br>Intravenous	(Natural lipids)<br><br>Solid lipid:<br>ATO<br><br>Liquid lipid:<br>Olive oil<br>Cremophor ELP<br><br>Surfactant:<br>CTAB<br>Tween 80<br><br>Functionalization:<br>FA-PEG-DSPE	Solvent injection (or solvent displacement)	PCS<br>Microscopy (TEM)	Size<br>127.1 ± 5.1 nm<br><br>PDI<br>0.23 ± 0.07<br><br>ZP<br>26.7 ± 2.2 mV<br><br>$\%EE_{CIS}$<br>82.1 ± 3.4%<br><br>$\%EE_{PTX}$<br>79.2 ± 3.1%<br><br>Stability<br>Not informed	Pharm. Development<br><br>*In vitro* tests<br><br>*In vivo:*<br>Tissue distribution and Anticancer effect	✓ Improved cytotoxicity<br>✓ Improved tumour regressions	[86]	[101]
PTX<br><br>+	**A549** cells<br><br>Xenografts **A549** cells in mice<br><br>Intravenous	(Synthetic lipids)<br><br>Solid lipid:<br>ATO<br><br>Liquid lipid	Emulsification-solvent diffusion	DLS<br>Microscopy (TEM)	Size<br>131.6 ± 3.5 nm<br><br>PDI<br>0.131 ± 0.026	Pharm. Development<br><br>*In vitro* tests<br><br>*In vivo:*	✓ Improved cell uptake and the combination treatment<br>✓ PTX-DMN synergistically	[87]	[102]

5-Demethylnobiletin (DMN)		OA<br>SPC<br><br>Surfactant:<br>Lecithin<br>DSPE-PEG-Mal<br>DDAB			ZP<br>19.5 ± 2.7 mV<br><br>%EE_{PTX}<br>90.5 ± 4.2%<br><br>%EE_{DMN}<br>91.1 ± 3.7%<br><br>Stability:<br>90 days	Anti-tumour effects and tissue distribution in mice	decreased cancer cells viability<br>✓ Improved tumour inhibition efficiency		
CIS<br><br>+<br><br>5-FU	Human gastric cancer (**BGC823**) cell line<br><br>Mice bearing **BGC823** human GC xenografts<br><br>Intravenous	(Natural lipids)<br><br>Solid lipid:<br>GMS<br><br>Liquid lipid:<br>soybean oil<br><br>Surfactant:<br>Tween 80<br>Lecithin<br>DDBA<br><br>Functionalization:<br>HA and 5-FU linked to SA	Microemulsion technique	DLS<br>Microscopy (TEM)	Size<br>181.6 ± 3.2 nm<br><br>PDI<br>0.21 ± 0.04<br><br>ZP<br>26.3 ± 2.4 mV<br><br>%$EE_{5\text{-}FU}$<br>89.8 ± 2.9%<br><br>%EE_{CIS}<br>89.1 ± 2.1%<br><br>Stability<br>Not informed	Pharm. Development<br><br>*In vitro* tests<br><br>*In vivo:*<br>Efficacy	✓ Strongest antitumour activity	[88]	[104]
TMZ<br><br>+<br><br>VCR	**U87 MG** cells<br><br>**U87 MG** cells in mice<br><br>Intravenous	(Synthetic lipids)<br><br>Solid lipid:<br>ATO<br><br>Liquid lipid:	Emulsification-solvent diffusion	DLS	Size<br>117.4 ± 2.8 nm<br><br>PDI<br>0.09 ± 0.02<br><br>ZP<br>29.8 ± 3.2 mV	Pharm. Development<br><br>*In vitro* tests<br><br>*In vivo:*<br>Anti-tumour efficacy	✓ Improved cytotoxicity<br>✓ Improved tumour inhibition	[89]	[105]

		Cremophor ELP<br>SPC<br><br>Surfactant:<br>DDAB<br>Lecithin			%EE_{TMZ}<br>88.9 ± 3.6%<br><br>%%EE_{VCR}<br>85.4 ± 2.8%<br><br>Stability<br>Not informed				
TMZ<br><br>+<br><br>VCR	**U87 MG**, human malignant glioma (**T98G**) cell line, and **A549** cells<br><br>**U87 MG** in mice<br><br>Intravenous	(Synthetic lipids)<br><br>Solid lipid:<br>Compritol 888 ATO<br><br>Liquid lipid:<br>Cremophor ELP<br><br>Surfactant:<br>DDAB<br>Lecithin<br><br>Functionalization:<br>Lactoferrin-PEG-DSPE<br>RGD-PEG-DSPE	Emulsification-solvent diffusion	DLS	Size<br>139.3±4.9 nm<br><br>PDI<br>0.187 ± 0.021<br><br>ZP<br>32.4±2.7mV<br><br>%EE_{TMZ}<br>81.9 ± 3.4%<br><br>%%EE_{VCR}<br>82.2 ± 3.2%<br><br>Stability<br>3 months	Pharm. Development<br><br>*In vitro* tests<br><br>*In vivo:*<br>Tissue distribution, antitumour activity	✓ Improved cytotoxicity with TMZ-VCR synergism<br>✓ increased drug accumulation in the tumour<br>✓ improved tumour inhibition with low systemic toxicity	[90]	-
GEM<br><br>+<br><br>PTX	Human pulmonary adenocarcinoma (**A549, LTEPa2**) cell lines<br><br>Tumour xenograft model of **A549** cells in mouse	(Synthetic lipids)<br><br>Solid lipid:<br>GMS<br><br>Liquid lipid:<br>Soy lecithin<br><br>Surfactant:<br>Sodium deoxycholate	Emulsification-solvent evaporation	DLS<br>Microscopy (CLSM / TEM)	Size<br>119.6 ± 0.3 nm<br><br>PDI<br>0.251 ± 0.03<br><br>ZP<br>-20.7 ± .9 mV	Pharm. Development<br><br>*In vitro* tests<br><br>*In vivo:*<br>Antitumour effect	✓ Suppression of tumour growth	[91]	-

	Intravenous	Poloxamer 188<br>Tween 80<br><br>Functionalization:<br>NAG (N-acetyl-d-glucosamine), a Glucose receptor ligand.			$\%EE_{GEM}$<br>83.7 ± 4.15<br><br>$\%EE_{PTX}$<br>83.5 ± 2.72<br>Stability:<br>Not informed				
GEM<br><br>+<br><br>PTX<br><br>(Covalently bound through an ester linker form drug conjugates) associated to NAG-NLC	Cell lines: **A549, NCLH1299** and **LTEPa2**	(Synthetic lipids)<br><br>Solid lipid:<br>GMS<br><br>Liquid lipid:<br>Soy lecithin<br><br>Surfactant:<br>P-188<br>Tween 80<br><br>Functionalization:<br>NAG	Emulsification-solvent evaporation	DLS<br>Microscopy (CLSM / TEM)	Size<br>120.3 ± 1.3 nm<br><br>PDI<br>0.233 ± 0.04<br><br>ZP<br>-27.8 ± 3.4 mV<br><br>%EE<br>Not informed<br><br>Stability<br>Not informed	Pharm. Development<br><br>*In vitro* tests	✓ Superior cytotoxicity<br>✓ High uptake by A549 cells (via glucose receptor-mediated endocytosis)	[92]	-
GEM<br><br>+<br><br>BCL	Human pancreatic adenocarcinoma (**AsPC1**) cell line and **HeLa** cells<br><br>AsPC1 cells in mice	(Synthetic / Natural lipids)<br><br>Solid Lipid:<br>Precirol ATO-5<br><br>Liquid lipid:	Solvent evaporation	PCS<br>H NMR and IR spectroscopy	Size<br>131.9 ± 3.9 nm<br><br>PDI<br>0.12 ± 0.03	Pharm. Development<br><br>*In vitro* tests<br><br>*In vivo:*	✓ Significant tumour growth inhibition	[93]	-

	Intravenous	Olive oil<br><br>Surfactant:<br>Tween 80<br>DDAB<br>SPC<br><br>Functionalization:<br>HA			ZP<br>-25.9 ± 1.9 mV<br><br>%EE_{GEM}<br>85.1 ± 2.3%<br><br>%EE_{BCL}<br>82.9 ± 2.4%<br><br>Stability<br>1 month	Antitumour effect<br>Tissue distribution			
PTX<br><br>+<br><br>Salinomycin (SAL)	**NCl-H1299** and **S180** cells<br><br>**S180** cells in mice<br><br>Intravenous	(Synthetic lipids)<br><br>Solid lipid:<br>ATO<br><br>Liquid lipid:<br>MCT<br><br>Surfactant<br>Polyoxyethylene (40) stearate<br>Solutol HS15<br>Kolliphor EL<br><br>Functionalization:<br>DSPE-PEG-TR	Microemulsion technique	DLS<br>Microscopy (CLSM)	Size<br>128.73 ± 2.09 nm<br><br>PDI<br>Not informed<br><br>ZP<br>−28.3 ± 0.4 mv<br><br>%EE<br>95.62 ± 1.46%<br><br>Stability<br>Not informed	Pharm. Development<br><br>*In vitro* tests<br><br>*In vivo:*<br>Antitumour effect	✓ Greater tumour suppression	[94]	-
PTX<br><br>+<br><br>SiRNA	**A549** cells<br><br>Mouse orthotopic model of human lung cancer (**A549**)<br><br>Inhalation	(Synthetic lipids)<br><br>Solid lipid:<br>Precirol ATO 5<br><br>Liquid lipid:<br>Squalene<br>SPC	Sonication or ultra-sonication	DLS<br>Microscopy (AFM)	Size<br>110 ± 20 nm<br><br>PDI<br>0.4<br><br>ZP<br>60.3 mV	Pharm. Development<br><br>*In vitro* tests<br><br>*In vivo:*<br>Efficacy, biodistribution	✓ Improved accumulation in lung cancer<br>✓ Enhanced antitumour efficiency	[95]	-

		Surfactant<br>Tween-80<br>DOTAP<br>Lecithin<br><br>Functionalization:<br>DSPE-PEG-LHRH			%EE<br>Not informed<br><br>Stability<br>1 month				
PTX<br><br>+<br><br>pEGFP	**NCl-H460** cells<br><br>**NCl-H460** cells in mice<br><br>Intravenous	(Synthetic lipids)<br><br>Solid lipid:<br>GMS<br><br>Liquid lipid<br>OA<br><br>Surfactant:<br>DOTMA<br>Tween-80<br>Lecithin<br><br>Functionalization:<br>Tf-PEG-PE	Microemulsion technique	LLS<br>Microscopy (TEM)	Size<br>133 nm<br><br>PDI<br>Not informed<br><br>ZP<br>19 mV<br><br>%EE<br>85.4 ± 2.9%<br><br>Stability<br>Not informed	Pharm. Development<br><br>*In vitro* tests<br><br>*In vivo:*<br>Antitumour efficacy; gene transfection analysis.	✓ Increased *in vivo* gene transfection efficiency<br>✓ Improved antitumour efficacy	[96]	-
DOX<br><br>+<br><br>Sorafenib (SFN)	Mice drug-resistant oesophagus carcinoma (**AKR/Dox**) cell line<br><br>AKR/Dox tumour-bearing model in mice.<br><br>Intravenous	(Synthetic lipids)<br><br>Solid lipid:<br>GMS<br><br>Liquid lipid<br>OA<br><br>Surfactant:<br>Lecithin<br>PC<br><br>Functionalization:<br>SA-PEG-FA	Emulsification-solvent diffusion	DLS	Size<br>100 nm<br><br>PDI<br>Not informed<br><br>ZP<br>Not informed<br><br>%EE<br>Not informed<br><br>Stability<br>Not informed	Pharm. Development<br><br>*In vitro* tests<br><br>*In vivo:*<br>anticancer efficacy, immune *activation*	✓ DOX-SFN synergistic effect (improved cytotoxicity)<br><br>✓ Strong immune response after treatment	[97]	-

MTX<br>+<br>DTX<br>+<br>DOX	**MCF-7** and **A549** cells	(Synthetic lipids)<br><br>Solid lipid:<br>GMS<br>SA<br><br>Liquid lipid:<br>Miglyol 812<br><br>Surfactant:<br>Poloxamer 407	Solvent injection (or solvent displacement)	DLS<br>Microscopy (SEM / TEM)<br>FTIR	Size<br>233.3 nm<br><br>PDI<br>0.14<br><br>ZP<br>Not informed<br><br>%EE_{MTX}<br>33.73%<br><br>%EE_{DOX}<br>90.48%<br><br>%EE_{DTX}<br>98.95%<br><br>Stability<br>Not informed	Pharm. Development<br><br>*In vitro* tests	✓ Increased cell uptake, apoptosis, autophagy, ROS levels<br>✓ Clonogenicity tests revealed decreased colony formation in MCF-7 cells	[98]	-
DTX<br>+<br>Trastuzumab	Breast cancer **(MDA-MB-468 and BT-474)** cell lines	(Natural lipids)<br><br>Solid lipid:<br>SA<br>Stearyl amine<br>Spermine<br>Chol<br><br>Liquid lipid:<br>Castor oil<br><br>Surfactant<br>Poloxamer F127	Sonication or ultra-sonication	DLS<br>Microscopy (SEM/ TEM)	Size<br>199 ± 10 nm<br><br>PDI<br>0.65 ± 0.05<br><br>ZP<br>~18 mV<br><br>%EE_{DTX}<br>75.5 ± 2.0%<br><br>Stability<br>Not informed	Pharm. Development<br><br>*In vitro* tests	✓ Enhanced cellular uptake<br>✓ Higher cytotoxic effect	[99]	-
DTX	**MDA-MBA-231** cells	(Synthetic lipids)	Sonication or ultra-sonication	DLS<br>Microscopy (SEM)	Size<br>55 a 110 nm	Pharm. development	✓ Improved cytotoxicity	[100]	-

+<br><br>Myricetin		Solid lipid:<br>ATO<br><br>Liquid lipid:<br>Miglyol 812<br><br>Surfactant:<br>Poloxamer 407<br><br>Functionalization:<br>Myricetin			PDI<br>0.19<br><br>ZP<br>-12 mV<br><br>% EE<br>Not informed<br><br>Stability<br>Not informed	*In vitro* tests	✓ Increase in apoptotic cells		

New approaches in chemotherapy

Curcumin as non-conventional AA encapsulated in NLC Curcumin

Drug	Tumour tested and route	Composition	Preparation method	Characterization	Physical-chemical properties	Tests conducted	Main results	Ref.	Ref. in the review
Curcumin (CUR)	Human epithelial ovarian cancer (**A2780S** and **A2780CP**) cell lines	(Synthetic lipids)<br><br>Solid lipid:<br>ATO<br><br>Liquid lipid:<br>Captex 355 EP/NF<br>Miglyol 812<br><br>Surfactant:<br>SPC<br>Taurocholate	Microemulsion technique	DLS<br>Microscopy (fluorescent)<br>FTIR	Size<br>~100 nm<br><br>PDI<br>> 0.2<br><br>ZP<br>−30 mV<br><br>%EE<br>40%<br><br>Stability	Development<br><br>*In vitro* tests	✓ Improved cytotoxicity<br>✓ Improved inhibition colony formation<br>✓ No haemolytic effect	[101]	-

					Not informed				
CUR	Human neuroblastoma cell line (**LAN5**)	(Synthetic lipids)<br><br>Solid lipid:<br>ATO<br>Precirol ATO<br><br>Liquid lipid:<br>Imwitor 800<br><br>Surfactant:<br>Lecithin<br>Taurocholate	Microemulsion technique	PCS<br>Microscopy (fluorescent)	Size<br>110 − 135 nm<br><br>PDI<br>0.2 – 0.3<br><br>ZP<br>−24 to − 18 mV<br><br>%EE<br>Not informed<br><br>Stability<br>Not informed	Development<br><br>*In vitro* tests	✓ Improved cytotoxicity	[102]	-
CUR	**A549** cells	(Synthetic lipids)<br><br>Solid lipid:<br>Monostearin<br><br>Liquid lipid:<br>Octyl decyl acid triglycerate<br><br>Surfactant:<br>Lecithin<br>P-188<br>Tween-80	Microemulsion technique	DLS<br>Microscopy (fluorescent)	Size<br>99.99 nm<br><br>PDI<br>0.158<br><br>ZP<br>−19.9 mV<br><br>%EE<br>97.86 %<br><br>Stability<br>Not informed	Development<br><br>*In vitro* tests<br><br>*In vivo* test:<br>Pharmacokinetic	✓ Improved cytotoxicity<br>✓ Increased apoptosis<br>✓ Improved bioavailability	[103]	-
CUR	**HepG2** cells	(Synthetic lipids)<br><br>Solid lipid:<br>Monostearin<br><br>Liquid lipid:	Microemulsion technique	DLS	Size<br>99.99 ± 1.87 nm<br><br>PDI<br>0.158 ± 0.005<br><br>ZP	Development<br><br>*In vitro* tests	✓ Improved cytotoxicity<br>✓ increased caspase-8 and caspase-3 activities<br>✓ increased apoptosis	[104]	-

		octyl decyl acid triglycerate<br><br>Surfactant:<br>Lecithin<br>P-188<br>Tween-80			−19.9 ± 0.65 mV<br><br>%EE<br>97.86 ± 0.72 %<br><br>Stability<br>Not informed				
CUR	**MCF-7 cells**	(Natural lipids)<br><br>Solid lipid:<br>Cetyl palmitate<br><br>Liquid lipid:<br>Cod-liver oil<br><br>Surfactant:<br>Tween 80<br><br>Functionalization:<br>Fe_3O_4 particles	Sonication or ultra-sonication	DLS<br>Microscopy (TEM)	Size<br>166.7 ± 14.20 nm<br><br>PDI<br>0.24 ± 0.14<br><br>ZP<br>-27.6 ± 3.83 mV<br><br>%EE<br>99.95 ± 0.015%<br><br>Stability<br>5 months	Development<br><br>*In vitro* tests	✓ Improved cytotoxicity	[105]	-
CUR	Human brain cancer cell (**A172**)<br><br>Mice bearing **A172** xenografts<br><br>Intraperitoneally	(Synthetic lipids)<br><br>Solid lipid:<br>Tripalmitin<br><br>Liquid lipid:<br>OA<br><br>Surfactant:<br>Tween 80	High-pressure homogenization (HPH)	PCS<br>Microscopy (CLSM /TEM)	Size<br>214.0 nm<br><br>PDI<br>Not informed<br><br>ZP<br>Not informed<br><br>%EE<br>88.6%<br><br>Stability	Development<br><br>*In vitro* tests<br><br>*In vivo* test:<br>Tumour regression, plasma concentration and tissue distribution	✓ Improved cytotoxicity<br>✓ Apoptosis ratio and ROS levels<br>✓ Higher bioavailability<br>✓ Significant tumour inhibition	[106]	[108]

					Not informed				
CUR<br>+<br>PDT	**MCF-7** cells	(Natural lipids)<br><br>Solid lipid:<br>GMO<br><br>Liquid lipid:<br>Olive oil<br><br>Surfactant:<br>Tween 80<br>Lecithin	High-pressure homogenization (HPH)	DLS<br>Microscopy (TEM)	Size<br>113.94 ± 11.3 nm<br><br>PDI<br>0.29 ± 0.05<br><br>ZP<br>−47.50 ± 2.69<br><br>%EE<br>82.49%<br><br>Stability<br>2 months	Pharm. Development<br><br>*In vitro* tests	✓ Superior cytotoxicity in breast cancer cells	[107]	-
CUR<br>+<br>Imatinib (monoclonal antibody – IMT)	Non-Hodgkin lymphoma (**Jurkat** and **Ramos** cell lines)	(Synthetic lipids)<br><br>Solid lipid:<br>GMS<br><br>Liquid lipid:<br>OA<br><br>Surfactant:<br>Tween 80<br>Lecithin<br><br>Functionalization:<br>Rituximab	Emulsification-solvent diffusion	DLS<br>Microscopy (AFM)	Size<br>272.0 ± 0.3 nm<br><br>PDI<br>0.4<br><br>ZP<br>8.2 ± 0.1mV<br><br>%EE<br>100%<br><br>Stability<br>Not informed	Pharm. Development<br><br>*In vitro* tests	✓ CUR-NLC showed significant cytotoxic effect<br>✓ Co-administration with imatinib reduced the imatinib doses.	[108]	[109]
CUR<br>+<br>DTX	**NCL-H460** cells<br><br>Urethane induced lung carcinoma in mice	(Synthetic lipids)<br><br>Solid lipid:<br>Glyceryl Palmitostearate<br>Trimyristin	High-pressure homogenization (HPH)	DLS<br>Microscopy (TEM)<br>DSC<br>FTIR<br>PXRD	Size<br>150.2 ± 5.2 nm<br><br>PDI<br>0.263 ± 0.15	Pharm. Development<br><br>*In vitro* tests<br><br>In vivo:	✓ Improved cytotoxicity<br>✓ Significantly *better in vivo* effects than Taxotere®<br>✓ Lower toxicity	[109]<br>[110]	[110]<br>[91]

	Intravenous	Liquid lipid:<br>MCT<br><br>Surfactant:<br>Solutol HS15<br>Lecithin<br><br>Functionalization:<br>FA			ZP<br>26.3 ± 5.2 mV<br><br>$\%EE_{DTX}$<br>94.4 ± 2.3 %<br><br>$\%EE_{CUR}$<br>95.10 ± 2.8%<br><br>Stability<br>Not informed	Pharmacokinetics studies; Pharmacodynamics study; efficacy, Toxicity			
CUR<br><br>+<br><br>TMZ	Glioma (C6) cells<br><br>C6 in mice<br><br>Intravenous	(Synthetic lipids)<br><br>Solid lipid:<br>GMS<br><br>Liquid lipid:<br>MCT<br><br>Surfactant:<br>P-188	Microemulsion technique	DLS<br>Microscopy (TEM)<br>DSC	Size<br>78.49 ± 0.38 nm<br><br>PDI<br>0.22 ± 0.01<br><br>ZP<br>- 8.54 ± 0.51 mV<br><br>$\%EE_{TMZ}$<br>70.90% ± 0.06%<br><br>$\%EE_{CUR}$<br>68.17% ± 0.20%<br><br>Stability<br>Not informed	Pharm. Development<br><br>*In vitro* tests<br><br>*In vivo:*<br>Antitumour effect, biodistribution and toxicity in mice	✓ Significant synergistic (CUR/TMZ) anticancer effect *in vivo*.<br>✓ No toxic effects of on major organs and normal cells at the same therapeutic dose.	[111]	[111]

Photodynamic and photothermal therapy

Photosensitizer	Tumour tested and route	Composition	Preparation method	Characterization	Physical-chemical properties	Tests conducted	Main results	Ref.	Ref. in the review

Chloroaluminum phthalocyanine (ClAlPc)	**A549** and **B16-F10** cells	(Synthetic lipids)<br><br>Solid lipid:<br>SA<br><br>Liquid lipid:<br>OA<br><br>Surfactant:<br>Sodium lauryl sulfate	Emulsification-solvent diffusion	DLS<br>Microscopy (TEM)<br>DSC<br>FTIR<br>SAXS	Size<br>205.9 ± 22.7 nm<br><br>PDI<br>0.46 ± 0.04<br><br>ZP<br>−69.1 ± 18.5 mV<br><br>%EE<br>95.8 ± 0.03 %<br><br>Stability<br>3 months	Pharm. Development<br><br>*In vitro* tests<br><br>In vivo:<br>Skin permeation in rat	✓ Improved cytotoxicity<br>✓ Increased the retained of drug into the skin.<br>✓ biocompatible	[112]	[123]
IR780	Human epithelial colorectal adenocarcinoma cell line (**Caco-2**) and mouse colon cancer cell line (**CT-26**)<br><br>**CT-26** in mice<br><br>Oral	(Synthetic lipids)<br><br>Solid lipid:<br>Trilaurin<br><br>Liquid lipid:<br>CT<br><br>Surfactant:<br>Soy lecithin	Emulsification-solvent evaporation	DLS<br>Microscopy (TEM / CLSM)	Size<br>170 nm<br><br>PDI<br>0.057<br><br>ZP<br>Not informed<br><br>%EE<br>Not informed<br><br>Stability<br>2h	Pharm. Development<br><br>*In vitro* tests<br><br>*In vivo:*<br>*Pharmacokinetics, Biodistribution, and Therapeutic Activity*	✓ Enhanced oral absorption<br>✓ Increased oral bioavailability<br>✓ Improved antitumour efficacy	[113]	[121]
IR780	**4T1** cells<br><br>**4T1** in mice<br><br>Intravenous	(Synthetic lipids)<br><br>Solid lipid:<br>Trilaurin<br><br>Liquid lipid:	Emulsification-solvent evaporation	DLS<br>Microscopy (TEM / CLSM)	Size<br>~135 nm<br><br>PDI<br>Not informed	Pharm. Development<br><br>*In vitro* tests<br><br>*In vivo:*	✓ Photothermal properties of IR780 were improved<br>✓ Accumulated at high levels in tumours	[114]	[122]

		MCT<br><br>Surfactant:<br>PC<br><br>Functionalization:<br>AMD3100			ZP<br>−12 mV<br><br>%EE<br>94.6 ± 1.4%<br><br>Stability<br>Not informed	Pharmacokinetics, Biodistribution, and Therapeutic Activity	✓ Anti-metastatic efficacy		
Verteporfin (VTP)	Human ovarian cancer cell lines: **SKOV3**, **OVCAR3** and **IGROV1**<br><br>**SKOV3** in mice<br><br>Intravenous	(Natural lipids)<br><br>Solid lipid:<br>Suppocir NB<br><br>Liquid lipid:<br>Soybean oil<br><br>Surfactant:<br>Polyoxyethylene 40	Emulsification-solvent evaporation	DLS<br>Microscopy (CLSM)	Size<br>~47.9 ± 1.0<br><br>PDI<br>0.12 ± 0.02<br><br>ZP<br>−3.7 ± 0.9<br><br>%EE<br>< 95 %<br><br>Stability:<br>Not informed	Pharm. Development<br><br>*In vitro* tests<br><br>*In vivo:*<br>Pharmacokinetics, Biodistribution, and Therapeutic Activity	✓ Enhanced deleterious effects in ovarian cancer cells cultured in monolayer, in spheroids, and *in vivo* (in tumour bearing mice).	[115]	[124]
Zinc phthalocyanine (ZnPc)	**MCF-7** cells	(Synthetic lipids)<br><br>Solid lipid:<br>Ethoxylated hydrogenated Castor oil 40<br><br>Liquid lipid:<br>capric/caprylic acid triglycerides<br><br>Surfactant:<br>Pluronic F127	Emulsification-solvent evaporation	DLS<br>Microscopy (CLSM)<br>FTIR<br>NMR	Size<br>166.7 ± 1.33 nm<br><br>PDI<br>0.14 ± 0.018<br><br>ZP<br>−16.4 ± 0.45 mV<br><br>%EE<br>63.00 ± 1.19%<br><br>Stability<br>3 months	Pharm. Development<br><br>*In vitro* tests	✓ New nanocarrier for photodynamic therapy, rationally designed<br>✓ The photosensitizer was by first time used in drug delivery tests and MTT assays.	[116]	[120]

		Polyoxyethylene 40<br><br>Functionalization:<br>FA							
5-amino levulinic acid<br>(5-ALA)	Vero E6 cell lines	(Synthetic lipids)<br><br>Solid lipid:<br>Compritol ATO 888<br><br>Liquid lipid:<br>OA<br><br>Surfactant:<br>Tween 20	Microemulsion technique<br>Emulsification-solvent diffusion	DLS<br>Microscopy (CLSM / TEM)<br>DSC	Size<br>194.16 ± 5.73 nm<br><br>PDI<br>0.24 ± 0.03<br><br>ZP<br>Not informed<br><br>%EE<br>72.84 ± 3.81 %<br><br>Stability<br>Not informed	Pharm. Development<br><br>*In vitro* tests<br><br>*In vivo:*<br>skin permeation studies in rat	✓ Increased concentration in the target skin layers	[117]	[118]
DHX-1	**4T1** cells	(Natural lipids)<br><br>Solid lipid:<br>ATO<br><br>Liquid lipid:<br>Babassu oil<br><br>Surfactant:<br>Kolliphor HS	Emulsification-solvent evaporation	DLS<br>Microscopy (TEM)	Size<br>92.1 ± 0.7nm<br><br>PDI<br>0.215 ± 0.030<br><br>ZP<br>-1.12 ± 0.09 mV<br><br>%EE<br>Not informed<br><br>Stability<br>Not informed	Pharm. Development<br><br>*In vitro* tests	✓ *In vitro* demonstration of photosensitizer activity<br>✓ DDS may serve as a theragnostic agent	[118]	[119]
Chlorin e6<br>(Ce6)<br>+<br>PTX	**MDA-MB-231** cells	(Synthetic lipids)<br><br>Solid lipid:<br>Precirol ATO 5	Emulsification-solvent evaporation	DLS<br>Microscopy (CLSM / TEM)<br>DSC	Size<br>120.79 ± 1.68 nm	Pharm. Development<br><br>*In vitro* tests	✓ Long circulation and tumour targeting<br>✓ PDT combined with chemotherapy	[119]	[125]

	**MDA-MB-231 in mice**<br><br>Intravenous	Liquid lipid:<br>Cremophor RH40<br><br>Surfactant:<br>Cremophor RH40<br><br>Functionalization:<br>DSPE-PEG-Ce6		XRD	PDI<br>0.19 ± 0.01<br><br>ZP<br>−38.8 ± 1.5 mV<br><br>%EE<br>76.67 ± 0.61%<br><br>Stability<br>Not informed	*In vivo:*<br>Biodistribution efficacy	produced synergistic effects		
Indocyanine green (ICG)<br><br>+<br><br>PTX	**HepG2** cells<br><br>**HepG2** tumour-bearing mice<br><br>Intravenous	(Synthetic lipids)<br><br>Solid lipid:<br>GMS<br><br>Liquid lipid:<br>OA<br><br>Surfactant<br>P-188<br><br>Functionalization:<br>SA-PEG-FA	Solvent injection (or solvent displacement)	DLS<br>Microscopy (TEM)	Size<br>92.6 nm<br><br>PDI<br>0.115<br><br>ZP<br>Not informed<br><br>%EE<br>Not informed<br><br>Stability: not informed	Pharm. Development<br><br>*In vitro* tests<br><br>*In vivo:*<br>Biodistribution and image	✓ Increased drug intracellular uptake<br>✓ Increased cytotoxicity in cancer cells (ICG-PTX synergistic effect)<br>✓ Excellent tumour targetability in tumour-bearing mice	[120]	-

***** *In this report (ref. 76) two NLC formulations, one with DOX and the other containing PTX were tested and compared.*

Abbreviations:

5-ALA – 5 amino levulinic acid; 2-DG – stearyl-2-amino-2-deoxyglucose; 5-FU – 5-fluorouacil; AF4 – asymmetric flow field-flow fractionation; AFM – atomic force microscopy; ApoB – apolipoprotein B-100; ATO – Compritol 888; BCL – baicalein; CBZ – cabazitaxel; Ce-6 – chlorin e6; CHEMS – cholesteryl hemisuccinate; Chol – Cholesterol; CIS – cisplatin; ClAlPc – Chloroaluminum phthalocyanine; CLSM – confocal laser scanning microscopy; CT – capric triglyceride; CTAB – cetyltrimethylammonium bromide; CUR – curcumin; CXCR4 - antagonist of the chemokine receptor; CYT – cytarabine; DCB – decitabine; DDAB – Didecyldimethylammonium bromide; DHA – docosahexaenoic acid; DLS – dynamic light scattering; DMN – 5-demethylnobiletin ; DN – docetaxel complexed with nicotinamide; DNPA – delsa nano particle analyser; DOTMA – N-[1-(2,3-dioleyloxy)propyl]-N,N,Ntrimethyl-ammonium chloride; DOX –

doxorubicin; DSC – differential Scanning calorimetry; DSPE-PEG – Distearoyl-phosphatidylethanolamine; DSPE-PEG-Ce6 – distearoyl-phosphatidylethanolamine-chlorin e6; DSPE-PEG-Mal –distearoyl-phosphatidylethanolamine-Mal; DSPE-PEG-NH2 – 1,2-Distearoyl-sn-glycero-3-phosphoethanolamine-N-[amino(polyethylene glycol); DSPE-PEG-TR – distearoyl-phosphatidylethanolamine-targeting peptide TISWPPR; DOTAP – 1,2-Dioleoyl-3-trimethylammoniumpropane; DTX – docetaxel; DZ – dacarbazine; EE – encapsulation efficiency; ELS – electrophoretic light scattering; ETP – etoposide; FA – folate acid; FACS – Fluorescence-Activated Cell Sorting; FA-PEG-DSPE – FA containing polyethylene glycol (PEG)-distearoylphosphatidylethanolamine (DSPE); FA-PEGPCHL – folate-poly(PEG-cyanoacrylate-co-cholesteryl cyanoacrylate); FA-SA – folic acid and stearic acid; Fol-PEG-CHEMS – Folate-polyethyleneglycol-cholesterol hemisuccinate; FTIR – Fourier transform infraRed spectroscopy; GMO – glyceryl monooleate; GMS – glyceryl monostearate; HA – hyaluronic acid; ICG – indocyanine green ; IFOS – ifosfamide; IMT – imatinib; Ir – irinotecan; Lapa – β-lapachone; LbL-PEG – layer-by-layer containing polyethylene glycol; LLS – laser light scattering; LPSA – laser particle size analyser; MCT – Medium fatty acids; MCTN – mechlorethamine; MTX – methotrexate; NAG – N-acetyl-d-glucosamine; NLC – nanostructured lipid carriers; NIR – near infrared; NMR – nuclear magnetic resonance spectroscopy; NTA – nanoparticle tracking analysis; OA – oleic acid; OXA – oxaliplatin; P-188 – poloxamer 188; PC – phosphatidylcholine; PCS – photon correlation spectroscopy; PDI – polydispersity index; PDT – photodynamic therapy; PEG – polyethylene glycol; PEG-40-St – Polyethylene glycol 40 stearate; PEG-FA – Polyethylene glycol-Folic acid; pEGFP – green fluorescence protein plasmid; PL – Platelet; PLM – polarized light microscopy; PRXD – powder X-Ray diffraction; PTX – Paclitaxel; PVA – polyvinyl alcohol; RBCm – Red blood cell-membrane; RDG – Arginine-glycine-aspartic acid peptide; RIPL – IPLVVPLRRRRRRRRC peptide; SA- Stearic acid; SAL – salinomycin; SA-PEG-FA – Stearic acid-PEG-folate; SA-R8– Stearyl-polyarginine; SAXS – Small-angle X-ray scattering; SC – sclareol; SiC – sildenafil citrate; SEM – Scanning electronic microscopy; SFN – sorafenib; SPC – soybean phosphatidylcholine; Stattic- STAT3 inhibitor; STEM-EDS – scanning transmission electron microscopy-energy dispersive X-ray spectroscopy; TAT – cell-penetrating peptide transcription activator; TEM – transmission electronic microscopy; Tf – Transferrin; Tf-PEG-PE – transferrin-Polyethylene glycol-phosphatidylethanolamine; TGA – thermogravimetric analysis; TGI – tumour growth inhibition; THP – pirarubicin; TMZ – temozolamide; TPT – topotecan; TS – α-tocopherol succinate; VCR – vincristine sulphate; VTP – verteporfirin; XRD – X-ray diffraction, XPS –X-ray photoelectron spectroscopy; ZnPc – Zinc phthalocyanine; ZP – zeta potential.

References

1. Varshosaz, J.; Hassanzadeh, F.; Sadeghi, H.; Khadem, M. Galactosylated nanostructured lipid carriers for delivery of 5-FU to hepatocellular carcinoma. *J. Liposome Res.* **2012**, *22*, 224–236, doi:10.3109/08982104.2012.662653.
2. Rajinikanth, P.S.; Chellian, J. Development and evaluation of nanostructured lipid carrier-based hydrogel for topical delivery of 5-fluorouracil. *Int. J. Nanomedicine* **2016**, *11*, 5067–5077, doi:10.2147/IJN.S117511.
3. Amasya, G.; Aksu, B.; Badilli, U.; Onay-Besikci, A.; Tarimci, N. QbD guided early pharmaceutical development study: Production of lipid nanoparticles by high pressure homogenization for skin cancer treatment. *Int. J. Pharm.* **2019**, *563*, 110–121, doi:10.1016/j.ijpharm.2019.03.056.
4. Sharma, P.; Dube, B.; Sawant, K. Development and evaluation of nanostructured lipid carriers of cytarabine for treatment of meningeal leukemia. *J. Nanosci. Nanotechnol.* **2011**, *11*, 6676–6682, doi:10.1166/jnn.2011.4235.
5. Neupane, Y.R.; Srivastava, M.; Ahmad, N.; Kumar, N.; Bhatnagar, A.; Kohli, K. Lipid based nanocarrier system for the potential oral delivery of decitabine: Formulation design, characterization, ex vivo, and in vivo assessment. *Int. J. Pharm.* **2014**, *477*, 601–612, doi:10.1016/j.ijpharm.2014.11.001.
6. Abdelbary, G.; Haider, M. In vitro characterization and growth inhibition effect of nanostructured lipid carriers for controlled delivery of methotrexate. *Pharm. Dev. Technol.* **2013**, *18*, 1159–1168, doi:10.3109/10837450.2011.614251.

7. Ferreira, M.; Chaves, L.L.; Lima, S.C.; Reis, S. Optimization of nanostructured lipid carriers loaded with methotrexate : A tool for inflammatory and cancer therapy. *Int. J. Pharm.* **2015**, *492*, 65–72, doi:10.1016/j.ijpharm.2015.07.013.

8. Ong, Y.S.; Bañobre-López, M.; Costa Lima, S.A.; Reis, S. A multifunctional nanomedicine platform for co-delivery of methotrexate and mild hyperthermia towards breast cancer therapy. *Mater. Sci. Eng. C* **2020**, *116*, 111255–111268, doi:10.1016/j.msec.2020.111255.

9. Ferreira, M.; Silva, E.; Barreiros, L.; Segundo, M.A.; Costa Lima, S.A.; Reis, S. Methotrexate loaded lipid nanoparticles for topical management of skin-related diseases: Design, characterization and skin permeation potential. *Int. J. Pharm.* **2016**, *512*, 14–21, doi:10.1016/j.ijpharm.2016.08.008.

10. Chand, P.; Kumar, H.; Badduri, N.; Gupta, N.V.; Bettada, V.G.; Madhunapantula, S.R. V.; Kesharwani, S.S.; Dey, S.; Jain, V. Design and evaluation of cabazitaxel loaded NLCs against breast cancer cell lines. *Colloids Surfaces B Biointerfaces.* **2021**, *199*, 111535–111547, doi:10.1016/j.colsurfb.2020.111535.

11. Lee, S.G.; Kim, C.H.; Lee, E.S.; Goh, M.S; Yoon, H.Y.; Kang, M.J.; Lee, S.; Choi, Y.W. RIPL peptide-conjugated nanostructured lipid carriers for enhanced intracellular drug delivery to hepsin-expressing cancer cells. *Inter. J. Nanomedicine.* **2018**, 3263–3278, doi: 10.2147/IJN.S166021.

12. Gao, W.; Xiang, B.; Meng, T.T.; Liu, F.; Qi, X.R. Chemotherapeutic drug delivery to cancer cells using a combination of folate targeting and tumour microenvironment-sensitive polypeptides. *Biomaterials* **2013**, *34*, 4137–4149, doi:10.1016/j.biomaterials.2013.02.014.

13. Liu, D.; Liu, Z.; Wang, L.; Zhang, C.; Zhang, N. Nanostructured lipid carriers as novel carrier for parenteral delivery of docetaxel. *Colloids Surfaces B Biointerfaces.* **2011**, *85*, 262–269, doi:10.1016/j.colsurfb.2011.02.038.

14. Fang, G.; Tang, B.; Chao, Y.; Xu, H.; Gou, J.; Zhang, Y.; Xu, H.; Tang, X. Cysteine-Functionalized Nanostructured Lipid Carriers for Oral Delivery of Docetaxel: A Permeability and Pharmacokinetic Study. *Mol. Pharm.* **2015**, *12*, 2384–2395, doi:10.1021/acs.molpharmaceut.5b00081.

15. Kharkar, P.B.; Talkar, S.S.; Patravale, V.B. An industrially viable technique for fabrication of docetaxel NLCs for oncotherapy. *Int. J. Pharm.* **2020**, *577*, 119082–119091, doi:10.1016/j.ijpharm.2020.119082.

16. Kim, C.H.; Kang, T.H.; Kim, B.D.; Lee, T.H.; Yoon, H.Y.; Goo, Y.T.; Choi, Y.S.; Kang, M.J.; Choi, Y.W. Enhanced docetaxel delivery using sterically stabilized RIPL peptide-conjugated nanostructured lipid carriers: In vitro and in vivo antitumour efficacy against SKOV3 ovarian cancer cells. *Int. J. Pharm.* **2020**, *583*, 119393–119404, doi:10.1016/j.ijpharm.2020.119393.

17. Li, X.; Wang, D.; Zhang, J.; Pan, W. Preparation and pharmacokinetics of docetaxel based on nanostructured lipid carriers. *J. Pharm. Pharmacol.* **2009**, *61*, 1485–1492, doi:10.1211/jpp/61.11.0007.

18. Li, M.; Pei, J.; Ma, Z.; Fu, J.; Chen, F.; Du, S. Docetaxel-loaded ultrasmall nanostructured lipid carriers for cancer therapy: in vitro and in vivo evaluation. *Cancer Chemother. Pharmacol.* **2020**, *85*, 731–739, doi:10.1007/s00280-020-04048-6.

19. Liu, D.; Liu, F.; Liu, Z.; Wang, L.; Zhang, N. Tumour specific delivery and therapy by double-targeted nanostructured lipid carriers with anti-VEGFR-2 antibody. *Mol. Pharm.* **2011**, *8*, 2291–2301, doi:10.1021/mp200402e.

20. Mathur, P.; Sharma, S.; Rawal, S.; Patel, B.; Patel, M.M. Fabrication, optimization, and in vitro evaluation of docetaxel-loaded nanostructured lipid carriers for improved anticancer activity. *J. Liposome Res.* **2020**, *30*, 182–196, doi:10.1080/08982104.2019.1614055.

21. Vohra, T.; Kaur, I.; Heer, H.; Murthy, R.R. Nanolipid carrier-based thermoreversible gel for localized delivery of docetaxel to breast cancer. *Cancer Nanotechnol.* **2013**, *4*, 1–12, doi:10.1007/s12645-013-0032-9.

22. Zhao, X.; Zhao, Y.; Geng, L.; Li, X.; Wang, X.; Liu, Z.; Wang, D.; Bi, K.; Chen, X. Pharmacokinetics and tissue distribution of docetaxel by liquid chromatography-mass spectrometry: Evaluation of folate receptor-targeting amphiphilic copolymer modified nanostructured lipid carrier. *J. Chromatogr. B.* **2011**, *879*, 3721–3727, doi:10.1016/j.jchromb.2011.10.015.

23. Zwain, T.; Alder, J.E.; Sabagh, B.; Shaw, A.; Burrow, A.J.; Singh, K.K. Tailoring functional nanostructured lipid carriers for glioblastoma treatment with enhanced permeability through in-vitro 3D BBB/BBTB models. *Mater. Sci. Eng. C* **2021**, *121*, 111774–111789, doi:10.1016/j.msec.2020.111774.

24. Fan, X.; Chen, J.; Shen, Q. Docetaxel-nicotinamide complex-loaded nanostructured lipid carriers for transdermal delivery. *Int. J. Pharm.* **2013**, *458*, 296–304, doi:10.1016/j.ijpharm.2013.10.036.

25. Bang, K.H.; Na, Y.G.; Huh, H.W.; Hwang, S.J.; Kim, M.S.; Kim, M.; Lee, H.K.; Cho, C.W. The delivery strategy of paclitaxel nanostructured lipid carrier coated with platelet membrane. *Cancers.* **2019**, *11*, 807–822, doi:10.3390/cancers11060807.

26. Chen, J.; Chen, H.; Cui, S.; Xue, B.; Tian, J.; Achilefu, S.; Gu, Y. Glucosamine derivative modified nanostructured lipid carriers for targeted tumour delivery. *J. Mater. Chem.* **2012**, *22*, 5770–5783, doi:10.1039/c2jm15830b.

27. Emami, J.; Rezazadeh, M.; Varshosaz, J.; Tabbakhian, M.; Aslani, A. Formulation of LDL targeted nanostructured lipid carriers loaded with paclitaxel: A detailed study of preparation, freeze drying condition, and in vitro cytotoxicity. *J. Nanomater.* **2012**, *2012*, 21–30, doi:10.1155/2012/358782.

28. Harshita; Barkat, M.A.; Rizwanullah, M.; Beg, S.; Pottoo, F.H.; Siddiqui, S.; Ahmad, F.J. Paclitaxel-loaded Nanolipidic Carriers with Improved Oral Bioavailability and Anticancer Activity against Human Liver Carcinoma. *AAPS PharmSciTech* **2019**, *20*, 87 doi:10.1208/s12249-019-1304-4.

29. Kaur, P.; Garg, T.; Rath, G.; Murthy, R.S.R.; Goyal, A.K. Development, optimization and evaluation of surfactant-based pulmonary nanolipid carrier system of paclitaxel for the management of drug resistance lung cancer using Box-Behnken design. *Drug Deliv.* **2016**, *23*, 1912–1925, doi:10.3109/10717544.2014.993486.

30. de Sousa Marcial, S.P.; Carneiro, G.; Leite, E.A. Lipid-based nanoparticles as drug delivery system for paclitaxel in breast cancer treatment. *J. Nanoparticle Res.* **2017**, *19*, 340 doi:10.1007/s11051-017-4042-0.

31. Sun, M.; Gao, Y.; Zhu, Z.; Wang, H.; Han, C.; Yang, X.; Pan, W. A systematic in vitro investigation on poly-arginine modified nanostructured lipid carrier: Pharmaceutical characteristics, cellular uptake, mechanisms and cytotoxicity. *Asian J. Pharm. Sci.* **2017**, *12*, 51–58, doi:10.1016/j.ajps.2016.07.007.

32. Ucar, E.; Teksoz, S.; Ichedef, C.; Kilcar, A.Y.; Medine, E.I.; Ari, K.; Parlak, Y.; Sayit Bilgin, B.E.; Unak, P. Synthesis, characterization and radiolabeling of folic acid modified nanostructured lipid carriers as a contrast agent and drug delivery system. *Appl. Radiat. Isot.* **2017**, *119*, 72–79, doi:10.1016/j.apradiso.2016.11.002.

33. Yang, X. yan; Li, Y. xia; Li, M.; Zhang, L.; Feng, L. xia; Zhang, N. Hyaluronic acid-coated nanostructured lipid carriers for targeting paclitaxel to cancer. *Cancer Lett.* **2013**, *334*, 338–345, doi:10.1016/j.canlet.2012.07.002.

34. Zhao, X.; Tang, D.; Yang, T.; Wang, C. Facile preparation of biocompatible nanostructured lipid carrier with ultra-small size as a tumour-penetration delivery system. *Colloids Surfaces B Biointerfaces* 2018, *170*, 355–363, doi: 10.1016/j.colsurfb.2018.06.017.

35. Zhou, J.; Guo, B.; Zhu, W.; Sui, X.; Ma, X.; Qian, J.; Cao, L.; Han, C. Novel biomimetic nanostructured lipid carriers for cancer therapy: preparation, characterization, and in vitro/in vivo evaluation. *Pharm. Dev. Technol.* **2021**, *26*, 81–91, doi:10.1080/10837450.2020.1835957.

36. Emami, J.; Rezazadeh, M.; Sadeghi, H.; Khadivar, K. Development and optimization of transferrin-conjugated nanostructured lipid carriers for brain delivery of paclitaxel using Box–Behnken design. *Pharm. Dev. Technol.* **2017**, *22*, 370–382, doi:10.1080/10837450.2016.1189933.

37. Gao, X.; Zhang, J.; Xu, Q.; Huang, Z.; Wang, Y.; Shen, Q. Hyaluronic acid-coated cationic nanostructured lipid carriers for oral vincristine sulfate delivery. *Drug Dev. Ind. Pharm.* **2017**, *43*, 661–667, doi:10.1080/03639045.2016.1275671.

38. Zhang, G.; Liu, F.; Jia, E.; Jia, L.; Zhang, Y. Folate-modified , cisplatin-loaded lipid carriers for cervical cancer chemotherapy. *Drug Deliv.* **2016**, *23*, 1393–1397, doi:10.3109/10717544.2015.1054052.

39. Zhang, Y.; Zhang, P.; Zhu, T. Ovarian carcinoma biological nanotherapy : Comparison of the advantages and drawbacks of lipid, polymeric and hybrid nanoparticles for cisplatin delivery. *Biomed. Pharmacother.* **2019**, *109*, 475–483, doi:10.1016/j.biopha.2018.10.158.

40. Almoussalam, M.; Zhu, H. Encapsulation of Cancer Therapeutic Agent Dacarbazine Using Nanostructured Lipid Carrier. **2016**, 53760 doi:10.3791/53760.

41. Velmurugan, R.; Selvamuthukumar, S. Development and optimization of ifosfamide nanostructured lipid carriers for oral delivery using response surface methodology. *Appl. Nanosci.* **2016**, *6*, 159–173, doi:10.1007/s13204-015-0434-6.

42. Velmurugan, R.; Selvamuthukumar, S. In Vivo Antitumour Activity of a Novel Orally Bioavailable Ifosfamide Nanostructured Lipid Carrier Against Dalton's Ascitic Lymphoma. *J. Pharm. Innov.* **2014**, *9*, 203–211, doi:10.1007/s12247-014-9187-0.

43. Velmurugan, R.; Nair, K.G.S. Toxicity evaluation of ifosfamide nanostructured lipid carriers designed for oral delivery in Wistar albino rats. *Drug Invent. Today*. **2018**, *10*, 192–196

44. Gidwani, B.; Vyas, A.; Kaur, C.D. Cytotoxicity and pharmacokinetics study of nanostructured lipid carriers of mechlorethamine: Preparation, optimization and characterization. *Part. Sci. Technol.* **2020**, *38*, 23–33, doi:10.1080/02726351.2018.1536685.

45. Khan, A.; Imam, S.S.; Aqil, M.; Ahad, A.; Sultana, Y.; Ali, A.; Khan, K. Brain Targeting of Temozolomide via the Intranasal Route Using Lipid-Based Nanoparticles: Brain Pharmacokinetic and Scintigraphic Analyses. *Mol. Pharmaceutics.* **2016**, *13*, 3773-3782, doi:10.1021/acs.molpharmaceut.6b00586.

46. Qu, J.; Zhang, L.; Chen, Z.; Mao, G.; Gao, Z.; Lai, X.; Zhu, X.; Zhu, J. Nanostructured lipid carriers, solid lipid nanoparticles, and polymeric nanoparticles: which kind of drug delivery system is better for glioblastoma chemotherapy? *Drug Deliv.* **2016**, *23*, 3408–3416, doi:10.1080/10717544.2016.1189465.

47. Chen, Z.; Lai, X.; Song, S.; Zhu, X.; Zhu, J. Nanostructured lipid carriers based temozolomide and gene co-encapsulated nanomedicine for gliomatosis cerebri combination therapy. *Drug Deliv.* **2016**, *23*, 1369–1373, doi:10.3109/10717544.2015.1038857.

48. Song, S.; Mao, G.; Du, J.; Zhu, X. Novel RGD containing, temozolomide-loading nanostructured lipid carriers for glioblastoma multiforme chemotherapy. *Drug Deliv.* **2016**, *23*, 1404–1408, doi:10.3109/10717544.2015.1064186.

49. Raeisi, S.; Chavoshi, H.; Mohammadi, M.; Ghorbani, M.; Sabzichi, M.; Ramezani, F. Naringenin-loaded nano-structured lipid carrier fortifies oxaliplatin-dependent apoptosis in HT-29 cell line. *Process Biochem.* **2019**, *83*, 168–175, doi:10.1016/j.procbio.2019.05.013.

50. Abdolahpour, S.; Mahdieh, N.; Jamali, Z.; Akbarzadeh, A.; Toliyat, T.; Paknejad, M. Development of Doxorubicin-Loaded Nanostructured Lipid Carriers: Preparation, Characterization, and In Vitro Evaluation on MCF-7 Cell Line. *Bionanoscience* **2017**, *7*, 32–39, doi:10.1007/s12668-016-0391-x.

51. Fernandes, R.S.; Silva, J.O.; Monteiro, L.O.F.; Leite, E.A.; Cassali, G.D.; Rubello, D.; Cardoso, V.N.; Ferreira, L.A.M.; Oliveira, M.C.; de Barros, A.L.B. Doxorubicin-loaded nanocarriers: A comparative study of liposome and nanostructured lipid carrier as alternatives for cancer therapy. *Biomed. Pharmacother.* **2016**, *84*, 252–257, doi:10.1016/j.biopha.2016.09.032.

52. Fernandes, R.S.; Silva, J.O.; Mussi, S. V.; Lopes, S.C.A.; Leite, E.A.; Cassali, G.D.; Cardoso, V.N.; Townsend, D.M.; Colletti, P.M.; Ferreira, L.A.M.; et al. Nanostructured Lipid Carrier Co-loaded with Doxorubicin and Docosahexaenoic Acid as a Theranostic Agent: Evaluation of Biodistribution and Antitumour Activity in Experimental Model. *Mol. Imaging Biol.* **2018**, *20*, 437–447, doi:10.1007/s11307-017-1133-3.

53. Fernandes, R.S.; Silva, J.O.; Seabra, H.A.; Oliveira, M.S.; Carregal, V.M.; Vilela, J.M.C.; Andrade, M.S.; Townsend, D.M.; Colletti, P.M.; Leite, E.A.; Cardoso, V.N.; Ferreira, L.A.M.; Rubello D.; Barros, A.L.B. α- Tocopherol succinate loaded nano-structed lipid carriers improves antitumour activity of doxorubicin in breast cancer models in vivo. *Biomed. Pharmacother.* **2018**, *103*, 1348–1354, doi:10.1016/j.biopha.2018.04.139.

54. Hajipour, H.; Ghorbani, M.; Kahroba, H.; Mahmoodzadeh, F.; Emameh, R.Z.; Taheri, R.A. Arginyl-glycyl-aspartic acid (RGD) containing

nanostructured lipid carrier co-loaded with doxorubicin and sildenafil citrate enhanced anti-cancer effects and overcomes drug resistance. *Process Biochem.* **2019**, *84*, 172–179, doi:10.1016/j.procbio.2019.06.013.

55. Han, Y.; Zhang, Y.; Li, D.; Chen, Y.; Sun, J.; Kong, F. Transferrin-modified nanostructured lipid carriers as multifunctional nanomedicine for codelivery of DNA and doxorubicin. *Int. J. Nanomedicine* **2014**, *9*, 4107–4116, doi:10.2147/IJN.S67770.

56. Lages, E.B.; Fernandes, R.S.; Silva, J. de O.; de Souza, Â.M.; Cassali, G.D.; de Barros, A.L.B.; Miranda Ferreira, L.A. Co-delivery of doxorubicin, docosahexaenoic acid, and α-tocopherol succinate by nanostructured lipid carriers has a synergistic effect to enhance antitumour activity and reduce toxicity. *Biomed. Pharmacother.* **2020**, *132*, 110876–110887, doi:10.1016/j.biopha.2020.110876.

57. Li, W.; Fu, J.; Ding, Y.; Liu, D.; Jia, N.; Chen, D.; Hu, H. Low density lipoprotein-inspired nanostructured lipid nanoparticles containing pro-doxorubicin to enhance tumour-targeted therapeutic efficiency. *Acta Biomater.* **2019**, *96*, 456–467, doi:10.1016/j.actbio.2019.06.051.

58. Mohammadian, J.; Mahmoudi, S.; Pourmohammad, P.; Pirouzpanah, M.; Salehnia, F.; Maroufi, N.F.; Samadi, N.; Sabzichi, M. Formulation of Stattic as STAT3 inhibitor in nanostructured lipid carriers (NLCs) enhances efficacy of doxorubicin in melanoma cancer cells. *Naunyn. Schmiedebergs. Arch. Pharmacol.* **2020**, *393*, 2315–2323, doi:10.1007/s00210-020-01942-x.

59. Moraes, S.; Marinho, A.; Lima, S.; Granja, A.; Araújo, J.P.; Reis, S.; Sousa, C.T.; Nunes, C. Targeted nanostructured lipid carriers for doxorubicin oral delivery. *Int. J. Pharm.* **2021**, *592*, 120029–120039, doi:10.1016/j.ijpharm.2020.120029.

60. Mussi, S. V.; Sawant, R.; Perche, F.; Oliveira, M.C.; Azevedo, R.B.; Ferreira, L.A.M.; Torchilin, V.P. Novel nanostructured lipid carrier co-loaded with doxorubicin and docosahexaenoic acid demonstrates enhanced in vitro activity and overcomes drug resistance in MCF-7/Adr cells. *Pharm. Res.* **2014**, *31*, 1882–1892, doi:10.1007/s11095-013-1290-2.

61. Mussi, S. V.; Parekh, G.; Pattekari, P.; Levchenko, T.; Lvov, Y.; Ferreira, L.A.M.; Torchilin, V.P. Improved pharmacokinetics and enhanced tumour growth inhibition using a nanostructured lipid carrier loaded with doxorubicin and modified with a layer-by-layer polyelectrolyte coating. *Int. J. Pharm.* **2015**, *495*, 186–193, doi:10.1016/j.ijpharm.2015.08.079.

62. Sabzichi, M.; Mohammadian, J.; Mohammadi, M.; Jahanfar, F.; Movassagh Pour, A.A.; Hamishehkar, H.; Ostad-Rahimi, A. Vitamin D-Loaded Nanostructured Lipid Carrier (NLC): A New Strategy for Enhancing Efficacy of Doxorubicin in Breast Cancer Treatment. *Nutr. Cancer* **2017**, *69*, 840–848, doi:10.1080/01635581.2017.1339820.

63. Zhang, H.W.; Dang, Q.; Zhang, Z.W.; Wu, F.S. Development, characterization and evaluation of doxorubicin nanostructured lipid carriers for prostate cancer. *J. BUON.* **2017**, *22*, 102–111.

64. Zhao, S.; Minh, L. Van; Li, N.; Garamus, V.M.; Handge, U.A.; Liu, J.; Zhang, R.; Willumeit-Römer, R.; Zou, A. Doxorubicin hydrochloride-oleic acid conjugate loaded nanostructured lipid carriers for tumour specific drug release. *Colloids Surfaces B Biointerfaces* **2016**, *145*, 95–103,

doi:10.1016/j.colsurfb.2016.04.027.

65. Deng, C.; Jia, M.; Wei, G.; Tan, T.; Fu, Y.; Gao, H.; Sun, X.; Zhang, Q.; Gong, T.; Zhang, Z. Inducing Optimal Antitumour Immune Response through Coadministering iRGD with Pirarubicin Loaded Nanostructured Lipid Carriers for Breast Cancer Therapy. *Mol. Pharm.* **2017**, *14*, 296–309, doi:10.1021/acs.molpharmaceut.6b00932.

66. Negi, L.M.; Talegaonkar, S.; Jaggi, M.; Verma, A.K.; Verma, R.; Dobhal, S.; Kumar, V. Surface engineered nanostructured lipid carriers for targeting MDR tumour: Part II. In vivo biodistribution, pharmacodynamic and hematological toxicity studies. *Colloids Surfaces B Biointerfaces* **2014**, *123*, 610–615, doi:10.1016/j.colsurfb.2014.09.061

67. Negi, L.M.; Talegaonkar, S.; Jaggi, M.; Verma, A.K.; Verma, R.; Dobhal, S.; Kumar, V. Surface engineered nanostructured lipid carriers for targeting MDR tumour: Part I. Synthesis, characterization and in vitro investigation. *Colloids Surfaces B Biointerfaces* **2014**, *123*, 600–609, doi:10.1016/J.COLSURFB.2014.09.062.

68. Jiang, H.; Pei, L.; Liu, N.; Li, J.; Li, Z.; Zhang, S. Etoposide-loaded nanostructured lipid carriers for gastric cancer therapy. *Drug Deliv.* **2016**, *23*, 1379–1382, doi:10.3109/10717544.2015.1048491.

69. Khajavinia, A.; Varshosaz, J.; Dehkordi, A.J. Targeting etoposide to acute myelogenous leukaemia cells using nanostructured lipid carriers coated with transferrin. *Nanotechnology* **2012**, *23*, 405101–405114, doi:10.1088/0957-4484/23/40/405101.

70. Wang, H.; Sun, G.; Zhang, Z.; Ou, Y. Transcription activator, hyaluronic acid and tocopheryl succinate multi-functionalized novel lipid carriers encapsulating etoposide for lymphoma therapy. *Biomed. Pharmacother.* **2017**, *91*, 241–250, doi:10.1016/j.biopha.2017.04.104.

71. Zhang, T.; Chen, J.; Zhang, Y.; Shen, Q.; Pan, W. Characterization and evaluation of nanostructured lipid carrier as a vehicle for oral delivery of etoposide. *Eur. J. Pharm. Sci.* **2011**, *43*, 174–179, doi:10.1016/j.ejps.2011.04.005.

72. Zhang, S.; Lu, C.; Zhang, X.; Li, J.; Jiang, H. Targeted delivery of etoposide to cancer cells by folate-modified nanostructured lipid drug delivery system. *Drug Deliv.* **2016**, *23*, 1838–1845, doi:10.3109/10717544.2016.1141258.

73. Venâncio, J.H.; Andrade, L.M.; Esteves, N.L.S.; Brito, L.B.; Valadares, M.C.; Oliveira, G.A.R.; Lima, E.M.; Marreto, R.N.; Gratieri, T.; Taveira, S.F. Topotecan-loaded lipid nanoparticles as a viable tool for the topical treatment of skin cancers. *J. Pharm. Pharmacol.* **2017**, *69*, 1318–1326, doi:10.1111/jphp.12772.

74. Kaur, P.; Mishra, V.; Shunmugaperumal, T.; Goyal, A.K.; Ghosh, G.; Rath, G. Inhalable spray dried lipidnanoparticles for the co-delivery of paclitaxel and doxorubicin in lung cancer. *J. Drug Deliv. Sci. Technol.* **2020**, *56*, 101502–101511, doi:10.1016/j.jddst.2020.101502.

75. Wang, Y.; Zhang, H.; Hao, J.; Li, B.; Li, M.; Xiuwen, W. Lung cancer combination therapy: co-delivery of paclitaxel and doxorubicin by nanostructured lipid carriers for synergistic effect. *Drug Deliv.* **2016**, *23*, 1398–1403, doi:10.3109/10717544.2015.1055619.

76. Zhang, X.G.; Miao, J.; Dai, Y.Q.; Du, Y.Z.; Yuan, H.; Hu, F.Q. Reversal activity of nanostructured lipid carriers loading cytotoxic drug in multi-drug resistant cancer cells. *Int. J. Pharm.* **2008**, *361*, 239–244, doi:10.1016/j.ijpharm.2008.06.002.

77. Raikwar, S.; Vyas, S.; Sharma, R.; Mody, N.; Dubey, S.; Vyas, S.P. Nanocarrier-Based Combination Chemotherapy for Resistant Tumour: Development, Characterization, and Ex Vivo Cytotoxicity Assessment. *AAPS PharmSciTech* **2018**, *19*, 3839–3849, doi:10.1208/s12249-018-1185-y.

78. Chang, L.; Zhang, Y.; Li, M.; Zhao, X.; Wang, D.; Liu, J.; Zhou, F.; Zhang, J. Nanostructured lipid carrier co-delivering paclitaxel and doxorubicin restrains the proliferation and promotes apoptosis of glioma stem cells via regulating PI3K/Akt/mTOR signaling. *Nanotechnology* **2021**, *32*, 225101–225113, doi:10.1088/1361-6528/abd439.

79. Di, H.; Wu, H.; Gao, Y.; Li, W.; Zou, D.; Dong, C. Doxorubicin- and cisplatin-loaded nanostructured lipid carriers for breast cancer combination chemotherapy. *Drug Dev. Ind. Pharm.* **2016**, *42*, 2038–2043, doi:10.1080/03639045.2016.1190743.

80. Dong, X.; Wang, W.; Qu, H.; Han, D.; Zheng, J.; Sun, G. Targeted delivery of doxorubicin and vincristine to lymph cancer: evaluation of novel nanostructured lipid carriers in vitro and in vivo. *Drug Deliv.* **2016**, *23*, 1374–1378, doi:10.3109/10717544.2015.1041580.

81. Ni, S.; Qiu, L.; Zhang, G.; Zhou, H.; Han, Y. Lymph cancer chemotherapy: Delivery of doxorubicin-gemcitabine prodrug and vincristine by nanostructured lipid carriers. *Int. J. Nanomedicine* **2017**, *12*, 1565–1576, doi:10.2147/IJN.S120685.

82. Liu, Q.; Li, J.; Pu, G.; Zhang, F.; Liu, H.; Zhang, Y. Co-delivery of baicalein and doxorubicin by hyaluronic acid decorated nanostructured lipid carriers for breast cancer therapy. *Drug Deliv.* **2016**, *23*, 1364–1368, doi:10.3109/10717544.2015.1031295.

83. Borges, G.S.M.; Silva, J. de O.; Fernandes, R.S.; de Souza, Â.M.; Cassali, G.D.; Yoshida, M.I.; Leite, E.A.; de Barros, A.L.B.; Ferreira, L.A.M. Sclareol is a potent enhancer of doxorubicin: Evaluation of the free combination and co-loaded nanostructured lipid carriers against breast cancer. *Life Sci.* **2019**, *232*, 116678–116690, doi:10.1016/j.lfs.2019.116678.

84. Cao, C.; Wang, Q.; Liu, Y. Lung cancer combination therapy: Doxorubicin and β-elemene co-loaded, pH-sensitive nanostructured lipid carriers. *Drug Des. Devel. Ther.* **2019**, *13*, 1087–1098, doi:10.2147/DDDT.S198003.

85. Li, X.; Jia, X.; Niu, H. Nanostructured lipid carriers co-delivering lapachone and doxorubicin for overcoming multidrug resistance in breast cancer therapy. *Int. J. Nanomedicine* **2018**, *13*, 4107–4119, doi:10.2147/IJN.S163929.

86. Yang, J.; Ju, Z.; Dong, S. Cisplatin and paclitaxel co-delivered by folate-decorated lipid carriers for the treatment of head and neck cancer. *Drug Deliv.* **2017**, *24*, 792–799, doi:10.1080/10717544.2016.1236849.

87. Guo, S.; Zhang, Y.; Wu, Z.; Zhang, L.; He, D.; Li, X.; Wang, Z. Synergistic combination therapy of lung cancer: Cetuximab functionalized nanostructured lipid carriers for the co-delivery of paclitaxel and 5-Demethylnobiletin. *Biomed. Pharmacother.* **2019**, *118*, 109225–109232, doi:10.1016/j.biopha.2019.109225.

88. Qu, C.-Y.Y.; Zhou, M.; Chen, Y.W.; Chen, M.M.; Shen, F.; Xu, L.-M.M. Engineering of lipid prodrug-based, hyaluronic acid-decorated nanostructured lipid carriers platform for 5-fluorouracil and cisplatin combination gastric cancer therapy. *Int. J. Nanomedicine* **2015**, *10*, 3911–3920, doi:10.2147/IJN.S83211.

89. Wu, M.; Fan, Y.; Lv, S.; Xiao, B.; Ye, M.; Zhu, X. Vincristine and temozolomide combined chemotherapy for the treatment of glioma: a comparison of solid lipid nanoparticles and nanostructured lipid carriers for dual drugs delivery. *Drug Deliv.* **2016**, *23*, 2720–2725, doi:10.3109/10717544.2015.1058434.

90. Zhang, J.; Xiao, X.; Zhu, J.; Gao, Z.; Lai, X.; Zhu, X.; Mao, G. Lactoferrin- and RGD-comodified, temozolomide and vincristine-coloaded nanostructured lipid carriers for gliomatosis cerebri combination therapy. *Int. J. Nanomedicine* **2018**, *13*, 3039–3051, doi:10.2147/IJN.S161163.

91. Liang, Y.; Zhang, J.; Tian, B.; Wu, Z.; Svirskis, D.; Han, J. A NAG-guided nano-delivery system for redox-and pH-triggered intracellularly sequential drug release in cancer cells. *Int. J. Nanomedicine* **2020**, *15*, 841–855, doi:10.2147/IJN.S226249.

92. Liang, Y.; Tian, B.; Zhang, J.; Li, K.; Wang, L.; Han, J.; Wu, Z. Tumour-targeted polymeric nanostructured lipid carriers with precise ratiometric control over dual-drug loading for combination therapy in non-small-cell lung cancer. *Int. J. Nanomedicine* **2017**, *12*, 1699–1715, doi:10.2147/IJN.S121262.

93. Lu, Z.; Su, J.; Li, Z.; Zhan, Y.; Ye, D. Hyaluronic acid-coated, prodrug-based nanostructured lipid carriers for enhanced pancreatic cancer therapy. *Drug Dev. Ind. Pharm.* **2017**, *43*, 160–170, doi:10.1080/03639045.2016.1226337.

94. Zhou, J.; Sun, M.; Jin, S.; Fan, L.; Zhu, W.; Sui, X.; Cao, L.; Yang, C.; Han, C. Combined using of paclitaxel and salinomycin active targeting nanostructured lipid carriers against non-small cell lung cancer and cancer stem cells. *Drug Deliv.* **2019**, *26*, 281–289, doi:10.1080/10717544.2019.1580799.

95. Taratula, O.; Kuzmov, A.; Shah, M.; Garbuzenko, O.B.; Minko, T. Nanostructured lipid carriers as multifunctional nanomedicine platform for pulmonary co-delivery of anticancer drugs and siRNA. *J. Control. Release* **2013**, *171*, 349–357, doi:10.1016/j.jconrel.2013.04.018.

96. Shao, Z.; Shao, J.; Tan, B.; Guan, S.; Liu, Z.; Zhao, Z.; He, F.; Zhao, J. Targeted lung cancer therapy: Preparation and optimization of transferrin-decorated nanostructured lipid carriers as novel nanomedicine for co-delivery of anticancer drugs and DNA. *Int. J. Nanomedicine* **2015**, *10*, 1223–1233, doi:10.2147/IJN.S77837.

97. Wang, J.Y.; Song, Y.Q.; Peng, J.; Luo, H.L. Nanostructured lipid carriers delivering sorafenib to enhance immunotherapy induced by doxorubicin for effective esophagus cancer therapy. *ACS Omega* **2020**, *5*, 22840–22846, doi:10.1021/acsomega.0c02072.

98. Mahoutforoush, A.; Solouk, A.; Hamishehkar, H.; Haghbin Nazarpak, M.; Abbaspour-Ravasjani, S. Novel decorated nanostructured lipid carrier for simultaneous active targeting of three anti-cancer agents. *Life Sci.* **2021**, *279*, 119576–119589, doi:10.1016/J.LFS.2021.119576.

99. Varshosaz, J.; Davoudi, M.A.; Rasoul-Amini, S. Docetaxel-loaded nanostructured lipid carriers functionalized with trastuzumab (Herceptin) for HER2-

positive breast cancer cells. *J. Liposome Res.* **2018**, *28*, 285–295, doi:10.1080/08982104.2017.1370471.

100. Maroufi, N.F.; Vahedian, V.; Mazrakhondi, S.A.M.; Kooti, W.; Khiavy, H.A.; Bazzaz, R.; Ramezani, F.; Pirouzpanah, S.M.; Ghorbani, M.; Akbarzadeh, M.; et al. Sensitization of MDA-MBA231 breast cancer cell to docetaxel by myricetin loaded into biocompatible lipid nanoparticles via sub-G1 cell cycle arrest mechanism. *Naunyn. Schmiedebergs. Arch. Pharmacol.* **2020**, *393*, 1–11, doi:10.1007/s00210-019-01692-5.

101. Bondì, M.L.; Emma, M.R.; Botto, C.; Augello, G.; Azzolina, A.; Gaudio, F. Di; Craparo, E.F.; Cavallaro, G.; Bachvarov, D.; Cervello, M. Biocompatible Lipid Nanoparticles as Carriers To Improve Curcumin Efficacy in Ovarian Cancer Treatment. *J. Agric. Food Chem.* **2017**, *65*, 1342–1352, doi:10.1021/ACS.JAFC.6B04409.

102. Bondì, M.; Craparo, E.; Picone, P.; Carlo, M.; Gesu, R.; Capuano, G.; Giammona, G. Curcumin Entrapped Into Lipid Nanosystems Inhibits Neuroblastoma Cancer Cell Growth and Activates Hsp70 Protein. *Curr. Nanosci.* **2010**, *6*, 439–445, doi:10.2174/157341310797575005.

103. Wang, F.; Chen, J.; Dai, W.; He, Z.; Zhai, D.; Chen, W. Pharmacokinetic studies and anticancer activity of curcumin-loaded nanostructured lipid carriers. *Acta Pharm.* **2017**, *67*, 357–371, doi:10.1515/acph-2017-0021.

104. Wang, F.; Ye, X.; Zhai, D.; Dai, W.; Wu, Y.; Chen, J.; Chen, W. Curcumin-loaded nanostructured lipid carrier induced apoptosis in human HepG2 cells through activation of the DR5/caspase-mediated extrinsic apoptosis pathway. *Acta Pharm.* **2020**, *70*, 227–237, doi:10.2478/ACPH-2020-0003.

105. Karami, S.; Rostamizadeh, K.; Shademani, N.; Parsa, M. Synthesis and Investigation of the Curcumin-Loaded Magnetic Lipid Nanoparticles and Their Cytotoxicity Assessment on Human Breast Carcinoma Cell Line. *Jundishapur J. Nat. Pharm. Prod.* **2020**, *15*, doi:10.5812/JJNPP.91886.

106. Chen, Y.; Pan, L.; Jiang, M.; Li, D.; Jin, L. Nanostructured lipid carriers enhance the bioavailability and brain cancer inhibitory efficacy of curcumin both in vitro and in vivo. *Drug Deliv.* **2015**, *23*,1383–1392, doi:10.3109/10717544.2015.1049719.

107. Kamel, A.E.; Fadel, M.; Louis, D. Curcumin-loaded nanostructured lipid carriers prepared using Peceol™ and olive oil in photodynamic therapy: development and application in breast cancer cell line. *Int J. Nanomedicine* **2019**, *14,* 5073–5085, doi:10.2147/IJN.S210484.

108. Varshosaz, J.; Jandaghian, S.; Mirian, M.; Sajjadi, S.E. Co-delivery of rituximab targeted curcumin and imatinib nanostructured lipid carriers in non-Hodgkin lymphoma cells. *J. Liposome Res.* **2020**, *31*, 64–78, doi:10.1080/08982104.2020.1720718.

109. Rawal, S.; Patel, B.; Patel, M.M. Fabrication, optimisation and in vitro evaluation of docetaxel and curcumin Co-loaded nanostructured lipid carriers for improved antitumour activity against non-small cell lung carcinoma. *J. Microencapsul.* **2020**, *37*, 543–556, doi:10.1080/02652048.2020.1823498.

110. Rawal, S.; Bora, V.; Patel, B.; Patel, M. Surface-engineered nanostructured lipid carrier systems for synergistic combination oncotherapy of non-small cell lung cancer. *Drug Deliv. Transl. Res.* **2020**, *11, 2030* –2051, doi:10.1007/s13346-020-00866-6.

111. Xu, M.; Li, G.; Zhang, H.; Chen, X.; Li, Y. Sequential delivery of dual drugs with nanostructured lipid carriers for improving synergistic tumour

treatment effect. *Drug Deliv.* **2020**, *27*, 983-995,doi:10.1080/10717544.2020.1785581.

112. Almeida, E.D.P.; Dipieri, L. V.; Rossetti, F.C.; Marchetti, J.M.; Bentley, M.V.L.B.; Nunes, R. de S.; Sarmento, V.H. V.; Valerio, M.E.G.; Rodrigues Júnior, J.J.; Montalvão, M.M.; et al. Skin permeation, biocompatibility and antitumour effect of chloroaluminum phthalocyanine associated to oleic acid in lipid nanoparticles. *Photodiagnosis Photodyn. Ther.* **2018**, *24*, 262–273, doi:10.1016/j.pdpdt.2018.10.002.

113. Chen, G.; Wang, K.; Zhou, Y.; Ding, L.; Ullah, A.; Hu, Q.; Sun, M.; Oupický, D. Oral Nanostructured Lipid Carriers Loaded with Near-Infrared Dye for Image-Guided Photothermal Therapy. *ACS Appl. Mater. Interfaces* **2016**, *8*, 25087–25095, doi:10.1021/acsami.6b07425.

114. Li, H.; Wang, K.; Yang, X.; Zhou, Y.; Ping, Q.; Oupicky, D.; Sun, M. Dual-function nanostructured lipid carriers to deliver IR780 for breast cancer treatment: Anti-metastatic and photothermal anti-tumour therapy. *Acta Biomater.* **2017**, *53*, 399–413, doi:10.1016/j.actbio.2017.01.070.

115. Michy; Massias; Bernard; Vanwonterghem; Henry; Guidetti; Royal; Coll; Texier; Josserand; et al. Verteporfin-Loaded Lipid Nanoparticles Improve Ovarian Cancer Photodynamic Therapy In Vitro and In Vivo. *Cancers (Basel).* **2019**, *11*, 1760–1778, doi:10.3390/cancers11111760.

116. Oshiro-Junior, J.A.; Sato, M.R.; Boni, F.I.; Santos, K.L.M.; de Oliveira, K.T.; de Freitas, L.M.; Fontana, C.R.; Nicholas, D.; McHale, A.; Callan, J.F.; et al. Phthalocyanine-loaded nanostructured lipid carriers functionalized with folic acid for photodynamic therapy. *Mater. Sci. Eng. C* **2020**, *108*, 110462–110468, doi:10.1016/j.msec.2019.110462.

117. Qidwai, A.; Khan, S.; Md, S.; Fazil, M.; Baboota, S.; Narang, J.K.; Ali, J. Nanostructured lipid carrier in photodynamic therapy for the treatment of basal-cell carcinoma. *Drug Deliv.* **2016**, *23*, 1476–1485, doi:10.3109/10717544.2016.1165310.

118. Wang, K.; Zhang, J.; de Sousa Júnior, W.T.; da Silva, V.C.M.; Rodrigues, M.C.; Morais, J.A.V.; Jiang, C.; Longo, J.P.F.; Azevedo, R.B.; Muehlmann, L.A. A xanthene derivative, free or associated to nanoparticles, as a new potential agent for anticancer photodynamic therapy. *J. Biomater. Sci. Polym. Ed.* **2020**, 31, 1977-1993, doi:10.1080/09205063.2020.1788370.

119. Zhang, Q.; Zhao, J.; Hu, H.; Yan, Y.; Hu, X.; Zhou, K.; Xiao, S.; Zhang, Y.; Feng, N. Construction and in vitro and in vivo evaluation of folic acid-modified nanostructured lipid carriers loaded with paclitaxel and chlorin e6. *Int. J. Pharm.* **2019**, *569*, 118595–118606, doi:10.1016/j.ijpharm.2019.118595.

120. Xuefang Ding, Xian Xu, Ye Zhao, Lihui Zhang, Yadong Yu, Fei Huang, Dezhou Yin, He Huang. Tumour targeted nanostructured lipid carrier co-delivering paclitaxel and indocyanine green for laser triggered synergetic therapy of cancer. *RSC Advances* **2017**, *7,* 35086–35095 doi:10.1039/C7RA06119
